# A model for brain life history evolution

**DOI:** 10.1371/journal.pcbi.1005380

**Published:** 2017-03-09

**Authors:** Mauricio González-Forero, Timm Faulwasser, Laurent Lehmann

**Affiliations:** 1 Department of Ecology and Evolution, University of Lausanne, Lausanne, Switzerland; 2 Laboratoire d’Automatique, École Polytechnique Fédérale de Lausanne, Lausanne, Switzerland; 3 Institute for Applied Computer Science, Karlsruhe Institute of Technology, Eggenstein-Leopoldshafen, Baden-Württemberg, Germany; CNRS, FRANCE

## Abstract

Complex cognition and relatively large brains are distributed across various taxa, and many primarily verbal hypotheses exist to explain such diversity. Yet, mathematical approaches formalizing verbal hypotheses would help deepen the understanding of brain and cognition evolution. With this aim, we combine elements of life history and metabolic theories to formulate a metabolically explicit mathematical model for brain life history evolution. We assume that some of the brain’s energetic expense is due to production (learning) and maintenance (memory) of energy-extraction skills (or cognitive abilities, knowledge, information, etc.). We also assume that individuals use such skills to extract energy from the environment, and can allocate this energy to grow and maintain the body, including brain and reproductive tissues. The model can be used to ask what fraction of growth energy should be allocated at each age, given natural selection, to growing brain and other tissues under various biological settings. We apply the model to find uninvadable allocation strategies under a baseline setting (“me vs nature”), namely when energy-extraction challenges are environmentally determined and are overcome individually but possibly with maternal help, and use modern-human data to estimate model’s parameter values. The resulting uninvadable strategies yield predictions for brain and body mass throughout ontogeny and for the ages at maturity, adulthood, and brain growth arrest. We find that: (1) a me-vs-nature setting is enough to generate adult brain and body mass of ancient human scale and a sequence of childhood, adolescence, and adulthood stages; (2) large brains are favored by intermediately challenging environments, moderately effective skills, and metabolically expensive memory; and (3) adult skill is proportional to brain mass when metabolic costs of memory saturate the brain metabolic rate allocated to skills.

## Author summary

Complex cognition and relatively large brains occur in a diversity of mammal, bird, and fish species among others, and there is a large number of mostly verbal hypotheses to explain what causes their evolution in certain species but not others. However, these hypotheses have scarcely exploited the power of formulating them in mathematical terms, which has been very useful to understand the evolution of other traits. To address this issue, we formulate a mathematical model that allows incorporating many of those hypotheses and that can be used to obtain predictions for how much and how fast the brain should grow depending on the hypothesis assumed. We apply the model to humans in a setting where each individual must extract energy from the environment alone (e.g., by hunting or cooking) but possibly with its mother’s help when young (“me vs nature”). We find that a me-vs-nature setting can be enough to produce a variety of human features, including large brain sizes and an adolescence life stage. Our model can be used to compare hypotheses promoting brain evolution, such as harsh environments or plentiful social interactions.

## Introduction

Complex cognitive abilities and relatively large brains are distributed across a variety of taxa [1], and there is a large number of primarily verbal hypotheses proposed to explain such diversity. Leading hypotheses suggest that various ecological and social challenges favor enhanced cognition or relatively large brains [1–9]. Empirical tests for these hypotheses regularly involve assessment of correlations, for instance, between diet quality or group size with cognitive ability or proxies thereof [10–17]. A complementary approach has been via functional studies; for example, behavioral experiments in humans find refined cognitive skills for social rather than general function [6, 18], and brain imaging also in humans has identified various brain regions specialized for social interaction [19, 20]. More recently, studies have addressed more directly the causes of large-brain evolution via phylogenetic analyses, artificial selection experiments, and genomic patterns of selection [21–25]. However, as hypotheses have remained mostly verbal, understanding of brain evolution may benefit from mathematical formalization to be used in synergy with empirical research, which can reveal features that are otherwise difficult to identify given biological complexity [26, 27]. In addition, mathematical models that can make quantitative rather than essentially qualitative predictions could facilitate obtaining contrasting predictions from the diversity of hypotheses, thereby sharpening their tests [28, 29]. Thus, here we formulate an evolutionary model that can be used to ask what quantitative and qualitative predictions arise from the various hypotheses for complex-cognition or large-brain evolution once these hypotheses are expressed in mathematical form.

A successful modeling approach in evolutionary research is life history theory [30–35]. Life history theory considers decisions regarding resouce allocation that individuals make over their lifespan, taking into account tradeoffs between competing ends (e.g., current vs future reproduction, number vs size of offspring, and growth vs reproduction) [30, 33, 34]. Such tradeoffs are of particular importance in complex-cognition and large-brain evolution because the brain uses copious amounts of energy that could be used for other functions [36–39]. While life history theory has been thoroughly used to explain the distribution of a variety of traits (e.g., [40–44]), it has remained relatively underdeveloped for cognition and brain (but see [29, 45, 46]). A first barrier is that mathematical modeling of brain evolution must describe how the brain impacts fitness without being overwhelmed by brain mechanistic details and at the same time it should consider enough mechanistic details to be able to make testable predictions. Existing models have described brain’s impact on fitness as facilitating energy acquisition from the environment allowing this energy to be used to increase survival [46], as facilitating energy production and/or decreasing the probability of being scrounged by others [47], as increasing offspring survival via parental care despite increasing mortality at birth [48], as increasing collaborative efficiency [49], as increasing mating ability [50], and as increasing the complexity of decision making regarding cooperation [51]. While these models have contributed to the understanding of brain evolution, a life history model is still lacking that incorporates real estimates of the metabolic costs of the brain while causally yielding quantitative predictions for brain and body size throughout ontogeny from a given brain-evolution hypothesis.

However, producing quantitative predictions that match empirical data from causal mathematical models is challenging given biological complexity. Despite this difficulty, metabolic theory has been successful at making quantitative predictions about ontogenetic body mass with the added bonus that its focus on metabolism allows using a top-down perspective without the need to describe the inner functioning of the system [52–56]. Thus, with the aim of producing quantitative predictions from given brain-evolution hypotheses, we combine elements of life history and metabolic theories to derive a mathematical model to study brain life history evolution. The model can be used to determine an individual’s optimal strategy regarding its energy allocation to the growth of its different tissues at each point in its life, which allows obtaining quantitative predictions for body and brain size under different biological settings.

Our model builds on previous approaches considering brain as embodied capital invested in fitness [46]. We consider separately the physical and functional embodied capital, the former being brain itself and the latter being skills (or cognitive abilities, knowledge, information, etc.) generated by the brain during ontogeny. Thus, our life-history approach considers skills throughout ontogeny. This implements the notion that information gained and maintained by the brain during growth should be considered when modeling brain evolution because selection for skill learning may delay maturity and thus impose tradeoffs among brain, body, and reproduction [1, 8, 45, 46, 57]. A defining feature of our model is that it assumes that some of the brain’s energetic consumption at each time is due to acquisition and maintenance of skills (or cognitive abilities, knowledge, information, etc.). In turn, we consider skills that allow overcoming energy-extraction challenges that the individual faces at each age, and by doing so the individual obtains some energetic reward. The model allows to study various biological settings depending on what poses the challenge faced at a given age and on who engages in overcoming the challenge: that is, the challenge can be posed by the non-social environment (nature), or it can be posed by social partners (them); also, the individual can engage in overcoming the challenge alone (me) or in concert with social partners (us): me (or us) vs nature (or them) [4, 5, 8, 45, 46, 49]. By applying different settings, our framework allows to investigate different brain-evolution hypotheses.

We apply our model to a baseline setting where individuals face exclusively ecological (non-social) challenges which are overcome by the individual alone (“me vs nature” [49]). This application captures basic aspects of hypotheses emphasizing ecological challenges as drivers of large-brain and complex-cognition evolution (e.g., that the non-social environment is a primary driver). Then, given that the brain consumes some of its energy to gain and maintain skills and given the various types of challenges that the individual faces at each age, we obtain a model that allows to predict how much an individual should grow its brain to obtain the energetic returns from skills. By feeding the model with parameter values for modern humans, we show how the model can yield predictions for life history stages as well as ontogenetic body and brain mass. We intend our me-vs-nature application to be compared with future applications of the model that include additional aspects of ecological-challenge hypotheses (e.g., variable environments [58]) and aspects of social-challenge hypotheses.

## Model

### Biological scenario

We consider a clonal, well-mixed, female population of large and constant size, where the environment is constant, generations are overlapping, and individuals’ age is measured in continuous time (i.e., standard demographic assumptions of models of life history evolution [32, 35, 40, 42, 59, 60]). We partition the body of each female into three types of tissues (or cells): reproductive tissue, brain tissue, and the remainder tissue, which we refer to as somatic. To have energy at each age for body growth, body maintenance, and reproduction, each female extracts energy from its environment (e.g., by locating food, or by making resources usable through cracking or cooking), possibly with the help of its mother (maternal care) and/or by interacting with other individuals in the population (e.g., through cooperative hunting or social competition for resources). To extract energy, each individual is assumed to use a number of relevant energy-extraction skills, which are produced and maintained by the brain.

We aim to determine the optimal allocation strategy of an individual’s energy budget to the growth of the different tissues throughout its lifespan, which simultaneously addresses the central life history question that asks what the optimal allocation to reproduction is ([61] p. 43; [33] p. 72; [62] p. 109; [32, 35, 40, 42, 59, 60]). An allocation strategy is here a vector of evolving traits that is a function of the individual’s age, and that determines the individual’s energy allocation to the growth of its different tissues throughout the individual’s lifespan. To analyze how selection affects the evolution of the allocation strategy, we carry out an evolutionary invasion analysis (e.g., [35, 63–65]), and thus consider that only two strategies can occur in the population, a mutant u and a resident (wild-type) v allocation strategies. As is standard in evolutionary invasion analysis, we thus seek to establish which strategy is uninvadable, that is, resistant to invasion by any alternative strategy taken from the set U of feasible allocation strategies, and which thus provides a likely final point of evolution [66–68]. From demographic assumptions we make below, it is well established [59, 68–72] that an uninvadable strategy $u^{*}$ satisfies

$$u^{*}\in \arg\max_{u\in U}R_{0}\left(u,u^{*}\right),$$ (1)

which implies that $u^{*}$ is a best response to itself, where

$$R_{0}\left(u,v\right)=\int_{0}^{T}l\left(t\right)m\left(t\right)dt$$ (2)

is the basic reproductive number (lifetime number of offspring) of a single mutant in an otherwise monomorphic resident population, and *T* is an age after which the individual no longer reproduces or is dead. The basic reproductive number depends on the probability *l*(*t*) that a mutant individual survives from birth until age *t* and on its rate *m*(*t*) of offspring production at age *t* with density dependence (“effective fecundity” [73], or the expected number of offspring produced at age *t* per unit time with density dependence), where these two vital rates may be functions of mutant and resident traits, u and v.

To determine the lifetime offspring production *R*_0_ and how it connects to the state variables (tissues and skill) and to the evolving traits, we relate brain and skill growth to vital rates, which in turn is mediated by the connection between energy extraction, metabolism, and tissue growth. We thus formally derive our model by making these connections.

### Tracking resting metabolic rate

Standard life history models refer to complete components of the energy budget (e.g., assimilated energy [74]). In practice, it is easier to measure heat release (metabolic rates [75]). Hence, to facilitate empirical parameter estimation, we follow the approach of [54] and formulate our life history model in terms of resting metabolic rate allocation, rather than energy budget allocation. Thus, we track how resting metabolic rate is due to growth and maintenance of different tissues, in particular the brain.

**Fig 1:**
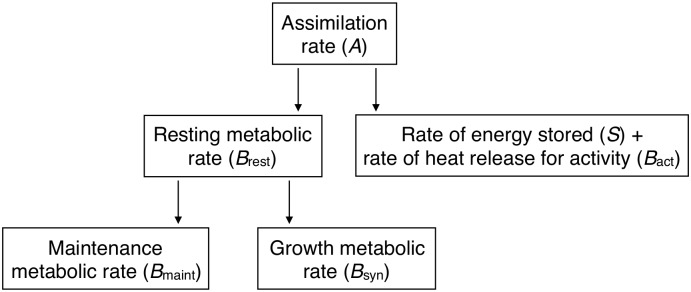
Partition of energy budget. Note the relation of resting metabolic rate to assimilation rate. Modified from [55].

We start from the partition of the individual’s energy budget used by [55], which divides the energy budget (assimilation rate) into heat released at rest (resting metabolic rate) and the remainder (Fig 1; see [75] for details justifying this partition). The amount of energy used per unit time by an individual is its assimilation rate. Part of this energy per unit time is stored in the body (*S*) and the rest is the total metabolic rate, which is the energy released as heat per unit time after use. Part of the total metabolic rate is the resting metabolic rate *B*_rest_ and the remainder is the energy released as heat per unit time due to activity *B*_act_. In turn, part of the resting metabolic rate is due to maintenance of existing biomass *B*_maint_, and the remainder is due to production of new biomass *B*_syn_. We refer to *B*_syn_ as the growth metabolic rate (Fig 1). We formulate our model in terms of allocation of growth metabolic rate *B*_syn_ to the growth of the different tissues.

### Energy partitioning

Denote by *N*_*i*_(*t*) the number of cells of type *i* of a focal mutant female of age *t*, where *i* ∈ {b, r, s} corresponds to brain, reproductive, and the remainder cells which we refer to as somatic, respectively. Assume that an average cell of type *i* in the resting body releases an amount of heat *B*_c*i*_ per unit time. Hence, the total amount of heat released per unit time by existing cells in the resting individual is

$$B_{\text{maint}}\left(t\right)=N_{b}\left(t\right)B_{cb}+N_{r}\left(t\right)B_{cr}+N_{s}\left(t\right)B_{cs},$$ (3)

which gives the part of resting metabolic rate due to body mass maintenance [55].

Denote by ${\dot{N}}_{i}\left(t\right)$ the time derivative of *N*_*i*_(*t*). Assume that producing a new average cell of type *i* releases an amount of heat *E*_c*i*_. Hence, the total amount of heat released per unit time by the resting individual due to production of new cells is

$$B_{\text{syn}}\left(t\right)={\dot{N}}_{b}\left(t\right)E_{cb}+{\dot{N}}_{r}\left(t\right)E_{cr}+{\dot{N}}_{s}\left(t\right)E_{cs},$$ (4)

which gives the rate of heat release in biosynthesis [55], and we call it the growth metabolic rate. From Eq (4), we have that

$${\dot{N}}_{i}\left(t\right)E_{ci}=u_{i}\left(t\right)B_{\text{syn}}\left(t\right),$$ (5)

for *i* ∈ {b, r, s}, where *u*_*i*_(*t*) is the fraction of the growth metabolic rate due to production of new type-*i* cells at time *t* (summing over all cell types in Eq 5 returns Eq 4). The resulting time sequence $u={\left\{u\left(t\right)\right\}}_{t=0}^{T}\in U$, where **u**(*t*) = (*u*_b_(*t*), *u*_r_(*t*), *u*_s_(*t*)), of allocations from birth to (reproductive) death is the evolving multidimensional trait in our model and U is the set of all feasible allocations strategies.

From our partitioning in Fig 1, the total amount of heat released by the resting individual per unit time at age *t* is

$$B_{\text{rest}}\left(t\right)=B_{\text{maint}}\left(t\right)+B_{\text{syn}}\left(t\right),$$ (6)

which is the individual’s resting metabolic rate at age *t*.

### Tissue growth rate

Let the mass of an average cell of type *i* be *x*_c*i*_ for *i* ∈ {b, r, s}. Then, changing units from cell number to mass, the mass of tissue *i* at age *t* is

$$x_{i}\left(t\right)=x_{ci}N_{i}\left(t\right),$$ (7)

and hence, using Eq (5), we have that the growth rate in mass of tissue *i* is

$$\begin{array}{ccc} {\dot{x}}_{i}\left(t\right) & = & x_{ci}{\dot{N}}_{i}\left(t\right)\\ & = & \frac{x_{ci}}{E_{ci}}u_{i}\left(t\right)B_{\text{syn}}\left(t\right). \end{array}$$ (8)

To continue the change of cell-number units to mass, we denote the heat released for producing an average mass unit of tissue *i* as *E*_*i*_ = *E*_c*i*_/*x*_c*i*_, which we assume constant with respect to time for simplicity. This gives

$${\dot{x}}_{i}\left(t\right)=u_{i}\left(t\right)\frac{B_{\text{syn}}\left(t\right)}{E_{i}}.$$ (9)

Substituting Eq (6) in Eq (9), we obtain the model’s first key equation specifying the growth rate of tissue *i*:

$${\dot{x}}_{i}\left(t\right)=u_{i}\left(t\right)\;\left(\frac{B_{\text{rest}}\left(t\right)-B_{\text{maint}}\left(t\right)}{E_{i}}\right),$$ (10)

where from Eqs (3) and (7), we have that

$$B_{\text{maint}}\left(t\right)=x_{b}\left(t\right)B_{b}+x_{r}\left(t\right)B_{r}+x_{s}\left(t\right)B_{s}$$ (11)

and the mass-specific cost of tissue maintenance is *B*_*i*_ = *B*_c*i*_/*x*_c*i*_, which we also assume constant with respect to time for simplicity. From Eqs (10) and (11), the mass unit in which *x*_*i*_(*t*) for *i* ∈ {b, r, s} is measured (e.g., gram or kilogram) is determined by the mass unit in *B*_*i*_ and *E*_*i*_.

### Skill learning rate

We assume that the individual at age *t* has a skill level *x*_k_(*t*) at energy extraction, which is a quantitative variable measuring the individual’s ability to overcome challenges of energy extraction. Skill level *x*_k_(*t*) can be measured on a scale that is best suited for each application, for example as the individual’s number of skills at age *t* (e.g., as may be useful in anthropology; [81]), or as an index of performance at a series of tasks (e.g., as in comparative psychology studies; [1]). We assume that some of the brain metabolic rate is due to acquiring and maintaining energy-extraction skills [76–80]. Denote by *B*_rest,b_(*t*) the brain metabolic rate of the individual at age *t* (i.e., the heat released by the brain per unit time with the individual at rest). From energy conservation, the brain metabolic rate must equal the heat released by the brain per unit time due to brain growth and brain maintenance; that is, from Eqs (3) and (4) in mass units, the brain metabolic rate must satisfy

$$B_{\text{rest},b}\left(t\right)=x_{b}\left(t\right)B_{b}+{\dot{x}}_{b}\left(t\right)E_{b}.$$ (12)

Let *s*_k_ be the fraction of brain metabolic rate allocated to energy-extraction skills, which we assume constant for simplicity. Suppose that the brain releases on average an amount of heat *E*_k_ for increasing skill level by one unit (learning cost; [76–78]). Similarly, assume that the brain releases on average an amount of heat *B*_k_ per unit time for maintaining a skill unit (memory cost; [79, 80]). For simplicity, we assume *E*_k_ and *B*_k_ to be constant with respect to time. From energy conservation, the rate of heat release by the brain due to skill growth and skill maintenance must equal the brain metabolic rate due to energy-extraction skill:

$$x_{k}\left(t\right)B_{k}+{\dot{x}}_{k}\left(t\right)E_{k}=s_{k}B_{\text{rest},b}\left(t\right).$$ (13)

Rearranging, we obtain the model’s second key equation specifying skill learning rate:

$${\dot{x}}_{k}\left(t\right)=\frac{s_{k}B_{\text{rest},b}\left(t\right)-x_{k}\left(t\right)B_{k}}{E_{k}}.$$ (14)

In analogy with Eq (10), the first term in the numerator of Eq (14) gives the heat released due to skill learning and memory whereas the second term gives the heat released for memory. [Note that an equation for skill growth rate can be similarly derived, not in terms of allocation to skill growth *and* maintenance *s*_k_, but in terms of allocation to skill growth *u*_*k*_ as for Eq (10).] As with Eq (10), from Eq (14) the unit in which *x*_k_(*t*) is measured is determined by the skill unit in *B*_k_ and *E*_k_.

### How skill affects energy extraction

We now derive an expression that specifies how brain affects energy extraction in the model. We consider that energy extraction depends on the focal female’s skills but possibly also on the skills of other females in the population. To make this dependence explicit, we denote by E(t,u,v) the amount of energy extracted by the focal female per unit time at time *t* from the environment. So, using Fig 1 the energy-extraction rate is

$$E\;\left(t,u,v\right)=A\left(t\right)+\text{surplus}.$$ (15)

E(t,u,v) depends on the mutant’s skill and possibly other features (control or state variables, or body mass) which ultimately depend on the mutant allocation strategy u. Additionally, the energy-extraction rate E(t,u,v) also depends on the skill or other features of the resident population which ultimately depend on the resident allocation strategy v. Let *E*_max_(*t*) be the amount of energy that the individual obtains from the environment per unit time at age *t* if it is maximally successful at energy extraction (which also possibly depends on body mass). We define the energy-extraction efficiency e(t,u,v) at age *t* as the normalized energy-extraction rate at age *t*:

$$e\;\left(t,u,v\right)=\frac{E\;\left(t,u,v\right)}{E_{\text{max}}\left(t\right)},$$ (16)

which is thus a dimensionless energy extraction performance measure.

We also define the ratio of resting metabolic rate to energy-extraction rate as

$$q\;\left(t,u,v\right)=\frac{B_{\text{rest}}\left(t\right)}{E\;\left(t,u,v\right)}$$ (17)

and, motivated by Eq (17), we define

$$B_{\text{rest},\text{max}}\left(t,u,v\right)=q\;\left(t,u,v\right)E_{\text{max}}\left(t\right)$$ (18a)

$$=\frac{B_{\text{rest}}\left(t\right)}{e\;\left(t,u,v\right)}.$$ (18b)

From Eq (18b), we have that

$$B_{\text{rest}}\left(t\right)=e\;\left(t,u,v\right)B_{\text{rest},\text{max}}\left(t,u,v\right).$$ (19)

Consequently, $B_{\text{rest},\text{max}}\left(t,u,v\right)$ gives the resting metabolic rate when the individual is maximally successful at energy extraction.

Adult resting metabolic rate typically scales with adult body mass as a power law across all living systems [82–85], and also ontogenetically in humans to a good approximation (Fig C in S1 Appendix; but see [86]). Such scaling is empirically obtained from measurements in mostly well-fed individuals, and thus we assume that the empirically measured power-law scaling applies to the resting metabolic rate when the individual is maximally successful at energy extraction, $B_{\text{rest},\text{max}}\left(t,u,v\right)$; that is, we assume

$$B_{\text{rest},\text{max}}\left(t,u,v\right)=Kx_{B}^{\beta }\left(t\right),$$ (20)

where *x*_B_(*t*) = *x*_b_(*t*) + *x*_r_(*t*) + *x*_s_(*t*) is body mass at age *t*, *β* is a scaling coefficient, and *K* is a constant independent of body mass (both *β* and *K* may depend on the resident strategy; note that *β* need not be 3/4). We further assume that energy-extraction efficiency e(t,u,v) is independent of body mass, whereby Eqs (19) and (20) yield the model’s third key equation specifying resting metabolic rate as:

$$B_{\text{rest}}\left(t\right)=Ke\;\left(t,u,v\right)\;x_{B}^{\beta }\left(t\right).$$ (21)

Eq (21) has two noteworthy implications. First, energy-extraction efficiency e(t,u,v) regulates the amount of heat that is released from tissue maintenance and synthesis: e.g., a zero energy-extraction efficiency means no heat is released at rest. Second, Eq (21) implies that an increase in body mass is accompanied by an increase in the rate of heat release *B*_rest_(*t*). But from Eq (15) and Fig 1 we have that *E*(*t*) = *B*_rest_(*t*) + *S*(*t*) + *B*_act_(*t*) + surplus, which means that an increase in the rate of heat release *B*_rest_(*t*) due to increased body mass must be accompanied by either (1) an increase in the energy-extraction rate *E*(*t*), (2) a decrease in the rate of energy stored *S*(*t*), (3) a decrease in the rate of heat release due to activity *B*_act_(*t*), or (4) a decrease in the surplus:

$$\frac{\partial B_{\text{rest}}\left(t\right)}{\partial x_{B}}=\frac{\partial E(t)}{\partial x_{B}}-\frac{\partial S(t)}{\partial x_{B}}-\frac{\partial B_{\text{act}}\left(t\right)}{\partial x_{B}}-\frac{\partial \;\text{surplus}}{\partial x_{B}}.$$ (22)

That is, Eq (21) specifies a physical constraint imposed by body size that requires that an increased resting metabolic rate due to a larger body mass is achieved by balancing the energy budget from the mentioned sources so that Eq (22) is satisfied. Given that a power law between resting metabolic rate and body mass is ubiquitously observed across living systems [82–85], we assume that individuals adjust their energy budget as just described (i.e., satisfy Eq 22) so that the physical constraint implied by Eq (21) is met.

Consequently, Eq (21) implies that increasing a tissue’s mass increases the resting metabolic rate which may increase the growth metabolic rate. To see this, consider the following. From Eqs (6), (21) and (11), the increase in growth metabolic rate with an increase in the mass of tissue *i* ∈ {b, r, s} is

$$\frac{\partial B_{\text{rest}}}{\partial x_{i}}-\frac{\partial B_{\text{maint}}}{\partial x_{i}}=Ke\;\left(t,u,v\right)\;\beta x_{B}^{\beta -1}\left(t\right)-B_{i}.$$ (23)

When Eq (23) is positive, an increase in the mass of tissue *i* increases the growth metabolic rate: resting metabolic rate increases more than maintenance metabolic rate, so there is a remainder due to growth. In addition, this remainder is greatest if *i* is the cheapest tissue to maintain (i.e., if *B*_*i*_ is the smallest for *i* ∈ {b, r, s}). Therefore, if a tissue (e.g., brain) is favored to grow, other particularly cheap-to-maintain tissues (e.g., soma) may be favored to grow if they increase the growth metabolic rate, which reflects an increase in the energy available for growth given that the individual balances its energy budget as indicated by Eq (22).

### Closing the model: Assume a hypothesis to obtain a closed model capable of predictions

The expression for the resting metabolic rate (Eq 21) closes the model from a metabolic point of view, since after substituting Eq (21) in Eq (10) (and using Eqs 11, 12, 14), the ontogenetic dynamics of the brain, reproductive, and somatic tissue mass, *x*_b_(*t*), *x*_r_(*t*), and *x*_s_(*t*), and of skill level, *x*_k_(*t*), are expressed in terms of such state variables, of empirically estimable parameters, and on the evolving traits (mutant u and resident v). That is, the ontogenetic dynamics are given by

$$\begin{array}{cc} {\dot{x}}_{i}\left(t\right)=u_{i}\left(t\right)\frac{1}{E_{i}} & [Ke\text{​}\left(t,u,v\right)\;\;x_{\text{B}}^{\beta }\left(t\right)\\ & -x_{\text{b}}\left(t\right)B_{\text{b}}-x_{\text{r}}\left(t\right)B_{\text{r}}-x_{\text{s}}\left(t\right)B_{\text{s}}]\;\text{for}\;i\in \left\{\text{b},\text{r},\text{s}\right\} \end{array}$$ (24a)

$$\begin{array}{cc} {\dot{x}}_{\text{k}}\left(t\right)=\frac{1}{E_{\text{k}}} & \{s_{\text{k}}[x_{\text{b}}\left(t\right)B_{\text{b}}\\ & \;+u_{\text{b}}\left(t\right)\;\left(Ke\text{​}\left(t,u,v\right)\;\;x_{\text{B}}^{\beta }\left(t\right)-x_{\text{b}}\left(t\right)B_{\text{b}}-x_{\text{r}}\left(t\right)B_{\text{r}}-x_{\text{s}}\left(t\right)B_{\text{s}}\right)]\\ & -x_{\text{k}}\left(t\right)B_{\text{k}}\}. \end{array}$$ (24b)

The ontogenetic dynamics of the state variables *x*_*i*_(*t*) are thus non-linear.

To close the model from an evolutionary perspective and compute an optimal allocation strategy, we need expressions for how the state variables relate to the vital rates [*l*(*t*) and *m*(*t*)] in Eq (2) and expressions for the energy-extraction efficiency [e(t,u,v)]. A large number of settings can be conceived with the model so far, both for the vital rates and energy-extraction efficiency. We focus on an application aiming at modeling human brain evolution from a baseline “me-vs-nature” setting.

#### Vital rates

For simplicity, we consider that the mortality rate *μ* is independent of age and of the evolving traits, so

l(t)=exp(-μt). (25)

We also assume that density-dependent regulation acts on fecundity (e.g., through lottery competition) so that the effective fecundity *m*(*t*) is proportional to fecundity *f*(*t*), defined as the rate of offspring production at age *t* without density dependence (e.g., [69, 73, 87]). That is, we let

m(t)=C(v)f(t) (26)

where C(v) is a proportionality factor that depends on population size which ultimately depends on the resident strategy v.

We obtain a measure of fecundity *f*(*t*) with a reasoning analogous to that used for the learning rate of skills. In particular, we assume that some of the metabolic rate of the reproductive tissue is due to offspring production and maintenance. Denote by *B*_rest,r_(*t*) the metabolic rate of the reproductive tissue at age *t* (i.e., the heat released by the reproductive tissue per unit time with the individual at rest). From energy conservation and Eqs (3) and (4) in mass units, the reproductive metabolic rate must satisfy

$$B_{\text{rest},r}\left(t\right)=x_{r}\left(t\right)B_{r}+{\dot{x}}_{r}\left(t\right)E_{r}.$$ (27)

Let *s*_o_ be the fraction of the reproductive metabolic rate allocated to offspring production and maintenance, which we assume constant for simplicity. Let *x*_o_(*t*) be the number of offspring the individual has at age *t*. Suppose that the reproductive tissue releases an amount of heat *E*_o_ for the production of an average offspring (fecundity cost). Similarly, assume that the reproductive tissue releases an amount of heat *B*_o_ per unit time for maintaining an average offspring (physiological cost of maternal care; e.g., due to lactation). Hence, from energy conservation, the rate of heat release by the reproductive tissue due to offspring production and maintenance must equal the reproductive metabolic rate allocated to offspring production and maintenance:

$$x_{\text{o}}\left(t\right)B_{\text{o}}+{\dot{x}}_{\text{o}}\left(t\right)E_{\text{o}}=s_{\text{o}}B_{\text{rest},r}\left(t\right).$$ (28)

Rearranging, we obtain the model’s fourth key equation specifying fecundity:

$$f\left(t\right)={\dot{x}}_{o}\left(t\right)=\frac{s_{\text{o}}B_{\text{rest},r}\left(t\right)-x_{\text{o}}\left(t\right)B_{\text{o}}}{E_{\text{o}}}.$$ (29)

The first term in the numerator of Eq (29) gives the heat released due to reproduction and physiological maternal care whereas the second term gives the heat released for physiological maternal care.

We simplify Eq (29) as follows. If the reproductive tissue is defined narrowly enough (e.g., as preovulatory ovarian follicles) so that it is not involved in offspring maintenance, the physiological costs of maternal care incurred by the reproductive tissue are essentially null (i.e., *B*_o_ ≈ 0; they are, however, included in the maintenance costs *B*_s_ of the somatic tissue as we defined it above). If additionally, reproductive tissue maintenance is much more expensive than production (i.e., *B*_r_ ≫ *E*_r_/(time unit), which holds with our estimated parameters for humans; Table B in S1 Appendix), using Eq (27) in Eq (29), fecundity can be approximated as

$$f\left(t\right)\approx \frac{s_{\text{o}}B_{r}}{E_{\text{o}}}x_{r}\left(t\right).$$ (30)

For the results reported below, the approximation Eq (30) is accurate and yields no detectable difference in the predicted adult brain and body mass [section 8.2.1 in S1 Appendix].

Using Eqs (26) and (30), effective fecundity becomes

$$m\left(t\right)\approx C\left(v\right)f_{0}x_{r}\left(t\right),$$ (31)

where *f*_0_ = *s*_o_*B*_r_/*E*_o_ is the number of offspring produced per unit time per mass unit of reproductive tissue in the absence of density dependence. We assume that non-physiological costs of maternal care are included in $C\left(v\right)f_{0}$. Effective fecundity is then proportional to the mass of reproductive tissue, which is consistent with medical approaches that predict women’s fecundity in terms of ovarian follicle count [88, 89].

#### Energy acquisition

We now model energy acquisition by specifying expressions for energy-extraction efficiency e(t,u,v). Considering different expressions for e(t,u,v) allows incorporating key aspects of hypotheses for cognition and brain evolution. For instance, hypotheses postulating ecological challenges (e.g., food gathering or food processing) as drivers of cognition and brain evolution can be modeled by letting the energy-extraction efficiency e(t,u,v) depend on the individual’s skills and the non-social environment. Alternatively, hypotheses postulating social challenges involving cooperation (e.g., hunting big game) can be modeled by letting e(t,u,v) have a positive dependence on the skills of both the individual and those of its social partners. Similarly, hypotheses postulating social challenges involving competition (e.g., outsmarting social partners) can be modeled by letting e(t,u,v) have a negative dependence on the skills of the social partners. Ideally, expressions for e(t,u,v) would be empirically informed. In the cases just mentioned, identifying uninvadable allocation strategies for non-social models involves an optimal control problem, while for the social models it involves a differential game problem. As a first approximation, we address here the non-social situation using plausible expressions for e(t,u,v) that are at present not empirically grounded but that are based on expressions previously derived from basic assumptions (axioms) about contests [90, 91].

We assume that energy extraction at age *t* is done exclusively by overcoming a challenge posed by the non-social environment (e.g., gathering food or lighting a fire) and that the individual engages alone but possibly with its mother’s help in overcoming such a challenge (“me vs nature”). From our assumption of clonality, the mother of a mutant is equally mutant. Thus, this me-vs-nature setting implies that the energy-extraction efficiency, e(t,u,v)=e(t,u) is independent of the resident strategy.

We treat the me-vs-nature setting as a contest against the environment. We thus let energy-extraction efficiency e(t,u) take the form of a contest success function [90, 92]:

$$e\left(t,u\right)=\frac{c(x_{k}\left(t\right))}{d\left(t\right)+c(x_{k}\left(t\right))},$$ (32)

which depends on two terms. First, energy-extraction efficiency depends on the difficulty of the challenge at age *t*, measured by *d*(*t*). The higher *d*(*t*), the more challenging energy extraction is and the higher the energy-extraction skill level must be to obtain resources. We let *d*(*t*) = *α*[1 − *φ*(*t*)], where *α* is the environmental difficulty and *φ*(*t*) is the facilitation of the challenge due to maternal care. For simplicity, we let this facilitation be independent of maternal skill (exogenously determined facilitation; see section 8.2.3 in S1 Appendix and section “Skill through ontogeny” below for a relaxation of this assumption that makes maternal facilitation *φ*(*t*) endogenous, i.e., depend on maternal skill). Thus, we let maternal facilitation be an exponentially decreasing function of age:

$$\varphi \left(t\right)=\varphi_{0}\exp\left(-\varphi_{r}t\right)$$ (33)

where *φ*_0_ is the maternal facilitation at birth and *φ*_r_ is its rate of decrease. For simplicity, we ignore the increased resting metabolic rate caused by gestation and lactation [93].

Second, energy-extraction efficiency depends on the individual’s competence, denoted by *c*(*x*_k_(*t*)). We consider two cases that are standard in contest models: (1) a power function *c*(*x*_k_(*t*)) = [*x*_k_(*t*)]^*γ*^, so energy-extraction efficiency *e*(*x*_k_(*t*)) is a contest success function in ratio form (power competence); and (2) an exponential function *c*(*x*_k_(*t*)) = [exp(*x*_k_(*t*))]^*γ*^ so energy-extraction efficiency is in difference form (exponential competence) [90, 92]. In both cases, the parameter *γ* describes the effectiveness of skills at energy extraction. Thus, with *γ* = 0, skills are ineffective while with increasing *γ* a lower skill level is sufficient to extract energy. In general, competence *c*(*x*_k_(*t*)) represents features of the individual (e.g., how increasing skill changes efficiency in information processing by the brain), and of the environment (e.g., how adding the skill of caching nuts to that of cracking nuts changes energy-extraction efficiency). For a given skill effectiveness (*γ*), exponential competence assumes a steeper increase in competence with increasing skill than power competence.

#### Model summary and solution implementation

**Fig 2:**
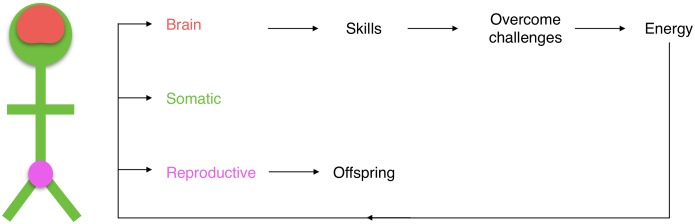
Schematic description of the model. The body is partitioned in three tissues. Some of the energetic expense of the reproductive tissue is for producing and maintaining offspring, and some of the energetic expense of the brain is for producing and maintaining energy-extraction skills. These skills allow extracting energy that can be used to produce and maintain the different tissues.

On substituting Eqs (25) and (31) into Eq (2) along with Eq (32) into Eq (24), the model is closed and can be used to determine uninvadable allocation strategies and the resulting equilibrium growth patterns. A schematic description of the model is in Fig 2. From our simplifying assumptions, determining an uninvadable allocation strategy reduces to an optimal control problem. We obtain locally uninvadable allocation strategies using optimal control methodology (e.g., [94]), both by a “direct simultaneous” approach with the software GPOPS [95] for numerical approximations and by an “indirect” approach using Pontryagin’s Maximum Principle for analytical results (sections 1–4 in S1 Appendix). GPOPS is based on a pseudospectral method which converts the optimal control problem into a nonlinear program that can be addressed using available solvers. This direct solution approach partitions the time interval [0, *T*] into sub intervals and, using an initial guess of the (allocation) solution (control), finds the resulting value of the (lifetime number of offspring) objective. Applying the gradient of the objective with respect to a representation of the control, this approach refines the control until no higher objective is attained. In contrast, the indirect approach uses necessary first-order conditions that the solution must satisfy to construct the solution (see section 7 in S1 Appendix for additional method details).

The model depends on 22 parameters which measure (P1) tissue mass in the newborn (*x*_*i*0_ for *i* ∈ {b, r, s}), (P2) tissue metabolism (*K*, *β*, *B*_*i*_ and *E*_*i*_ for *i* ∈ {b, r, s}), (P3) demography (*f*_0_, *μ*, and *T*), (P4) skill of the newborn (*x*_k0_), (P5) skill metabolism (*s*_k_, *B*_k_ and *E*_k_), (P6) maternal care (*φ*_0_ and *φ*_r_), and (P7) energy extraction (*α* and *γ*). From their definitions, the parameters are measured in units of mass, energy, time, and skill. The parameter *f*_0_ only displaces the objective vertically and thus has no effect on the uninvadable allocation strategies. The parameter *T* is taken finite for numerical implementation and set as the observed age of menopause.

We use published data for human females to estimate 13 parameters that affect the uninvadable allocation strategies (P1–P3) (section 5,6 and Table B in S1 Appendix). These parameters include the mass-specific production and maintenance metabolic costs of the different tissues including the brain (*B*_*i*_ and *E*_*i*_ for *i* ∈ {b, r, s}), and with these parameters and in particular with their relative values fixed, the model can only generate a vastly narrower set of outcomes. These 13 parameters are in units of mass, energy, and time which we measure in kg, MJ (megajoules), and years, respectively. The remaining 8 parameters that affect the uninvadable allocation strategies (P4–P7) measure skill metabolism, skill effectiveness, environmental difficulty, and maternal care, and given that an empirically grounded relationship between skill and energy-extraction efficiency is still unavailable, these parameters are less easily estimated from available data. Their values could be estimated using optimization algorithms that minimize the difference between predicted and observed body and brain mass, but given the complexity of the model, such investigations are beyond the scope of the present paper. As a first approximation, for these parameters we identify by trial and error benchmark values that yield a model output roughly in agreement with observed ontogenetic body and brain mass data for modern human females. Such benchmark parameter values are different with power (Table C in S1 Appendix) and exponential (Table D in S1 Appendix) competence. The benchmark parameter values include *B*_k_ and *E*_k_ which determine skill level units, and as we do not estimate these parameters from empirical data, we measure skill level in unspecified units denoted as “skill unit”. We first present the numerical results for the two sets of benchmark parameter values and then the results when deviating from them (see S1 Appendix for analytical results and S1 Computer Code for numerical implementation).

## Results

### Predicted life history stages: Childhood, adolescence, and adulthood

**Fig 3:**
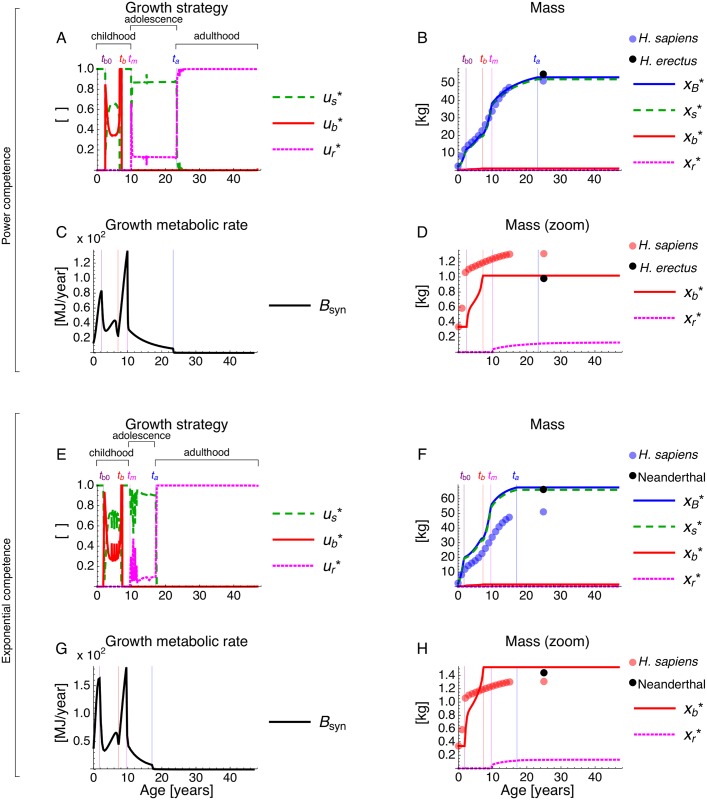
Locally uninvadable growth strategy ($u_{i}^{*}$) and the resulting growth patterns ($x_{i}^{*}$) under a me-vs-nature setting. Lines are model’s results and circles are observed values in human females. Results with (A-D) power and (E-H) exponential competence. (A,E) Uninvadable growth strategy vs age. (C,G) Resulting growth metabolic rate vs age. (B,F) Resulting body and tissue mass vs age. (D,H) Resulting brain and reproductive mass vs age. Lines and circles with the same color are respectively the model’s prediction and the observed values in modern human females [96]. Black circles are the observed (B,F) adult female body mass and (D,H) adult sex-averaged brain mass, either for late *H. erectus* [97] or Neanderthals [98, 99]. The growth strategy (A,E) has numerical jitter due to negligible numerical error (Fig B in S1 Appendix) and is here filtered for figure clarity (see unfiltered figure in Fig D in S1 Appendix).

The optimal strategy we obtain divides the individual’s lifespan in three broad stages: (1) a “childhood” stage, defined as the stage lasting from birth to the age at maturity *t*_m_, during which allocation to growth of reproductive tissue is zero; (2) an “adolescence” stage, defined as the stage lasting from *t*_m_ to the age at adulthood *t*_a_, during which there is simultaneous allocation to growth of somatic and reproductive tissue; and (3) an “adulthood” stage, defined as the stage lasting from *t*_a_ to the end of the individual’s reproductive career, during which all growth allocation is to reproductive tissue (Fig 3A). These life stages are obtained with either power or exponential competence (Fig 3A and 3E). Note that the ages at maturity *t*_m_ and adulthood *t*_a_ (switching times) are not parameters but an output of the model.

The obtained childhood stage, which is the only stage where there is allocation to brain growth, is further subdivided in three periods: (1a) “ante childhood”, defined here as lasting from birth to the age of brain growth onset *t*_b0_, during which there is pure allocation to somatic growth; (1b) “childhood proper”, defined here as lasting from *t*_b0_ to the age of brain growth arrest *t*_b_, during which there is simultaneous allocation to somatic and brain growth; and (1c) “preadolescence”, defined here as lasting from *t*_b_ to *t*_m_, during which there is pure somatic growth. Hence, brain growth occurs exclusively during “childhood proper”. The occurrence of an “ante childhood” without brain growth disagrees with observation in humans. Two possible and particularly relevant reasons for this discrepancy may be either the absence of social interactions in this setting of the model, or the approximation of resting metabolic rate by a power law (Eq 21) which underestimates resting metabolic rate (and thus growth metabolic rate) during ante childhood (Fig C in S1 Appendix; note that although improving the fit of resting metabolic rate with body mass is straightforward, this introduces additional non-linearities that make the optimal control problem numerically more challenging and this exploration is beyond the scope of the present paper). The switching times *t*_b0_ and *t*_b_ are also an output rather than parameters of the model (Fig 3A).

With the exception of the age of brain growth onset, the predicted timing of childhood, adolescence, and adulthood closely follows that observed in humans with competence being either a power or an exponential function of skill level, given their respective benchmark parameter values (Table 1). Recall that measurement units (i.e., years, kg, and MJ), excepting skill units, are real in that they result from the units of the parameter values estimated from empirical data (Table B in S1 Appendix). Hence, while using realistic metabolic costs of brain and body, the model can correctly predict major stages of human life history with accurate timing, with the exception of no brain growth allocation during ante childhood (Table 1). Later in the paper we address how these results change with changes in parameter values.

**Table 1:** Predictions for life history timing and adult brain and body mass with a me-vs-nature setting and the benchmark parameter values. Switching times and adult values resulting with competence as a power or exponential function (PC and EC) for the results in Fig 3. *Observed values in human females: age at maturity [100], adulthood [101], brain growth onset and arrest [96], adult body mass [96], and adult brain mass [96]. †Encephalization quotient, calculated as $\text{EQ}=x_{b}^{*}\left(t_{a}\right)/[11.22\times 10^{-3}x_{\text{B}}^{*}{\left(t_{a}\right)}^{0.76}]$ (mass in kg) [102].

		Predicted with		Observed in*
		PC	EC	*H. sapiens*
Age at:	Maturity, *t*_m_ [y]	9.94	9.70	7–13
	Adulthood, *t*_a_ [y]	23.37	17.33	≈17
	Brain growth onset, *t*_b0_ [y]	2.36	1.81	0
	Brain growth arrest, *t*_b_ [y]	7.19	7.34	≈17
	Adult body mass, [kg]	53.19	67.79	51.1
	Adult brain mass, [kg]	1.02	1.53	1.31
	EQ^†^, [ ]	4.43	5.52	5.87

### Body and brain mass through ontogeny

The optimal growth strategy generates the following predicted body and brain mass throughout ontogeny. For total body mass, there is fast growth during ante childhood, followed by slow growth during childhood proper, a growth spurt during preadolescence, slow growth during adolescence, and no growth during adulthood, each of which closely follows the observed growth pattern in humans (Fig 3B). Most body growth is due to somatic growth, and this results because if a tissue mass is favored to grow (e.g., brain) the energy required for its production can be made available by increasing the growth metabolic rate by producing the cheapest tissue to maintain which is the soma (Eq 23; Table B in S1 Appendix), under our assumption that individuals balance their energy budget as in Eq (22). The slow body growth during childhood proper results from the simultaneous allocation to somatic and brain growth and from the decreasing growth metabolic rate due to the increasing energetic costs of brain maintenance (Fig 3C). The growth spurt during preadolescence arises because (1) all growth metabolic rate is allocated to inexpensive somatic growth, and (2) growth metabolic rate increases due to increased metabolic rate caused by increasing, inexpensive-to-maintain somatic mass (Fig 3C). The slow growth during adolescence is due to simultaneous somatic and reproductive growth, and to the elevated costs of reproductive tissue maintenance (Fig 3C). These growth patterns result in two major peaks in growth metabolic rate (Fig 3C). While the first peak in growth metabolic rate is made possible by maternal care, the second peak is made possible by the individual’s own skills (Fig G panel D in S1 Appendix). After the onset of adulthood at *t*_a_, growth metabolic rate is virtually depleted and allocation to growth has essentially no effect on tissue growth (Fig 3C).

Whereas predicted body growth patterns are qualitatively similar with either power or exponential competence, they differ quantitatively (Fig 3B and 3F). With power competence, the predicted body mass is nearly identical to that observed in human females throughout life (Fig 3B). In contrast, with exponential competence, the predicted body mass is larger throughout life than that of human females (Fig 3F). Our exploration of the parameter space indicates that the larger body mass with exponential competence relative to power competence is robust to parameter change (see section “A large brain is favored…” below and section 8.8 in S1 Appendix).

Regarding brain mass, the model predicts it to have the following growth pattern. During ante childhood, brain mass remains static, in contrast to the observed pattern (Fig 3D). During childhood proper, brain mass initially grows quickly, then it slows down slightly, and finally grows quickly again before brain growth arrest at the onset of preadolescence (Fig 3D). Predicted brain growth is thus delayed by the obtained ante-childhood period relative to the observed brain growth in humans (Fig 3D). As previously stated, such brain growth delay may be a result of the absence of social interactions in this model setting, or an inaccuracy arising from the underestimation of resting metabolic rate during ante childhood by the power law of body mass.

Predicted brain growth patterns are also qualitatively similar but quantitatively different with power and exponential competence (Fig 3D and 3H). Adult brain mass is predicted to be larger with competence as an exponential rather than as a power function (Fig 3D and 3H). As for body mass, our exploration of the parameter space indicates that the larger brain mass with exponential competence is robust to parameter change (see section “A large brain is favored…” below and section 8.8 in S1 Appendix). Moreover, the encephalization quotient (EQ, which is the ratio of observed adult brain mass over expected adult brain mass for a given body mass) is also larger with exponential competence for the benchmark parameter values (Table 1). For illustration, with competence as a power function, the predicted adult body and brain mass approach those observed in late *H. erectus* (Fig 3B and 3D). In contrast, with competence as an exponential function, the predicted adult body and brain mass approach those of Neanderthals (Fig 3F and 3H). The larger EQ with exponential competence is also robust to parameter change (see section “Factors favoring a large EQ…” below and 8.8 in S1 Appendix).

### Skill through ontogeny

**Fig 4:**
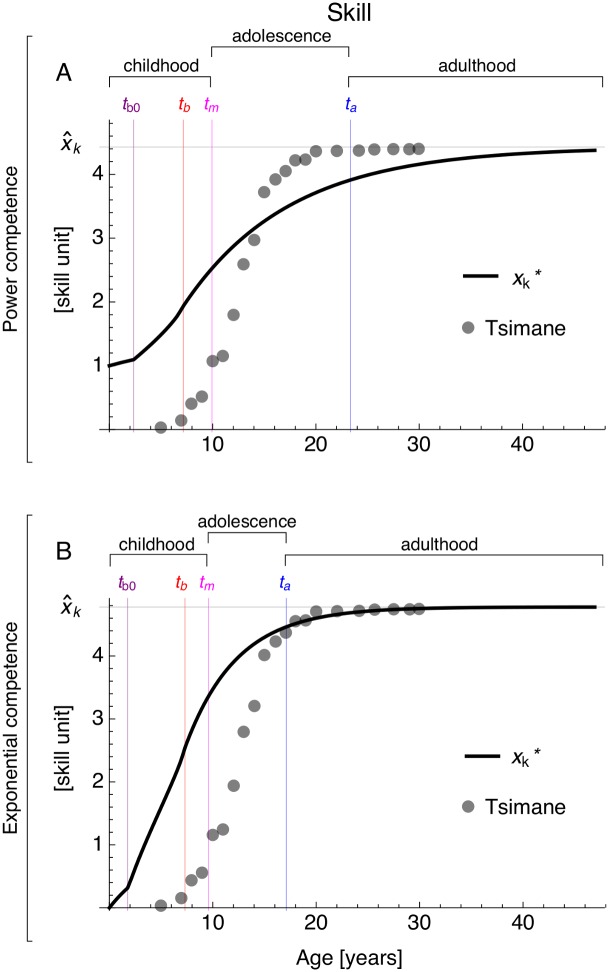
Predicted skill level at energy extraction throughout ontogeny plateaus in adulthood. Lines are the predicted skill level vs age with (A) power and (B) exponential competence for the results in Fig 3. Circles are the observed cumulative distribution of self-reported acquisition ages of food production skills in female Tsimane horticulturalists [81] multiplied by our ${\hat{x}}_{k}$. However, note that the observed skills in Tsimane involve cumulative social learning which we do not model explicitly.

The obtained optimal growth strategy predicts the following patterns for the skill level at energy-extraction throughout ontogeny. Under the same parameter values as in Fig 3, the individual’s skill level increases most during childhood and adolescence, skill level continues to increase after brain growth arrest, and skill level plateaus in adulthood (Fig 4). That is, skill growth is “determinate”, in agreement with empirical observations for food production skills (Fig 4). Yet, if memory cost *B*_k_ is substantially lower, skill level can continue to increase throughout life (i.e., skill growth is then “indeterminate”; Fig K panel E in S1 Appendix) (see Eq 14). Nevertheless, in that case, the agreement between predicted and observed body and brain mass throughout ontogeny is substantially reduced (Fig K panels B,C in S1 Appendix).

The requirement for skill growth to be determinate is that the brain metabolic rate allocated to skills [*s*_k_*B*_rest,b_(*t*)] becomes saturated with skill maintenance [*x*_k_(*t*)*B*_k_] within the individual’s life (Eq 14). Thus, skill can continue to grow after brain growth arrest if memory costs do not yet saturate the brain metabolic rate allocated to skills. When skill growth is determinate, an immediate prediction of the model is that adult skill level is proportional to adult brain mass. In particular, with determinate skill growth, the skill level that is asymptotically achieved [from Eq 24b setting ${\dot{x}}_{k}^{*}\left(t\right)=0$ and $u_{b}^{*}\left(t\right)=0$] is

$${\hat{x}}_{k}=s_{k}\frac{B_{b}}{B_{k}}x_{b}^{*}\left(t_{a}\right),$$ (34)

where ${\hat{x}}_{k}$ is the asymptotic skill level, $x_{b}^{*}\left(t_{a}\right)$ is the adult brain mass, *s*_k_ is the fraction of brain metabolic rate allocated to energy-extraction skills, and *B*_b_ is the brain mass-specific maintenance cost. Hence, adult skill level is proportional to adult brain mass in the model (1) because of saturation with skill maintenance of the brain metabolic rate allocated to skills and (2) because adult brain metabolic rate is found to be proportional to adult brain mass given energy conservation and the assumptions on the parameters [setting ${\dot{x}}_{b}\left(t_{a}\right)=0$ in Eq (12) yields *B*_rest,b_(*t*_a_) = *x*_b_(*t*_a_)*B*_b_]. Weak correlations between cognitive ability and brain mass have been identified across taxa including humans [14, 103–106]. Since skill level is here broadly understood to refer to cognitive abilities, this result offers an explanation for these correlations in terms of saturation of brain metabolic rate with skill maintenance (memory).

In section 8.2.3 of S1 Appendix, we relax the assumption that maternal care is independent of maternal skill by allowing for such dependence (i.e., by letting maternal facilitation in Eq 33 depend on maternal skill). Doing so yields the same results with exponential competence and a slightly faster body growth rate with power competence (section 8.2.3 in S1 Appendix). The latter difference is only quantitative and arises because the chosen benchmark maternal facilitation for newborns (*φ*_0_ = 0.6) is lower than the resulting newborn maternal facilitation (*φ*_0_ = 0.8) when it is allowed to depend on maternal skill. In addition, the predicted adult body and brain mass are virtually the same with this relaxation for either the power or exponential competence (Fig H in S1 Appendix). Hence, our results are robust to modification of our simplifying assumption of exogenous maternal care.

We now vary parameter values to assess what factors favor a large brain at adulthood in a me-vs-nature setting.

### A large brain is favored by intermediate environmental difficulty, moderate skill effectiveness, and costly memory

**Fig 5:**
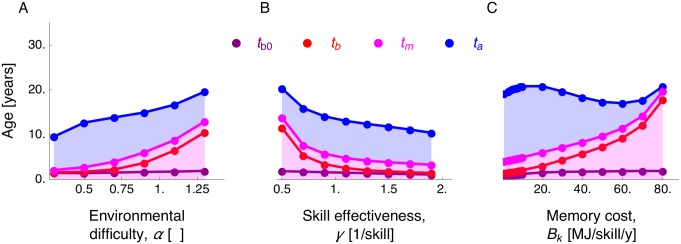
Predicted ages at brain growth onset (*t*_b0_), brain growth arrest (*t*_b_), maturity (*t*_m_), and adulthood (*t*_a_) as parameter values change with exponential competence. Plots show the predicted ages (switching times) vs (A) environmental difficulty, (B) skill effectiveness, and (C) memory cost. The childhood stage is shaded in pink, and the adolescence stage is shaded in blue. For clarity in the figure, points where adult body or brain sizes are zero are not shown (such points are shown in Fig 6).

We focus on varying the parameter values that were not estimated from empirical data to also assess how they impact predictions. For the switching times in the cases when the predicted adult body and brain mass are not zero, we find that the age of brain growth onset (*t*_b0_) remains largely invariant to parameter change (Fig 5 and Figs Q, S, U, and W in S1 Appendix). Later ages at brain growth arrest (*t*_b_) and at maturity (*t*_m_) are favored by increasing environmental difficulty (increasing *α*; Fig 5A and Fig S panel A in S1 Appendix), decreasing skill effectiveness (decreasing *γ*; Fig 5B and Fig S panel B in S1 Appendix), and costlier memory (increasing *B*_k_; Fig 5C and Fig S panel D in S1 Appendix). A later age at adulthood (*t*_a_) is also favored by environmental difficulty (Fig 5A and Fig S panel A in S1 Appendix) and skill ineffectiveness (Fig 5B and Fig S panel B in S1 Appendix), but is favored by either small or large memory costs (Fig 5C and Fig S panel D in S1 Appendix). While the switching times (*t*_b0_, *t*_b_, *t*_m_, *t*_a_) vary quantitatively as parameter values change, the qualitative occurrence of an adolescence period after childhood persists (shaded regions in Fig 5 and Figs Q, S, U, and W in S1 Appendix).

**Fig 6:**
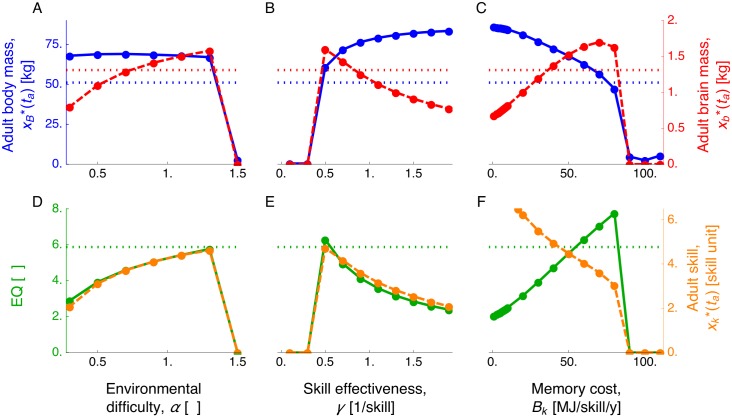
Large adult brain mass and EQ are favored by environmental difficulty, moderate skill effectiveness, and costly memory. Plots are the predicted adult body and brain mass, EQ, and skill vs parameter values with exponential competence. A-C show adult body mass (blue) and adult brain mass (red). D-F show adult EQ (green) and skill (orange). Vertical axes are in different scales. Dashed horizontal lines are the observed values in human females [96].

Regarding brain mass, a larger adult brain mass is favored by an increasingly challenging environment (increasing *α*; Eq 32), but is *disfavored* by an exceedingly challenging environment (Fig 6A). Environmental difficulty favors a larger brain because a higher skill level is needed for energy extraction (Eq 32), and from Eq (14) a higher skill level can be gained by increasing brain metabolic rate in turn by increasing brain mass. Thus, a large brain is favored to energetically support skill growth in a challenging environment. However, with exceedingly challenging environments, the individual is favored to reproduce early without substantial body or brain growth because it fails to gain a sufficiently high skill level to maintain its body mass as maternal care decreases with age (Fig O in S1 Appendix).

A larger adult brain is favored by moderately effective skills. When skills are ineffective at energy extraction (*γ* → 0; Eq 32), the brain entails little fitness benefit and fails to grow in which case the individual also reproduces without substantially growing (Fig 6B). When skill effectiveness (*γ*) crosses a threshold value, the fitness effect of brain becomes large enough that the brain becomes favored to grow. Yet, as skill effectiveness increases further and thus a lower skill level is sufficient for energy extraction, a smaller brain supports enough skill growth, so the optimal adult brain mass *decreases* with skill effectiveness (Fig 6B). Hence, adult brain mass is largest with moderately effective skill.

A larger brain is also favored by skills that are increasingly expensive for the brain to maintain (costly memory, increasing *B*_k_), but exceedingly costly memory prevents body and brain growth (Fig 6C). Costly memory favors a large brain because then a larger brain mass is required to energetically support skill growth (Eq 14). If memory is exceedingly costly, skill level fails to grow and energy extraction is unsuccessful, causing the individual to reproduce without substantial growth (Fig 6C).

### Factors favoring a large EQ and high skill

A large EQ and high adult skill are generally favored by the same factors that favor a large adult brain, but there are some differences regarding memory and learning costs. First, the memory cost has a particularly strong effect favoring a large EQ because it simultaneously favors increased brain and reduced body mass (Fig 6C and 6F). In contrast, increasing learning cost simultaneously reduces adult body and brain mass causing EQ to be invariant with respect to learning costs (Fig R panel D in S1 Appendix). That is, memory costs have a strong effect on EQ, but learning costs have little to no effect on it. Second, in contrast to its effect on EQ, increasing memory cost *disfavors* a high adult skill level (Fig 6F). That is, a higher EQ attained by increasing memory costs is accompained by a *decrease* in skill level (Fig 6C and 6F). The factors that favor a large brain, large EQ, and high skill are similar with either power or exponential competence (Fig 6 and Figs R and T in S1 Appendix). Importantly, although with the estimated parameter values the me-vs-nature setting can recover human growth patterns yielding adult body and brain mass of ancient humans, our exploration of the parameters that were not estimated from data suggests that the me-vs-nature setting cannot recover human growth patterns yielding adult body and brain mass of *modern* humans.

## Discussion

By combining elements of life history and metabolic theories, we formulated a metabolically explicit mathematical model to study brain life history evolution that can yield testable quantitative predictions from predefined settings. We applied our model to a me-vs-nature setting where individuals have no social interactions except possibly with their mothers through maternal care, but the model can be implemented to study brain evolution more generally. Surprisingly, our results for the me-vs-nature case show that this setting can be sufficient to generate major human life history stages as well as adult brain and body mass of ancient human scale, all without social interactions, evolutionary arms races in cognition triggered by social conflict, or explicit cumulative culture. Overall, we find that in the model the brain is favored to grow to energetically support skill growth, and thus a larger brain is favored when (1) competence at energy extraction has a steep dependence on skill, (2) high skill is needed for energy extraction due to environmental difficulty and moderate skill effectiveness, and (3) skills are expensive for the brain to maintain but are still necessary for energy extraction.

We find that somatic tissue grows more than the other tissues because it contributes to body mass and it is the cheapest tissue to maintain. The reason for this stems from the physical constraint imposed by resting metabolic rate being a power law of body mass (Eq 21). This constraint implies that individuals with a larger body mass release more heat and thus must compensate their energy budget either by extracting more energy, storing less energy, burning less energy in activity, or leaving less energy unassimilated (Eq 22). Given the pervasiveness across living systems of the power law between resting metabolic rate and body mass, we assumed that individuals balance their energy budget this way (as is implicitly assumed by [54]). If an individual balances its energy budget like this, then increasing any tissue’s mass can increase the energy available for growth, particularly if the tissue is cheap to maintain (so Eq 23 is positive). Thus, in our model all tissues have the implicit function of being able to increase the energy available for growth, given that individuals balance their energy budget as indicated, and this is why somatic tissue grows in our model.

The model correctly divides the individual’s lifespan into childhood, adolescence, and adulthood. The model also rightly predicts brain growth to occur only during childhood, although there is a delay in the predicted brain growth which may be due to the absence of social interactions or an underestimation of resting metabolic rate early in life by its power law approximation. Additionally, the predicted childhood stage finishes with a body growth spurt that ends at the age of maturity, as observed in human preadolescence and menarche. The model also recovers an adolescence stage with simultaneous allocation to growth and reproduction, which has been previously difficult to replicate with life history models [32]. While the timing of these predicted life stages depends on the magnitude of parameter values, their relative sequence is robust to change in the parameter values that were not estimated from data. However, the childhood-adolescence-adulthood sequence may depend on the relative magnitude of metabolic costs of maintenance and production of the different tissues (i.e., on whether (1) *B*_*i*_ < *B*_*j*_ and (2) *E*_*i*_ < *E*_*j*_ for *i*, *j* ∈ {b, r, s}). Empirically guided refinement of both parameter values and the shape of energy-extraction efficiency is expected to allow for increasingly accurate predictions [91]. Similarly, empirical data for non-human taxa should allow determining the model’s ability to predict diverse life histories and brain growth patterns [107–109].

The model also offers a metabolic explanation for correlations between adult cognitive ability and brain mass observed in inter- and intraspecific studies in birds and mammals including humans [14, 103–106]. The explanation is that when skills are costly for the brain to maintain (costly memory), the brain metabolic rate allocated to skills can become saturated by the energetic consumption of skill maintenance which causes adult skill level (or cognitive ability) to be proportional to brain mass (Eq 34). This proportionality between adult skill level and adult brain mass arises because the adult brain metabolic rate is found to be proportional to brain mass. This follows from energy conservation and our assumption that some of the brain (resting) metabolic rate is due to skill learning and memory (Eq 13), which does not make any assumptions about skill function. Consequently, the proportionality between adult skill level and brain mass is linear rather than non-linear: adding exponents to brain mass and skill level in Eqs (12) and (13) would remove the linear relationship between adult brain mass and adult skill, but it would also violate the assumption of energy conservation given the definitions of the accompanying parameters. Eq (34) also predicts that additional variation in correlations between cognitive ability and brain mass can be explained by variation in maintenance costs of brain and skill (see [110]), and by variation in brain metabolic rate allocation to skill. However, the proportionality between adult skill and adult brain mass arising from Eq (34) assumes that the fraction of brain metabolic rate allocated to the skills of interest (*s*_k_) is independent of brain mass (and similarly for *B*_b_ and *B*_k_). So, the proportionality could break if any of these parameters depends on brain mass. Also, the model indicates that adult skill and brain mass need not be correlated since saturation with skill maintenance of the brain metabolic rate allocated to skills may not occur during the individual’s lifespan, for example if memory is inexpensive, so skill increases throughout life (Fig K panel E in S1 Appendix).

Predicted adult brain mass and skill have non-monotonic relationships with their predictor variables (Fig 6 and Figs R and T in S1 Appendix). Consequently, conflicting inferences can be drawn if predictor variables are evaluated only on their low or high ends. For instance, increasingly challenging environments favor large brains up to a point, so that exceedingly challenging environments disfavor large brains. Thus, on the low end of environmental difficulty, the prediction that increasingly challenging environments favor large brains is consistent with ecological challenge hypotheses [45, 46, 111]; yet, on the high end of environmental difficulty, the prediction that increasingly challenging environments disfavor large brains is consistent with constraint hypotheses according to which facilitation of environmental challenge favors larger brains [46, 112–114]. Counter-intuitively on first encounter, the finding that moderately effective skills are most conducive to a large brain and high skill is a consequence of the need of higher skill when skill effectiveness decreases (Fig 6B). Regarding memory cost, the strong effect of memory cost on favoring a high EQ at first glance suggests that a larger EQ than that observed in humans is possible if memory were costlier (see dashed lines in Fig 6F). However, such larger memory costs cause a reduced adult skill level (Fig 6F), and a substantial delay in body and brain growth that yields growth patterns that are inconsistent with those of humans (Figs L–N in S1 Appendix).

We have made a number of assumptions that, if modified, the predicted relationships may be qualitatively affected, as is known to occur with life history models [33]. Assumptions whose modification may qualitatively affect predictions, as suggested by previous life-history models, include (1) different forms for the mortality rate (e.g., as a function of skills) [69, 115], (2) different modes of population regulation (e.g., through survival rather than through fecundity) [69, 115], and (3) allowing population size to change [62, 115]. Implementing modifications like these would allow to assess the level of generality of our results. Additionally, our model may be used to study allocation to tissue maintenance to address senescence.

Although our model does not include numerous details relevant to human life history, such as social interactions and cummulative social learning, our results are relevant for a set of hypotheses for human-brain evolution. In particular, food processing (e.g., mechanically with stone tools or by cooking) has previously been advanced as a determinant factor in human-brain evolution as it increases energy and nutrient availability from otherwise relatively inaccessible sources [7, 116]. This is supported by archaeological evidence of mechanical food processing in early humans (1.5 mya in Kenya; [116, 117]), as well as archeological evidence of fire control in early humans with increasing certainty in younger strata (1.5 mya–130 kya; [118–120]). Our results, where individuals engage in energy extraction without considering the effects of social interactions, suggest that food processing alone could indeed have been sufficient to allow for a substantial brain expansion. In addition, food processing may help satisfy at least two of the three key conditions identified for large-brain evolution listed in the first paragraph of the Discussion. First, a shift in food-processing technology (e.g., from primarily mechanical to cooking) could create a steeper relationship between energy-extraction skill and competence by substantially facilitating energy extraction (relating to condition 1). Second, food processing (e.g., by building the required tools or lighting a fire) is a challenging feat to learn and may often fail (relating to condition 2). Yet, there are scant data allowing to judge the metabolic expense for the brain to maintain tool-making or fire-control skills (condition 3). Our results are thus consistent with the hypothesis of food processing as being a key factor in human brain expansion.

In sum, the model identifies various conditions favoring large-brain evolution, in particular steep competence with respect to skill, intermediate environmental difficulty, moderate skill effectiveness, and costly memory. As we did not consider social interactions, our application of the model cannot refute or support social brain hypotheses. However, application of our model to the social realm should allow for assessments of social hypotheses. Overall, our model is a step towards a quantitative theory of brain life history evolution yielding testable quantitative predictions as ecological, demographic, and social factors vary.

## Supporting information

S1 Appendix Appendix. Analytical results, parameter estimation, numerical implementation, and supplementary numerical results.
(PDF)

S1 Computer code Computer code. MATLAB computer code for solutions using GPOPS.
(ZIP)
