# Supplementary material for: A model for brain life history evolution
Source: PLoS Comput Biol. 2017 Mar 9;13(3):e1005380. doi: 10.1371/journal.pcbi.1005380 (PMC5344330; doi:10.1371/journal.pcbi.1005380)
Supplement: S1 Appendix — Analytical results, parameter estimation, numerical implementation, and supplementary numerical results. (PDF) [file pcbi.1005380.s001.pdf]

S1 Appendix for:

A model for brain life history evolution

Mauricio González-Forero, Timm Faulwasser, and Laurent Lehmann

Contents

1 Optimal control problem **3**

2 The Pontryagin Maximum Principle **4**

3 Analytical results **7**

4 Derivation of analytical results **12**

4.1 Calculation of $\dot{\psi}_i$ 12

4.2 Singular controls for regime BS: $\sigma_\mathrm{s} > 0$, $\sigma_\mathrm{b} > 0$, and $\sigma_\mathrm{s} = \sigma_\mathrm{b}$ 14

4.3 Singular controls for regime BR: $\sigma_\mathrm{s} < 0$ and $\sigma_\mathrm{b} = 0$ 15

4.4 Singular controls for regime RS: $\sigma_\mathrm{s} = 0$ and $\sigma_\mathrm{b} < 0$ 16

4.5 Singular controls for regime BRS: $\sigma_\mathrm{s} = \sigma_\mathrm{b} = 0$ 16

5 Parameter values **19**

6 Estimation of parameter values **20**

6.1 Values for B_i for $i \in \{\mathrm{b}, \mathrm{r}, \mathrm{s}\}$ 20

6.2 Values for E_i for $i \in \{\mathrm{b}, \mathrm{r}, \mathrm{s}\}$ 21

6.3 Values for K and β 21

6.4 Values for f_0, μ, and T 21

7 Numerical implementation **23**

8 Supplementary results **24**

8.1 Results without image filtering 24

8.2 Tests of assumptions 24

8.2.1 Effect of fecundity approximation 24

8.2.2 Effect of removing maternal care 28

8.2.3 Relaxing the assumption that maternal care is independent of maternal skill 29

8.3 Brain metabolic rate through ontogeny 31

8.4 Mass of reproductive tissue 32

8.5 Indeterminate skill growth with inexpensive memory 32

8.6 Large, yet inconsistent-with-data encephalization with exceedingly expensive memory 33
8.7 Reproduction without growth and body collapse for certain parameter values 35
8.8 A large brain is also favored by small metabolic costs of learning, small innate skill, and intermediate allocation of brain metabolic rate to skills 36
8.9 Dependence on maternal care parameters 39

References 39

1 Optimal control problem

From our assumptions of no social interactions and density-dependent competition through fecundity described in the section "Closing the model" (see Eqs. 25 and 31; hereafter all equations refer to those in the main text unless they have an "S"), the basic reproductive number (Eq. 2) can be written as

$$R_0(\mathbb{u},\mathbb{v}) = C(\mathbb{v})J(\mathbb{u}), \tag{S1}$$

for some scalar $C(\mathbb{v}) > 0$ and objective function

$$J(\mathbb{u}) = f_0 \int_0^T \exp(-\mu t) x_\mathrm{r}(t) \mathrm{d}t, \tag{S2}$$

which only depends on the mutant but not the resident strategy (we leave f_0 in the objective to re-scale it, which facilitates numerical convergence). Hence, determining an uninvadable strategy (Eq. 1) poses a standard life history optimal control problem [1–5].

In the terminology of optimal control theory (e.g., [5]), we seek a strategy $\mathbb{u}^*$ and resulting $\mathbb{x}^*$ that solve the problem

$$\max_{\mathbb{u}\in\mathbb{U}} J(\mathbb{u}), \tag{S3a}$$

where $\mathbb{u} = \{\mathbf{u}(t)\}_{t=0}^T$ is the time sequence of control variables subject to the constraints

$$\mathbf{u}(t) = (u_\mathrm{b}(t), u_\mathrm{r}(t), u_\mathrm{s}(t)) \in [0,1]^3 \quad \text{and} \quad u_\mathrm{b}(t) + u_\mathrm{r}(t) + u_\mathrm{s}(t) = 1. \tag{S3b}$$

Likewise, $\mathbb{x} = \{\mathbf{x}(t)\}_{t=0}^T$ is the time sequence of state variables

$$\mathbf{x}(t) = (x_\mathrm{b}(t), x_\mathrm{r}(t), x_\mathrm{s}(t), x_\mathrm{k}(t)) \geq 0, \tag{S3c}$$

for all t in the time interval $[0, T]$. For readability, we will suppress the argument in $\mathbf{u}(t)$ and $\mathbf{x}(t)$, and instead simply write $\mathbf{u}$ and $\mathbf{x}$ below. Note that referring to $\mathbb{u}$ and $\mathbb{x}$ as time sequences is a slight abuse of optimal control terminology, where they would be called control signal (or trajectory) and state trajectory, respectively. With this notation, the dynamic constraints are

$$\dot{\mathbf{x}} = \mathbf{g}(t,\mathbf{u},\mathbf{x}), \tag{S3d}$$

with

$$g_i(t,\mathbf{u},\mathbf{x}) = a_i u_i B_\mathrm{syn}(t,\mathbf{x}) \quad \text{for } i \in \{\mathrm{b},\mathrm{r},\mathrm{s}\} \tag{S3e}$$

$$g_\mathrm{k}(t,\mathbf{u},\mathbf{x}) = b_1 \left[x_\mathrm{b} B_\mathrm{b} + u_\mathrm{b} B_\mathrm{syn}(t,\mathbf{x})\right] - b_2 x_\mathrm{k}, \tag{S3f}$$

which are obtained from Eqs. (24), where $a_i = 1/E_i$, $b_1 = s_\mathrm{k}/E_\mathrm{k}$, and $b_2 = B_\mathrm{k}/E_\mathrm{k}$. From Eqs. (6), (11), and (21), we have that the growth metabolic rate is

$$B_\mathrm{syn}(t,\mathbf{x}) = K e(t, x_\mathrm{k}) x_\mathrm{B}^\beta - B_\mathrm{b} x_\mathrm{b} - B_\mathrm{r} x_\mathrm{r} - B_\mathrm{s} x_\mathrm{s}, \tag{S3g}$$

where body mass is

$$x_\mathrm{B} = x_\mathrm{b} + x_\mathrm{r} + x_\mathrm{s}, \tag{S3h}$$

and the energy extraction efficiency at age t is

$$e(t, x_\mathrm{k}) = \frac{c(x_\mathrm{k})}{\alpha[1 - \varphi_0 \exp(-\varphi_\mathrm{r} t)] + c(x_\mathrm{k})}, \tag{S3i}$$

which depends on the competence at energy extraction:

$$c(x_{\mathrm{k}}) = \begin{cases} x_{\mathrm{k}}^{\gamma} & \text{(power competence)} \\ \exp(\gamma x_{\mathrm{k}}) & \text{(exponential competence).} \end{cases} \tag{S3j}$$

Finally, the initial conditions of Eq. (S3d) are

$$x_i(0) = x_{i0} \quad \text{for all } i \tag{S3k}$$

and we do not consider any terminal conditions for Eq. (S3d).

2 The Pontryagin Maximum Principle

Necessary first-order conditions for maximizing the objective $J(\mathbb{u})$ with respect to the controls throughout t are given by the Pontryagin Maximum Principle (PMP) [5–7]. The PMP states that if $(\mathbb{u}^*, \mathbb{x}^*)$ is a solution to the optimal control problem (S3), then an associated function, the Hamiltonian, is maximized with respect to the controls when evaluated at $(\mathbf{u}^*, \mathbf{x}^*)$ for all $t \in [0, T]$. The Hamiltonian for problem (S3) is

$$H(t, \mathbf{u}, \mathbf{x}, \boldsymbol{\lambda}) = f_0 \exp(-\mu t) x_{\mathrm{r}} + \sum_{i \in \{\mathrm{b,r,s,k}\}} \lambda_i g_i(t, \mathbf{u}, \mathbf{x}), \tag{S4}$$

where λ_i is the costate variable associated to state variable i and $\boldsymbol{\lambda}$ is the vector of costates. Here we also drop the argument of $\lambda_i(t)$ and write simply λ_i. A costate variable gives the marginal value of the corresponding state variable; that is, it is the effect on the maximized objective J^* for a marginal change in the corresponding state variable [8]. Thus, we now proceed to maximize the Hamiltonian to obtain candidate optimal controls $\mathbf{u}^*$ that satisfy these necessary conditions for optimality. To simplify the mathematical exposition of the PMP, the following derivations focus on the case of inactive state constraints; that is, we assume that Eq. (S3c) holds with the strict inequality. This simplification is justified since for any interval $[t_1, t_2] \subset [0, T]$ with $x_i(t) = 0$ for $i \in \{\mathrm{b,r,s}\}$, we have that $u_i(t) = 0$ (see Eq. S3e). Moreover, our numerical solutions indicate that $x_{\mathrm{k}}(t)$ is never identically zero along the optimal solutions (e.g., Fig. 4 in the main text). For an overview of the the PMP for optimal control problems with state constraints, see [9].

For simplicity of presentation in the remainder of sections 2-4, we will explicitly write the arguments of a function only when defining the function and will suppress their writing elsewhere, except in a few places where it is useful to recall them.

Due to the constraint $u_{\mathrm{b}} + u_{\mathrm{r}} + u_{\mathrm{s}} = 1$ (Eq. S3b), we set $u_{\mathrm{r}} = 1 - u_{\mathrm{b}} - u_{\mathrm{s}}$ and only two controls must be determined: u_{b}^* and u_{s}^*. Using Eqs. (S3e) and (S3f), collecting for B_{syn} in Eq. (S4), and evaluating at $(\mathbf{x}, \boldsymbol{\lambda}) = (\mathbf{x}^*, \boldsymbol{\lambda}^*)$ the Hamiltonian becomes

$$H(t, \mathbf{u}, \mathbf{x}^*, \boldsymbol{\lambda}^*) = f_0 \exp(-\mu t) x_{\mathrm{r}}^* + B_{\mathrm{syn}}(t, \mathbf{x}^*) \phi(\mathbf{u}, \boldsymbol{\lambda}^*) + \lambda_{\mathrm{k}}^* \xi(\mathbf{x}^*), \tag{S5}$$

where

$$\phi(\mathbf{u}, \boldsymbol{\lambda}) = u_{\mathrm{b}} \sigma_{\mathrm{b}} + u_{\mathrm{s}} \sigma_{\mathrm{s}} + a_{\mathrm{r}} \lambda_{\mathrm{r}} \tag{S6a}$$

$$\xi(\mathbf{x}) = b_1 x_{\mathrm{b}} B_{\mathrm{b}} - b_2 x_{\mathrm{k}} \tag{S6b}$$

and

$$\sigma_\mathrm{b}(\boldsymbol{\lambda}) = a_\mathrm{b}\lambda_\mathrm{b} - a_\mathrm{r}\lambda_\mathrm{r} + b_1\lambda_\mathrm{k} \tag{S7a}$$

$$\sigma_\mathrm{s}(\boldsymbol{\lambda}) = a_\mathrm{s}\lambda_\mathrm{s} - a_\mathrm{r}\lambda_\mathrm{r}. \tag{S7b}$$

We thus seek to maximize Eq. (S5) with respect to $\mathbf{u} = (u_\mathrm{b}, u_\mathrm{s})$.

The derivatives of the Hamiltonian (S5) with respect to the two controls (u_b, u_s) are (see Eq. 10 on p. 126 of [7])

$$\left.\frac{\partial H(t,\mathbf{u},\mathbf{x}^*,\boldsymbol{\lambda}^*)}{\partial u_i}\right|_{\mathbf{u}=\mathbf{u}^*} = B_\mathrm{syn}\sigma_i \qquad \text{for } i \in \{\mathrm{b},\mathrm{s}\}. \tag{S8}$$

If $B_\mathrm{syn} > 0$, then the Hamiltonian is maximized with respect to u_b and u_s depending on the signs of the switching functions σ_i and, because of the constraint that $u_\mathrm{b} + u_\mathrm{s} \leq 1$ (Eq. S3b), also depending on the sign of the difference

$$\sigma_\mathrm{s} - \sigma_\mathrm{b} = a_\mathrm{s}\lambda_\mathrm{s} - a_\mathrm{b}\lambda_\mathrm{b} - b_1\lambda_\mathrm{k}. \tag{S9}$$

By definition, the costates satisfy (see Eq. 7 on p. 126 of [7])

$$\dot{\lambda}_i^* = -\left.\frac{\partial H(\mathbf{u}^*,\mathbf{x},\boldsymbol{\lambda}^*,t)}{\partial x_i}\right|_{\mathbf{x}=\mathbf{x}^*} \qquad \text{for } i \in \{\mathrm{b},\mathrm{r},\mathrm{s},\mathrm{k}\} \tag{S10a}$$

$$\lambda_i^*(T) = 0. \tag{S10b}$$

For readability, hereafter we write $\boldsymbol{\lambda}$ instead of $\boldsymbol{\lambda}^*$. Hence, the dynamical equations of the costates are

$$\dot{\lambda}_\mathrm{b} = -\left(\phi\psi_\mathrm{b} + \lambda_\mathrm{k} b_1 B_\mathrm{b}\right) \tag{S11a}$$

$$\dot{\lambda}_\mathrm{r} = -\left(\phi\psi_\mathrm{r} + f_0 \exp(-\mu t)\right) \tag{S11b}$$

$$\dot{\lambda}_\mathrm{s} = -\phi\psi_\mathrm{s} \tag{S11c}$$

$$\dot{\lambda}_\mathrm{k} = -\left(\phi\psi_\mathrm{k} - \lambda_\mathrm{k} b_2\right), \tag{S11d}$$

evaluated at $(\mathbf{x}^*, \mathbf{u}^*)$, where we define

$$\psi_i(t,\mathbf{x}^*) = \left.\frac{\partial B_\mathrm{syn}}{\partial x_i}\right|_{\mathbf{x}=\mathbf{x}^*} \tag{S12}$$

for $i \in \{\mathrm{b},\mathrm{r},\mathrm{s},\mathrm{k}\}$ (see Eq. 23 in the main text).

Then,

$$\psi_i(t,\mathbf{x}^*) = \psi(t,\mathbf{x}^*) - B_i \qquad \text{for } i \in \{\mathrm{b},\mathrm{r},\mathrm{s}\} \tag{S13a}$$

$$\psi_\mathrm{k}(t,\mathbf{x}^*) = K x_\mathrm{B}^\beta(\mathbf{x}^*)\frac{\partial e}{\partial x_\mathrm{k}} \tag{S13b}$$

$$= K x_\mathrm{B}^\beta(\mathbf{x}^*) e(t,x_\mathrm{k}^*)[1 - e(t,x_\mathrm{k}^*)]\frac{\gamma}{\delta(x_\mathrm{k}^*)}, \tag{S13c}$$

where

$$\psi(t,\mathbf{x}^*) = K\beta e(t,x_\mathrm{k}^*) x_\mathrm{B}^{\beta-1}(\mathbf{x}^*) \tag{S13d}$$

$$\delta(x_\mathrm{k}^*) = \begin{cases} x_\mathrm{k}^* & \text{for } c(x_\mathrm{k}) = x_\mathrm{k}^\gamma \\ 1 & \text{for } c(x_\mathrm{k}) = \exp(\gamma x_\mathrm{k}). \end{cases} \tag{S13e}$$

Eq. (S13c) follows because the marginal returns on energy extraction from increasing skill are

$$\frac{\partial e}{\partial x_\mathrm{k}} = e(1-e)\frac{\mathrm{d}\ln c(x_\mathrm{k})}{\mathrm{d}x_\mathrm{k}} \tag{S14a}$$

$$= e(1-e)\frac{\gamma}{\delta(x_\mathrm{k})}. \tag{S14b}$$

3 Analytical results

We present the analytical results for the candidate optimal controls in this section, and their derivations in section 4. In these two sections, we assume that the growth metabolic rate is positive; that is, $B_{\mathrm{syn}}(t, \mathbf{x}^*) > 0$.

The Hamiltonian of the optimal control problem (S3) is affine (or, less rigorously, linear) in the controls (Eq. S5). Since we assume that $B_{\mathrm{syn}}(t, \mathbf{x}^*) > 0$, the sign of the derivative of the Hamiltonian with respect to u_{s} or u_{b} (Eq. S8) is given by the sign of the two switching functions σ_{s} and σ_{b} (Eqs. S7). If σ_i is negative, the Hamiltonian is maximized when $u_i^* = 0$. If σ_i is positive and the other switching function, denoted by $\sigma_{i'}$, is negative, then the Hamiltonian is maximized when $(u_i^*, u_{i'}^*) = (1, 0)$. If both σ_i and $\sigma_{i'}$ are positive, because of the constraint that $u_{\mathrm{s}}^* + u_{\mathrm{b}}^* \leq 1$, the Hamiltonian is maximized when $(u_i^*, u_{i'}^*) = (1, 0)$ if and only if $\sigma_i > \sigma_{i'}$. If σ_i is zero and $\sigma_{i'}$ is positive, then the Hamiltonian is maximized when $(u_i^*, u_{i'}^*) = (0, 1)$. If σ_i is zero and $\sigma_{i'}$ is negative, then the Hamiltonian is maximized when $u_{i'}^* = 0$ but the Hamiltonian is independent of u_i. In this case, the candidate optimal control $u_i^* = \hat{u}_i$ is called a singular arc and must be determined by another method [6]. If both σ_{s} and σ_{b} are zero, the Hamiltonian is independent of both controls and the candidate optimal controls are the singular arcs $(u_{\mathrm{s}}^*, u_{\mathrm{b}}^*) = (\hat{u}_{\mathrm{s}}, \hat{u}_{\mathrm{s}})$. Finally, if both σ_{s} and σ_{b} are positive and equal, then both u_{s}^* and u_{b}^* are positive and maximal given the constraint $u_{\mathrm{s}}^* + u_{\mathrm{b}}^* \leq 1$, so $(u_{\mathrm{s}}^*, u_{\mathrm{b}}^*) = (1 - \hat{u}_{\mathrm{b}}, \hat{u}_{\mathrm{b}})$.

Together, these cases show that there are seven possible growth regimes (Table A). Regimes B, R, and S involve pure growth of one of the three tissues, whereas regimes BS, BR, RS, and BRS are singular arcs where at least two tissues grow simultaneously. These regimes occur as indicated in Table A depending on the sign of both the switching functions and their difference. Numerical illustration of these regimes is given in Fig. A (figures and tables with alphabetic labels are in the S1 Appendix; those with numeric labels are in the main text).

Regime	Tissues growing	Candidate optimal controls	Sign of switching functions
		$(u_{\mathrm{s}}^*, u_{\mathrm{b}}^*)$	$\mathrm{sign}(\sigma_{\mathrm{s}}, \sigma_{\mathrm{b}}, \sigma_{\mathrm{s}} - \sigma_{\mathrm{b}})$
R	Reproductive	$(0, 0)$	$(-, -, \cdot)$
B	Brain	$(0, 1)$	$(-, +, \cdot)$, $(+, +, -)$, $(0, +, \cdot)$
S	Soma	$(1, 0)$	$(+, -, \cdot)$, $(+, +, +)$, $(+, 0, \cdot)$
BS	Brain and soma	$(1 - \hat{u}_{\mathrm{b}}, \hat{u}_{\mathrm{b}})$	$(+, +, 0)$
BR	Brain and reproductive	$(0, \hat{u}_{\mathrm{b}})$	$(-, 0, \cdot)$
RS	Reproductive and soma	$(\hat{u}_{\mathrm{s}}, 0)$	$(0, -, \cdot)$
BRS	Brain, reproductive, and soma	$(\hat{u}_{\mathrm{s}}, \hat{u}_{\mathrm{b}})$	$(0, 0, \cdot)$

Table A: Growth regimes. Four regimes are singular arcs. Note that $u_{\mathrm{r}}^* = 1 - u_{\mathrm{s}}^* - u_{\mathrm{b}}^*$. The "$\cdot$" means any sign.

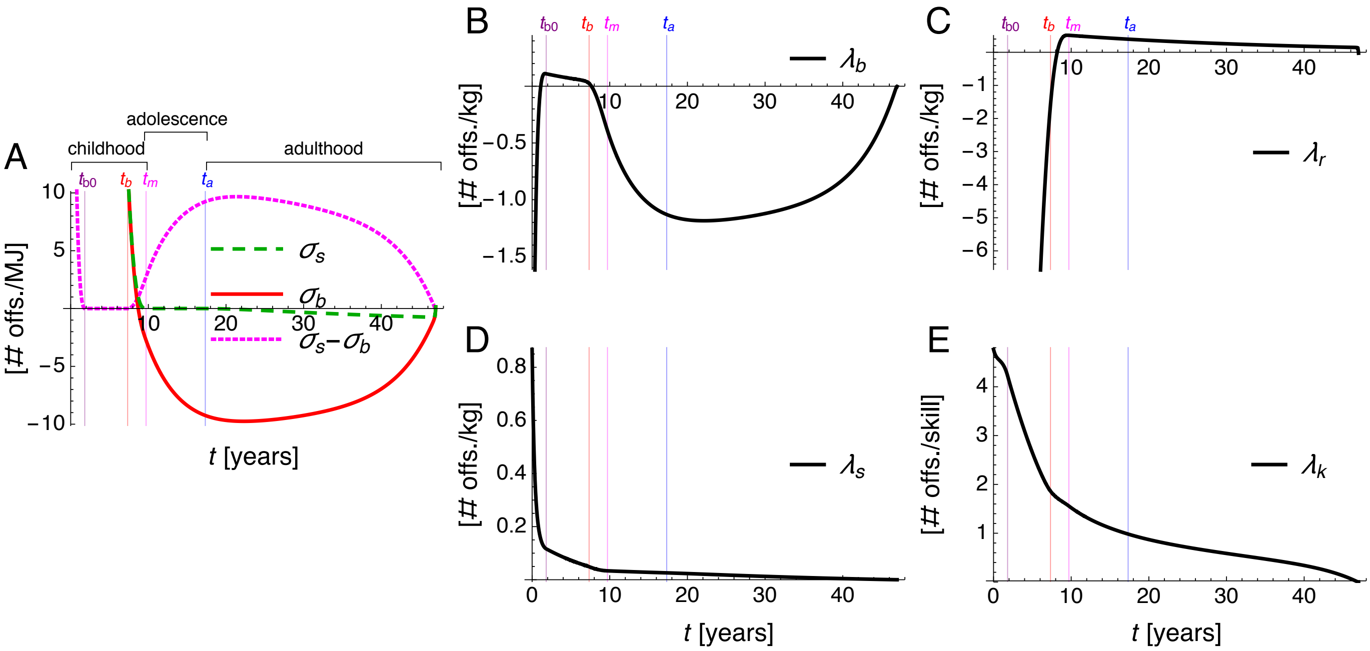

Figure A: Switching functions and costates for the process in Fig. 3 in the main text. The signs of the switching functions $\sigma_i(t)$ determine the candidate optimal allocation at time t (Table A). GPOPS yields the costates λ_i using a direct approach rather than the PMP [10]. The switching functions σ_i are calculated using such λ_i and Eqs. (S7). The costate $\lambda_i(t)$ gives the marginal value of state variable i at time t: that is, how much the lifetime number of offspring changes if state variable i increases by an infinitesimally small amount at time t. The switching function $\sigma_i(t)$ is proportional to the marginal effect on current and future profits of control variable i at time t: that is, proportional to the change in current offspring production and value of future prospects if control variable i increases by an infinitesimally small amount at time t. No filtering is applied to this figure.

In section 4 we show that for the singular arcs and assuming the denominators are non-zero, the candidate optimal controls are

$$\text{Regime BS:} \quad \hat{u}_{\mathrm{b}}(t,\mathbf{x}^*,\boldsymbol{\lambda}) = \frac{\rho_{\mathrm{sk}} - \chi^{\mathrm{sb}}_{\mathrm{s(s+/)(bk-sk)}}}{\chi^{\mathrm{sb}}_{\mathrm{s(s+/)(sk+/k)}}} \tag{S15a}$$

$$\text{Regime BR:} \quad \hat{u}_{\mathrm{b}}(t,\mathbf{x}^*,\boldsymbol{\lambda}) = \frac{\rho_{\mathrm{rk}} - \chi^{\mathrm{br}}_{\mathrm{r/(/k)}}}{\chi^{\mathrm{br}}_{\mathrm{rb(bk)}}} \tag{S15b}$$

$$\text{Regime RS:} \quad \hat{u}_{\mathrm{s}}(t,\mathbf{x}^*,\boldsymbol{\lambda}) = \frac{\rho_{\mathrm{rs}} - \chi^{\mathrm{sr}}_{\mathrm{r/0}}}{\chi^{\mathrm{sr}}_{\mathrm{rs0}}} \tag{S15c}$$

$$\text{Regime BRS:} \quad \hat{u}_{\mathrm{s}}(t,\mathbf{x}^*,\boldsymbol{\lambda}) = \frac{1}{D}\left[(\rho_{\mathrm{rs}} - \chi^{\mathrm{sr}}_{\mathrm{r/0}})\chi^{\mathrm{br}}_{\mathrm{rb(bk)}} - (\rho_{\mathrm{rk}} - \chi^{\mathrm{br}}_{\mathrm{r/(/k)}})\chi^{\mathrm{sr}}_{\mathrm{rb0}}\right] \tag{S15d}$$

$$\hat{u}_{\mathrm{b}}(t,\mathbf{x}^*,\boldsymbol{\lambda}) = \frac{1}{D}\left[(\rho_{\mathrm{rk}} - \chi^{\mathrm{br}}_{\mathrm{r/(/k)}})\chi^{\mathrm{sr}}_{\mathrm{sr0}} - (\rho_{\mathrm{rs}} - \chi^{\mathrm{sr}}_{\mathrm{s/0}})\chi^{\mathrm{br}}_{\mathrm{rs(sk)}}\right] \tag{S15e}$$

$$D = \chi^{\mathrm{sr}}_{\mathrm{rs0}}\chi^{\mathrm{br}}_{\mathrm{rb(bk)}} - \chi^{\mathrm{sr}}_{\mathrm{rb0}}\chi^{\mathrm{br}}_{\mathrm{rs(sk)}}.$$

Here we have

$$\chi^{kl}_{ijm}(t,\mathbf{x}^*,\boldsymbol{\lambda}) = a_i\lambda_i\left[\omega_j(a_k - a_l) + \omega_m b_1\right] \tag{S16a}$$

$$\rho_{ni}(t,\mathbf{x}^*,\boldsymbol{\lambda}) = \theta_i\Big(\eta_n b_1 b_2 \lambda_{\mathrm{k}}(a_{\mathrm{b}}B_{\mathrm{b}} - b_2) \tag{S16b}$$

$$+ a_i\lambda_i\left\{a_i\psi_i\left[a_i\psi_i - \eta_n(a_{\mathrm{b}}\psi_{\mathrm{b}} + b_1\psi_{\mathrm{k}}) - \hat{\eta}_n a_{\mathrm{s}}\psi_{\mathrm{s}}\right] - \eta_n b_1\psi_{\mathrm{k}}(a_{\mathrm{b}}B_{\mathrm{b}} - b_2)\right\} \tag{S16c}$$

$$+ \hat{\theta}_i a_{\mathrm{r}} f_0 \exp(-\mu t)\left\{\mu + \left[a_{\mathrm{r}}\psi_{\mathrm{r}} - \eta_n\left(a_{\mathrm{b}}\psi_{\mathrm{b}} + b_1\psi_{\mathrm{k}}\right) - \hat{\eta}_n a_{\mathrm{s}}\psi_{\mathrm{s}}\right]\right\}\Big), \tag{S16d}$$

for

$$i \in \{\mathrm{r},\mathrm{s}\}, \quad k \in \{\mathrm{b},\mathrm{s}\}, \quad l \in \{\mathrm{b},\mathrm{r}\}, \quad n \in \{\mathrm{k},0\}, \tag{S17a}$$

$$j \in \{\mathrm{b},\mathrm{s},/,\mathrm{b}-\mathrm{s},\mathrm{s}+/\}, \quad m \in \{\mathrm{bk},\mathrm{sk},/\mathrm{k},0,\mathrm{bk}-\mathrm{sk},\mathrm{sk}+/\mathrm{k}\}. \tag{S17b}$$

From Eq. (S15), note that the superscripts in χ^{kl}_{ijm} coincide with the growth regime. The functions defining χ^{kl}_{ijm} and ρ_{ni} are

$$\omega_{\mathrm{s}}(t,\mathbf{x}^*) = \frac{\psi(t,\mathbf{x}^*)}{x_{\mathrm{B}}(\mathbf{x}^*)}B_{\mathrm{syn}}(t,\mathbf{x}^*)(\beta-1)(a_{\mathrm{s}} - a_{\mathrm{r}}) \tag{S18a}$$

$$\omega_{\mathrm{b}}(t,\mathbf{x}^*) = \frac{\psi(t,\mathbf{x}^*)}{x_{\mathrm{B}}(\mathbf{x}^*)}B_{\mathrm{syn}}(t,\mathbf{x}^*)\left[(\beta-1)(a_{\mathrm{b}} - a_{\mathrm{r}}) + x_{\mathrm{B}}(\mathbf{x}^*)(1 - e(t,x^*_{\mathrm{k}}))\frac{\gamma}{\delta(x^*_{\mathrm{k}})}b_1\right] \tag{S18b}$$

$$\omega_{/}(t,\mathbf{x}^*) = \frac{\psi(t,\mathbf{x}^*)}{x_{\mathrm{B}}(\mathbf{x}^*)}\left[B_{\mathrm{syn}}(t,\mathbf{x}^*)(\beta-1)a_{\mathrm{r}} + x_{\mathrm{B}}(\mathbf{x}^*)(1 - e(t,x^*_{\mathrm{k}}))\left[\frac{\gamma}{\delta(x^*_{\mathrm{k}})}\xi(\mathbf{x}^*) - \varphi_{\mathrm{r}}\frac{\alpha - d(t)}{d(t)}\right]\right] \tag{S18c}$$

$$\omega_{\mathrm{sk}}(t,\mathbf{x}^*) = \frac{\psi_{\mathrm{k}}(t,\mathbf{x}^*)}{x_{\mathrm{B}}(\mathbf{x}^*)}B_{\mathrm{syn}}(t,\mathbf{x}^*)\beta(a_{\mathrm{s}} - a_{\mathrm{r}}) \tag{S18d}$$

$$\omega_{\mathrm{bk}}(t,\mathbf{x}^*) = \frac{\psi_{\mathrm{k}}(t,\mathbf{x}^*)}{x_{\mathrm{B}}(\mathbf{x}^*)}B_{\mathrm{syn}}(t,\mathbf{x}^*)\left\{\beta(a_{\mathrm{b}} - a_{\mathrm{r}}) + x_{\mathrm{B}}(\mathbf{x}^*)b_1\left[(1 - 2e(t,x^*_{\mathrm{k}}))\frac{\gamma}{\delta(x^*_{\mathrm{k}})} - \hat{\delta}\delta(x^*_{\mathrm{k}})\frac{1}{x^{*2}_{\mathrm{k}}}\right]\right\} \tag{S18e}$$

$$\omega_{/\mathrm{k}}(t,\mathbf{x}^*) = \frac{\psi_{\mathrm{k}}(t,\mathbf{x}^*)}{x_{\mathrm{B}}(\mathbf{x}^*)}\left\{B_{\mathrm{syn}}(t,\mathbf{x}^*)\beta a_{\mathrm{r}} + x_{\mathrm{B}}(\mathbf{x}^*)(1 - 2e(t,x^*_{\mathrm{k}}))\left[\frac{\gamma}{\delta(x^*_{\mathrm{k}})}\xi(\mathbf{x}^*) - \varphi_{\mathrm{r}}\frac{\alpha - d(t)}{d(t)}\right] - x_{\mathrm{B}}(\mathbf{x}^*)\delta(x^*_{\mathrm{k}})\hat{\delta}\frac{1}{x^2_{\mathrm{k}}}\xi(\mathbf{x}^*)\right\}, \tag{S18f}$$

with

$$\omega_{\text{b-s}}(t, \mathbf{x}^*) = \omega_{\text{b}} - \omega_{\text{s}} \tag{S19a}$$

$$\omega_{\text{s+}/}(t, \mathbf{x}^*) = \omega_{\text{s}} + \omega_{/} \tag{S19b}$$

$$\omega_{\text{bk-sk}}(t, \mathbf{x}^*) = \omega_{\text{bk}} - \omega_{\text{sk}} \tag{S19c}$$

$$\omega_{\text{sk+/k}}(t, \mathbf{x}^*) = \omega_{\text{sk}} + \omega_{/\text{k}} \tag{S19d}$$

$$\omega_0 = 0. \tag{S19e}$$

Finally, to complete the specification of Eqs. (S15), we have

$$\theta_i = \begin{cases} 1, & \text{if } i = \text{s} \\ -1, & \text{if } i = \text{r} \end{cases} \qquad \hat{\theta}_i = \begin{cases} 0, & \text{if } i = \text{s} \\ 1, & \text{if } i = \text{r} \end{cases} \tag{S20a}$$

$$\eta_n = \begin{cases} 1, & \text{if } n = \text{k} \\ 0, & \text{if } n = 0 \end{cases} \qquad \hat{\eta}_n = \begin{cases} 0, & \text{if } n = \text{k} \\ 1, & \text{if } n = 0 \end{cases} \tag{S20b}$$

$$\hat{\delta} = \begin{cases} 1 & \text{if } c(x_\text{k}) = x_\text{k}^\gamma \\ 0 & \text{if } c(x_\text{k}) = \exp(\gamma x_\text{k}). \end{cases} \tag{S20c}$$

The analytical solutions for the candidate optimal controls given by Table A and Eqs. (S15) are functions of the candidate optimal states $\mathbf{x}^*$ and costates $\boldsymbol{\lambda}$, which we have not specified analytically. To assess if these analytical candidate optimal controls are indeed optimal, we compare them to the approximately optimal controls found numerically by GPOPS [10] (Fig. 3A,E in the main text). GPOPS uses a direct approach to obtain a numerical approximation to the solution of optimal control problems by iterating different shapes for the controls and determining which increases the value of the objective [10], rather than the indirect approach of the PMP via necessary conditions for optimality (see [11] for a comparison of direct and indirect solution approaches to optimal control problems). From the numerical approximations given by GPOPS, we obtain approximately optimal states and their costates which are part of the output given by GPOPS (Fig. A panels B-E). Feeding these numerically obtained, approximately optimal states and costates to the expressions for the analytical candidate optimal controls, we plot in Fig. B the analytical solutions for the candidate optimal controls given by Table A and Eqs. (S15). Comparison with Fig. 3A,E in the main text shows that the analytical candidate optimal controls closely follow the controls found numerically by GPOPS.

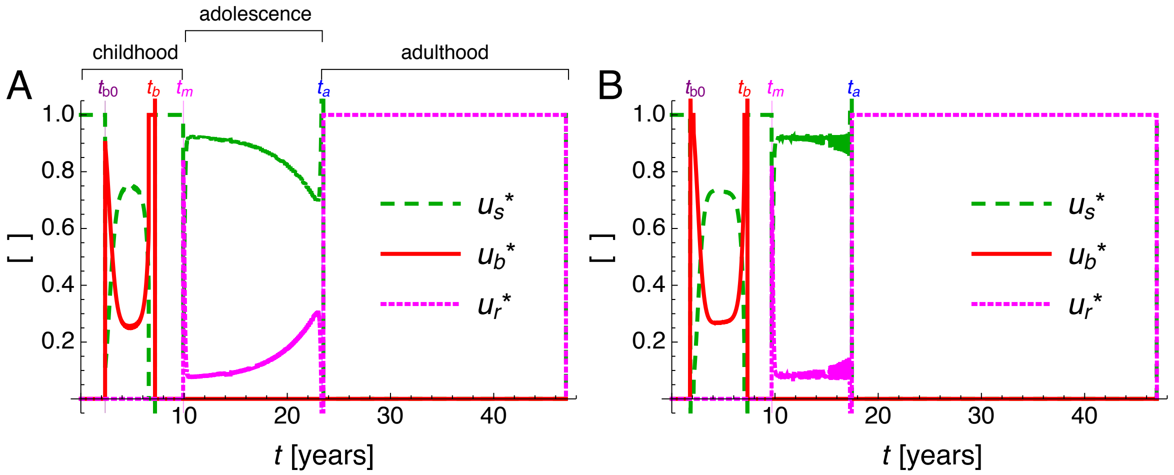

Figure B: Plots of the analytically found candidate optimal controls. (A) is for the power competence case in Fig. 3A-D in the main text. (B) is for the exponential competence case in Fig. 3E-H in the main text. In these plots, the analytically found controls are much greater than one or smaller than zero near the switching times (t_{b0}, t_{b}, t_{m}, t_{a}) between regimes (truncated here for figure clarity), possibly due to negligible numerical error in the location of the switching times. No filtering is applied to this figure.

4 Derivation of analytical results

Here we derive the expressions for the singular controls $\hat{u}_{\mathrm{b}}(t,\mathbf{x}^*,\boldsymbol{\lambda})$ and $\hat{u}_{\mathrm{s}}(t,\mathbf{x}^*,\boldsymbol{\lambda})$ given by Eqs. (S15). To do so, we make use of the well-known result, according to which singular controls can be obtained from total time derivatives of the switching functions of even but not odd order [12]; specifically, we will take the second order total time derivatives of the switching functions. During the singular arcs, either $\sigma_i = 0$ for some i or the difference $\sigma_{\mathrm{s}} - \sigma_{\mathrm{b}} = 0$, and hence their total time derivatives also equal zero during the singular arcs. Before calculating these derivatives, we obtain expressions that will be useful.

Using the overdot to denote total time derivatives, from Eqs. (S11), taking the second total time derivatives for the costates and noting that $\dot{\psi}_i = \dot{\psi}$ for $i \in \{\mathrm{b},\mathrm{r},\mathrm{s}\}$, we find

$$\ddot{\lambda}_{\mathrm{b}} = -\left(\phi\dot{\psi} + \dot{\phi}\psi_{\mathrm{b}} + \dot{\lambda}_{\mathrm{k}} b_1 B_{\mathrm{b}}\right) \tag{S21a}$$

$$\ddot{\lambda}_{\mathrm{r}} = -\left[\phi\dot{\psi} + \dot{\phi}\psi_{\mathrm{r}} - f_0\mu\exp(-\mu t)\right] \tag{S21b}$$

$$\ddot{\lambda}_{\mathrm{s}} = -\left(\phi\dot{\psi} + \dot{\phi}\psi_{\mathrm{s}}\right) \tag{S21c}$$

$$\ddot{\lambda}_{\mathrm{k}} = -\left(\phi\dot{\psi}_{\mathrm{k}} + \dot{\phi}\psi_{\mathrm{k}} - \dot{\lambda}_{\mathrm{k}} b_2\right). \tag{S21d}$$

Thsu, we need the total time derivative of ψ_i, $\dot{\psi}_i$.

4.1 Calculation of $\dot{\psi}_i$

From Eqs. (S13) we have that ψ_i is both a direct and an indirect function of time through the state variables; that is, $\psi_i(t, x_{\mathrm{b}}(t), x_{\mathrm{r}}(t), x_{\mathrm{s}}(t), x_{\mathrm{k}}(t))$. Thus, its total time derivative is

$$\dot{\psi}_i = \frac{\partial\psi_i}{\partial t} + \frac{\partial\psi_i}{\partial x_{\mathrm{s}}}\frac{\mathrm{d}x_{\mathrm{s}}}{\mathrm{d}t} + \frac{\partial\psi_i}{\partial x_{\mathrm{b}}}\frac{\mathrm{d}x_{\mathrm{b}}}{\mathrm{d}t} + \frac{\partial\psi_i}{\partial x_{\mathrm{r}}}\frac{\mathrm{d}x_{\mathrm{r}}}{\mathrm{d}t} + \frac{\partial\psi_i}{\partial x_{\mathrm{k}}}\frac{\mathrm{d}x_{\mathrm{k}}}{\mathrm{d}t}. \tag{S22}$$

For short, define

$$y_j = \begin{cases} t & \text{for } j = \mathrm{t} \\ x_j & \text{for } j \in \{\mathrm{b},\mathrm{r},\mathrm{s},\mathrm{k}\} \end{cases} \tag{S23a}$$

$$\Phi_i = \begin{cases} 0 & \text{for } i = \{\mathrm{b},\mathrm{r},\mathrm{s}\} \\ \frac{\gamma}{\delta} & \text{for } i = \mathrm{k} \end{cases} \tag{S23b}$$

$$\varsigma_i = \begin{cases} 1 & \text{for } i \in \{\mathrm{b},\mathrm{r},\mathrm{s}\} \\ 0 & \text{for } i = \mathrm{k}. \end{cases} \tag{S23c}$$

Then, in general from Eqs. (S13), we have that

$$\frac{\partial\psi_i}{\partial y_j} = \varsigma_i\frac{\partial\psi}{\partial y_j} + K\frac{\partial}{\partial y_j}\left[x_{\mathrm{B}}^{\beta} e(1-e)\Phi_i\right]. \tag{S24}$$

for $i \in \{\mathrm{b},\mathrm{r},\mathrm{s},\mathrm{k}\}$ and for $j \in \{\mathrm{t},\mathrm{b},\mathrm{r},\mathrm{s},\mathrm{k}\}$. From Eq. (S13d), we have that

$$\frac{\partial\psi}{\partial y_j} = K\beta\left[e(\beta-1)x_{\mathrm{B}}^{\beta-2}\frac{\partial x_{\mathrm{B}}}{\partial y_j} + x_{\mathrm{B}}^{\beta-1}\frac{\partial e}{\partial y_j}\right], \tag{S25}$$

while the rightmost derivative in Eq. (S24) is

$$\beta x_{\mathrm{B}}^{\beta-1}\frac{\partial x_{\mathrm{B}}}{\partial y_j}e(1-e)\Phi_i + x_{\mathrm{B}}^{\beta}\frac{\partial e}{\partial y_j}(1-e)\Phi_i - x_{\mathrm{B}}^{\beta}e\frac{\partial e}{\partial y_j}\Phi_i + x_{\mathrm{B}}^{\beta}e(1-e)\frac{\partial\Phi_i}{\partial y_j}. \tag{S26}$$

Therefore, to calculate Eq. (S24), using Eqs. (S25) and (S26), we need the following quantities

$$\frac{\partial x_{\mathrm{B}}}{\partial y_j}, \qquad \frac{\partial e}{\partial y_j}, \qquad \frac{\partial \Phi_i}{\partial y_j}. \tag{S27}$$

We have that

$$\frac{\partial x_{\mathrm{B}}}{\partial y_j} = \begin{cases} 1 & \text{for } j \in \{\mathrm{b}, \mathrm{r}, \mathrm{s}\} \\ 0 & \text{for } j \in \{\mathrm{t}, \mathrm{k}\}. \end{cases} \tag{S28}$$

Also,

$$\frac{\partial e}{\partial y_j} = e(1-e)\Phi_j \tag{S29}$$

where Φ_j is given by Eq. (S23b) for $j \in \{\mathrm{b}, \mathrm{r}, \mathrm{s}, \mathrm{k}\}$ and

$$\Phi_{\mathrm{t}} = -\varphi_{\mathrm{r}} \frac{\alpha - d}{d}. \tag{S30}$$

The latter follows since

$$\begin{aligned} \frac{\partial e}{\partial t} &= -e(1-e)\frac{\partial \ln d}{\partial t} \\ &= -e(1-e)\varphi_{\mathrm{r}} \frac{\alpha - d}{d} \end{aligned} \tag{S31a}$$

Consequently, to calculate Eq. (S24), it only remains to obtain $\partial \Phi_{\mathrm{k}} / \partial x_{\mathrm{k}}$, which is

$$\frac{\partial \Phi_{\mathrm{k}}}{\partial x_{\mathrm{k}}} = \gamma \frac{\partial}{\partial x_{\mathrm{k}}} \left(\frac{1}{\delta} \right) \tag{S32a}$$

$$= -\gamma \hat{\delta} \frac{1}{x_{\mathrm{k}}^2}. \tag{S32b}$$

We now have all the elements to write the $\dot{\psi}_i$. Using Eqs. (S22)–(S32b), (S3e), (S3f), and collecting for u_{s} and u_{b}, we obtain

$$\dot{\psi}_i(t, \mathbf{u}^*, \mathbf{x}^*) = u_{\mathrm{s}}^* \omega_{\mathrm{s}i} + u_{\mathrm{b}}^* \omega_{\mathrm{b}i} + \omega_{/i} \tag{S33a}$$

where

$$\omega_{\mathrm{ss}}(t, \mathbf{x}^*) = \frac{\psi}{x_{\mathrm{B}}} B_{\mathrm{syn}} (\beta - 1)(a_{\mathrm{s}} - a_{\mathrm{r}}) \tag{S34a}$$

$$\omega_{\mathrm{bs}}(t, \mathbf{x}^*) = \frac{\psi}{x_{\mathrm{B}}} B_{\mathrm{syn}} \left[(\beta - 1)(a_{\mathrm{b}} - a_{\mathrm{r}}) + x_{\mathrm{B}}(1-e)\frac{\gamma}{\delta} b_1 \right] \tag{S34b}$$

$$\omega_{/\mathrm{s}}(t, \mathbf{x}^*) = \frac{\psi}{x_{\mathrm{B}}} \left[B_{\mathrm{syn}} (\beta - 1) a_{\mathrm{r}} + x_{\mathrm{B}}(1-e) \left(\frac{\gamma}{\delta} \xi - \varphi_{\mathrm{r}} \frac{\alpha - d}{d} \right) \right]. \tag{S34c}$$

Since $\Phi_i = 0$ for $i \in \{\mathrm{b}, \mathrm{r}, \mathrm{s}\}$, then $\dot{\psi}_i = \dot{\psi}_{\mathrm{s}}$ for $i \in \{\mathrm{b}, \mathrm{r}, \mathrm{s}\}$. Then, we write $\omega_{ji} = \omega_j$ for $i, j \in \{\mathrm{b}, \mathrm{r}, \mathrm{s}\}$. Also,

$$\omega_{\mathrm{sk}}(t, \mathbf{x}^*) = \frac{\psi_{\mathrm{k}}}{x_{\mathrm{B}}} B_{\mathrm{syn}} \beta (a_{\mathrm{s}} - a_{\mathrm{r}}) \tag{S35a}$$

$$\omega_{\mathrm{bk}}(t, \mathbf{x}^*) = \frac{\psi_{\mathrm{k}}}{x_{\mathrm{B}}} B_{\mathrm{syn}} \left\{ \beta (a_{\mathrm{b}} - a_{\mathrm{r}}) + x_{\mathrm{B}} \left[(1-2e)\frac{\gamma}{\delta} - \delta \hat{\delta} \frac{1}{x_{\mathrm{k}}^2} \right] b_1 \right\} \tag{S35b}$$

$$\omega_{/\mathrm{k}}(t, \mathbf{x}^*) = \frac{\psi_{\mathrm{k}}}{x_{\mathrm{B}}} \left[B_{\mathrm{syn}} \beta a_{\mathrm{r}} + x_{\mathrm{B}}(1-2e) \left(\frac{\gamma}{\delta} \xi - \varphi_{\mathrm{r}} \frac{\alpha - d}{d} \right) - x_{\mathrm{B}} \delta \hat{\delta} \frac{1}{x_{\mathrm{k}}^2} \xi \right]. \tag{S35c}$$

4.2 Singular controls for regime BS: $\sigma_s > 0$, $\sigma_b > 0$, and $\sigma_s = \sigma_b$

We now obtain the singular controls for growth regime BS. The procedure is essentially the same for growth regimes BR, RS, and BRS.

For regime BS, we have the singular arc where $(u_b^*, u_s^*) = (\hat{u}_b, 1-\hat{u}_b)$ and $\sigma_s = \sigma_b$. Hence, from Eq. (S6a), during regime BS the variable ϕ in the Hamiltonian (S5) is no longer an explicit function of the controls:

$$\begin{aligned}\phi(\boldsymbol{\lambda}) &= (1-\hat{u}_b)\sigma_s + \hat{u}_b\sigma_s + a_r\lambda_r \\ &= \sigma_s + a_r\lambda_r \\ &= a_s\lambda_s. \end{aligned} \tag{S36a}$$

From Eq. (S33a), we also have the simplifications

$$\dot{\psi}(t,\mathbf{u}^*,\mathbf{x}^*) = \hat{u}_b\omega_{b-s} + \omega_{s+/} \tag{S36b}$$

$$\dot{\psi}_k = (t,\mathbf{u}^*,\mathbf{x}^*) = \hat{u}_b\omega_{bk-sk} + \omega_{sk+/k}. \tag{S36c}$$

Since $\sigma_s - \sigma_b = 0$, we have that $\ddot{\sigma}_s - \ddot{\sigma}_b = 0$, which using Eqs. (S9), (S21), and (S36) becomes

$$a_s\ddot{\lambda}_s - a_b\ddot{\lambda}_b - b_1\ddot{\lambda}_k = 0 \tag{S37a}$$

$$\Leftrightarrow \quad -a_s\left(\phi\dot{\psi} + \dot{\phi}\psi_s\right) + a_b\left(\phi\dot{\psi} + \dot{\phi}\psi_b + \dot{\lambda}_k b_1 B_b\right) + b_1\left(\phi\dot{\psi}_k + \dot{\phi}\psi_k - \dot{\lambda}_k b_2\right) = 0 \tag{S37b}$$

$$\Leftrightarrow \quad \dot{\psi}(t,\mathbf{u}^*,\mathbf{x}^*)\phi(\boldsymbol{\lambda})(a_b - a_s) + \dot{\psi}_k(t,\mathbf{u}^*,\mathbf{x}^*)\phi(\boldsymbol{\lambda})b_1 + \rho_{ks}(t,\mathbf{x}^*,\boldsymbol{\lambda}) = 0, \tag{S37c}$$

where

$$\begin{aligned}\rho_{ks}(t,\mathbf{x}^*,\boldsymbol{\lambda}) &= \dot{\lambda}_k b_1(a_b B_b - b_2) - \dot{\phi}\left(a_s\psi_s - a_b\psi_b - b_1\psi_k\right) && \text{(S38a)}\\ &= b_1 b_2\lambda_k(a_b B_b - b_2) + a_s\lambda_s\left[a_s\psi_s(a_s\psi_s - a_b\psi_b - b_1\psi_k) - b_1\psi_k(a_b B_b - b_2)\right]. && \text{(S38b)}\end{aligned}$$

(The subscripts for ρ are taken from the defining costates). Here $\dot{\lambda}_k$ during the singular arc BS is similarly not an explicit function of the controls.

In Eq. (S37c), only $\dot{\psi}$ and $\dot{\psi}_k$ are functions of $\mathbf{u}^*$. Expanding these terms in Eq. (S37c), we obtain an affine equation in the singular control $\hat{u}_b$:

$$(\hat{u}_b\omega_{b-s} + \omega_{s+/})\phi(a_b - a_s) + (\hat{u}_b\omega_{bk-sk} + \omega_{sk+/k})\phi b_1 + \rho_{sk} = 0 \tag{S39a}$$

$$-\hat{u}_b\chi^{sb}_{s(b-s)(bk-sk)}(t,\mathbf{x}^*,\boldsymbol{\lambda}) + \zeta^{sb}_{s(s+/)(sk+/k)ks}(t,\mathbf{x}^*,\boldsymbol{\lambda}) = 0, \tag{S39b}$$

where

$$\begin{aligned}\chi^{sb}_{s(b-s)(bk-sk)}(t,\mathbf{x}^*,\boldsymbol{\lambda}) &= \phi\left[\omega_{b-s}(a_s - a_b) - \omega_{bk-sk}b_1\right] \\ &= a_s\lambda_s\left[\omega_{b-s}(a_s - a_b) - \omega_{bk-sk}b_1\right] && \text{(S40a)}\\ \zeta^{sb}_{s(s+/)(sk+/k)ks}(t,\mathbf{x}^*,\boldsymbol{\lambda}) &= \rho_{ks} - \phi\left[\omega_{s+/}(a_s - a_b) - \omega_{sk+/k}b_1\right] \\ &= \rho_{ks} - a_s\lambda_s\left[\omega_{s+/}(a_s - a_b) - \omega_{sk+/k}b_1\right]. && \text{(S40b)}\end{aligned}$$

Therefore, assuming that $\chi^{sb}_{s(b-s)(bk-sk)} \neq 0$, the singular control for regime BS is

$$\hat{u}_b(t,\mathbf{x}^*,\boldsymbol{\lambda}) = \frac{\zeta^{sb}_{s(s+/)(sk+/k)ks}}{\chi^{sb}_{s(b-s)(bk-sk)}}. \tag{S41}$$

4.3 Singular controls for regime BR: $\sigma_{\mathrm{s}} < 0$ and $\sigma_{\mathrm{b}} = 0$

For regime BR, we have that $(u_{\mathrm{b}}^{*}, u_{\mathrm{s}}^{*}) = (\hat{u}_{\mathrm{b}}, 0)$. Hence, from Eq. (S6a), during regime BR the variable ϕ is no longer an explicit function of the controls:

$$\begin{aligned}\phi(\boldsymbol{\lambda}) &= 0 \times \sigma_{\mathrm{s}} + \hat{u}_{b} \times 0 + a_{\mathrm{r}}\lambda_{\mathrm{r}} \\ &= a_{\mathrm{r}}\lambda_{\mathrm{r}}. \end{aligned} \tag{S42a}$$

From Eq. (S33a), we have the simplifications

$$\dot{\psi}(t, \mathbf{u}^{*}, \mathbf{x}^{*}) = \hat{u}_{\mathrm{b}}\omega_{\mathrm{b}} + \omega_{/} \tag{S42b}$$

$$\dot{\psi}_{\mathrm{k}}(t, \mathbf{u}^{*}, \mathbf{x}^{*}) = \hat{u}_{\mathrm{b}}\omega_{\mathrm{bk}} + \omega_{/\mathrm{k}}. \tag{S42c}$$

From $\sigma_{\mathrm{b}} = 0$, we have that $\ddot{\sigma}_{\mathrm{b}} = 0$, which becomes

$$a_{\mathrm{b}}\ddot{\lambda}_{\mathrm{b}} - a_{\mathrm{r}}\ddot{\lambda}_{\mathrm{r}} + b_{1}\ddot{\lambda}_{\mathrm{k}} = 0 \tag{S43a}$$

$$\Leftrightarrow \quad -a_{\mathrm{b}}\left(\phi\dot{\psi} + \dot{\phi}\psi_{\mathrm{b}} + \dot{\lambda}_{\mathrm{k}} b_{1} B_{\mathrm{b}}\right) + a_{\mathrm{r}}\left(\phi\dot{\psi} + \dot{\phi}\psi_{\mathrm{r}} - f_{0}\mu\exp(-\mu t)\right) - b_{1}\left(\phi\dot{\psi}_{\mathrm{k}} + \dot{\phi}\psi_{\mathrm{k}} - \dot{\lambda}_{\mathrm{k}} b_{2}\right) = 0 \tag{S43b}$$

$$\Leftrightarrow \quad -\dot{\psi}(t, \mathbf{u}^{*}, \mathbf{x}^{*})\phi(\boldsymbol{\lambda})(a_{\mathrm{b}} - a_{\mathrm{r}}) - \dot{\psi}_{\mathrm{k}}(t, \mathbf{u}^{*}, \mathbf{x}^{*})\phi(\boldsymbol{\lambda}) b_{1} + \rho_{\mathrm{kr}}(t, \mathbf{x}^{*}, \boldsymbol{\lambda}) = 0, \tag{S43c}$$

where

$$\rho_{\mathrm{kr}}(t, \mathbf{x}^{*}, \boldsymbol{\lambda}) = -\dot{\lambda}_{\mathrm{k}} b_{1}\left(a_{\mathrm{b}} B_{\mathrm{b}} - b_{2}\right) + \dot{\phi}\left(a_{\mathrm{r}}\psi_{\mathrm{r}} - a_{\mathrm{b}}\psi_{\mathrm{b}} - b_{1}\psi_{\mathrm{k}}\right) - a_{\mathrm{r}} f_{0}\mu\exp(-\mu t) \tag{S44a}$$

$$\begin{aligned} &= -b_{1} b_{2}\lambda_{\mathrm{k}}\left(a_{\mathrm{b}} B_{\mathrm{b}} - b_{2}\right) - a_{\mathrm{r}}\lambda_{\mathrm{r}}\left[a_{\mathrm{r}}\psi_{\mathrm{r}}\left(a_{\mathrm{r}}\psi_{\mathrm{r}} - a_{\mathrm{b}}\psi_{\mathrm{b}} - b_{1}\psi_{\mathrm{k}}\right) - b_{1}\psi_{\mathrm{k}}\left(a_{\mathrm{b}} B_{\mathrm{b}} - b_{2}\right)\right] \\ &\quad - a_{\mathrm{r}} f_{0}\exp(-\mu t)\left[\mu + \left(a_{\mathrm{r}}\psi_{\mathrm{r}} - a_{\mathrm{b}}\psi_{\mathrm{b}} - b_{1}\psi_{\mathrm{k}}\right)\right]. \end{aligned} \tag{S44b}$$

Again, in Eq. (S43c), only $\dot{\psi}$ and $\dot{\psi}_{\mathrm{k}}$ are functions of $\mathbf{u}^{*}$. Expanding these terms in Eq. (S43c), we similarly obtain an affine equation in the singular control $\hat{u}_{\mathrm{b}}$:

$$-\left(\hat{u}_{\mathrm{b}}\omega_{\mathrm{b}} + \omega_{/}\right)\phi(a_{\mathrm{b}} - a_{\mathrm{r}}) - \left(\hat{u}_{\mathrm{b}}\omega_{\mathrm{bk}} + \omega_{/\mathrm{k}}\right)\phi b_{1} + \rho_{\mathrm{rk}} = 0 \tag{S45a}$$

$$-\hat{u}_{\mathrm{b}}\chi^{\mathrm{br}}_{\mathrm{rb(bk)}}(t, \mathbf{x}^{*}, \boldsymbol{\lambda}) + \zeta^{\mathrm{br}}_{\mathrm{r/(/k)rk}}(t, \mathbf{x}^{*}, \boldsymbol{\lambda}) = 0, \tag{S45b}$$

where

$$\begin{aligned}\chi^{\mathrm{br}}_{\mathrm{rb(bk)}}(t, \mathbf{x}^{*}, \boldsymbol{\lambda}) &= \phi\left[\omega_{\mathrm{b}}(a_{\mathrm{b}} - a_{\mathrm{r}}) + \omega_{\mathrm{bk}} b_{1}\right] \\ &= a_{\mathrm{r}}\lambda_{\mathrm{r}}\left[\omega_{\mathrm{b}}(a_{\mathrm{b}} - a_{\mathrm{r}}) + \omega_{\mathrm{bk}} b_{1}\right] \end{aligned} \tag{S46a}$$

$$\begin{aligned}\zeta^{\mathrm{br}}_{\mathrm{r/(/k)rk}}(t, \mathbf{x}^{*}, \boldsymbol{\lambda}) &= \rho_{\mathrm{kr}} - \phi\left[\omega_{/}(a_{\mathrm{b}} - a_{\mathrm{r}}) + \omega_{/\mathrm{k}} b_{1}\right] \\ &= \rho_{\mathrm{kr}} - a_{\mathrm{r}}\lambda_{\mathrm{r}}\left[\omega_{/}(a_{\mathrm{b}} - a_{\mathrm{r}}) + \omega_{/\mathrm{k}} b_{1}\right]. \end{aligned} \tag{S46b}$$

Therefore, assuming that $\chi^{\mathrm{br}}_{\mathrm{rb(bk)}} \neq 0$, the singular control for regime BR is

$$\hat{u}_{\mathrm{b}}(t, \mathbf{x}^{*}, \boldsymbol{\lambda}) = \frac{\zeta^{\mathrm{br}}_{\mathrm{r/(/k)rk}}}{\chi^{\mathrm{br}}_{\mathrm{rb(bk)}}}. \tag{S47}$$

4.4 Singular controls for regime RS: $\sigma_\mathrm{s} = 0$ and $\sigma_\mathrm{b} < 0$

For regime RS, we have that $(u_\mathrm{b}^*, u_\mathrm{s}^*) = (0, \hat{u}_\mathrm{s})$. Hence, during regime RS the variable ϕ is again no longer an explicit function of the controls:

$$\begin{aligned}\phi(\boldsymbol{\lambda}) &= \hat{u}_\mathrm{s} \times 0 + 0 \times \sigma_\mathrm{b} + a_\mathrm{r}\lambda_\mathrm{r} \\ &= a_\mathrm{r}\lambda_\mathrm{r}. \end{aligned} \tag{S48a}$$

We have the simplifications

$$\dot{\psi}(t, \mathbf{u}^*, \mathbf{x}^*) = \hat{u}_\mathrm{s}\omega_\mathrm{s} + \omega_{/} \tag{S48b}$$

$$\dot{\psi}_\mathrm{k}(t, \mathbf{u}^*, \mathbf{x}^*) = \hat{u}_\mathrm{s}\omega_\mathrm{sk} + \omega_{/\mathrm{k}}. \tag{S48c}$$

From $\sigma_\mathrm{s} = 0$, we have that $\ddot{\sigma}_\mathrm{s} = 0$, which becomes

$$a_\mathrm{s}\ddot{\lambda}_\mathrm{s} - a_\mathrm{r}\ddot{\lambda}_\mathrm{r} = 0 \tag{S49a}$$

$$\Leftrightarrow \quad -a_\mathrm{s}\left(\phi\dot{\psi} + \dot{\phi}\psi_\mathrm{s}\right) + a_\mathrm{r}\left(\phi\dot{\psi} + \dot{\phi}\psi_\mathrm{r} - f_0\mu\exp(-\mu t)\right) = 0 \tag{S49b}$$

$$\Leftrightarrow \quad -\dot{\psi}(t, \mathbf{u}, \mathbf{x})\phi(\boldsymbol{\lambda})(a_\mathrm{s} - a_\mathrm{r}) + \rho_{0\mathrm{r}}(t, \mathbf{x}^*, \boldsymbol{\lambda}) = 0, \tag{S49c}$$

where

$$\begin{aligned}\rho_{0\mathrm{r}}(t, \mathbf{x}^*, \boldsymbol{\lambda}) &= \dot{\phi}\left(a_\mathrm{r}\psi_\mathrm{r} - a_\mathrm{s}\psi_\mathrm{s}\right) - a_\mathrm{r} f_0\mu\exp(-\mu t) \\ &= -a_\mathrm{r}\lambda_\mathrm{r}\left[a_\mathrm{r}\psi_\mathrm{r}\left(a_\mathrm{r}\psi_\mathrm{r} - a_\mathrm{s}\psi_\mathrm{s}\right)\right] - a_\mathrm{r} f_0\exp(-\mu t)\left[\mu + \left(a_\mathrm{r}\psi_\mathrm{r} - a_\mathrm{s}\psi_\mathrm{s}\right)\right]. \end{aligned} \tag{S50}$$

Once again, only $\dot{\psi}$ is a function of $\mathbf{u}^*$ in Eq. (S49c). Expanding this term in Eq. (S49c), we obtain an affine equation in the singular control $\hat{u}_\mathrm{s}$:

$$-\left(\hat{u}_\mathrm{s}\omega_\mathrm{s} + \omega_{/}\right)\phi(a_\mathrm{s} - a_\mathrm{r}) + \rho_\mathrm{rs} = 0 \tag{S51a}$$

$$-\hat{u}_\mathrm{s}\chi^\mathrm{sr}_\mathrm{rs0}(t, \mathbf{x}^*, \boldsymbol{\lambda}) + \zeta^\mathrm{sr}_{\mathrm{r}/00\mathrm{r}}(t, \mathbf{x}^*, \boldsymbol{\lambda}) = 0, \tag{S51b}$$

where we define

$$\begin{aligned}\chi^\mathrm{sr}_\mathrm{rs0}(t, \mathbf{x}^*, \boldsymbol{\lambda}) &= \phi\omega_\mathrm{s}(a_\mathrm{s} - a_\mathrm{r}) \\ &= a_\mathrm{r}\lambda_\mathrm{r}\omega_\mathrm{s}(a_\mathrm{s} - a_\mathrm{r}) \end{aligned} \tag{S52a}$$

$$\begin{aligned}\zeta^\mathrm{sr}_{\mathrm{r}/00\mathrm{r}}(t, \mathbf{x}^*, \boldsymbol{\lambda}) &= \rho_{0\mathrm{r}} - \phi\omega_{/}(a_\mathrm{s} - a_\mathrm{r}) \\ &= \rho_{0\mathrm{r}} - a_\mathrm{r}\lambda_\mathrm{r}\omega_{/}(a_\mathrm{s} - a_\mathrm{r}). \end{aligned} \tag{S52b}$$

Therefore, assuming that $\chi^\mathrm{sr}_\mathrm{rs0} \neq 0$, the singular control for regime RS is

$$\hat{u}_\mathrm{s}(t, \mathbf{x}^*, \boldsymbol{\lambda}) = \frac{\zeta^\mathrm{sr}_{\mathrm{r}/00\mathrm{r}}}{\chi^\mathrm{sr}_\mathrm{rs0}}. \tag{S53}$$

4.5 Singular controls for regime BRS: $\sigma_\mathrm{s} = \sigma_\mathrm{b} = 0$

For regime BRS, we have that $(u_\mathrm{b}^*, u_\mathrm{s}^*) = (\hat{u}_\mathrm{b}, \hat{u}_\mathrm{s})$. As before, the variable ϕ is no longer an explicit function of the controls:

$$\begin{aligned}\phi(\boldsymbol{\lambda}) &= \hat{u}_\mathrm{s} \times 0 + \hat{u}_\mathrm{b} \times 0 + a_\mathrm{r}\lambda_\mathrm{r} \\ &= a_\mathrm{r}\lambda_\mathrm{r}. \end{aligned} \tag{S54a}$$

Similarly, we have the simplifications

$$\dot{\psi}(t,\mathbf{u}^*,\mathbf{x}^*) = \hat{u}_\mathrm{s}\omega_\mathrm{s} + \hat{u}_\mathrm{b}\omega_\mathrm{b} + \omega_{/} \tag{S54b}$$

$$\dot{\psi}_\mathrm{k}(t,\mathbf{u}^*,\mathbf{x}^*) = \hat{u}_\mathrm{s}\omega_\mathrm{sk} + \hat{u}_\mathrm{b}\omega_\mathrm{bk} + \omega_{/\mathrm{k}}. \tag{S54c}$$

From $\sigma_\mathrm{s} = 0$, we have that $\ddot{\sigma}_\mathrm{s} = 0$, which is

$$a_\mathrm{s}\ddot{\lambda}_\mathrm{s} - a_\mathrm{r}\ddot{\lambda}_\mathrm{r} = 0 \tag{S55a}$$

$$\Leftrightarrow \quad -a_\mathrm{s}\left(\phi\dot{\psi} + \dot{\phi}\psi_\mathrm{s}\right) + a_\mathrm{r}\left[\phi\dot{\psi} + \dot{\phi}\psi_\mathrm{r} - f_0\mu\exp(-\mu t)\right] = 0 \tag{S55b}$$

$$\Leftrightarrow \quad \dot{\psi}(t,\mathbf{u}^*,\mathbf{x}^*)\phi(\boldsymbol{\lambda})(a_\mathrm{r} - a_\mathrm{s}) + \rho_{0\mathrm{r}}(t,\mathbf{x}^*,\boldsymbol{\lambda}) = 0, \tag{S55c}$$

where as before

$$\begin{aligned}\rho_{0\mathrm{r}}(t,\mathbf{x}^*,\boldsymbol{\lambda}) &= \dot{\phi}\left(a_\mathrm{r}\psi_r - a_\mathrm{s}\psi_\mathrm{s}\right) - a_\mathrm{r}f_0\mu\exp(-\mu t)\\ &= -a_\mathrm{r}\lambda_\mathrm{r}\left[a_\mathrm{r}\psi_\mathrm{r}\left(a_\mathrm{r}\psi_\mathrm{r} - a_\mathrm{s}\psi_\mathrm{s}\right)\right] - a_\mathrm{r}f_0\exp(-\mu t)\left[\mu + \left(a_\mathrm{r}\psi_\mathrm{r} - a_\mathrm{s}\psi_\mathrm{s}\right)\right].\end{aligned} \tag{S56}$$

Expanding $\dot{\psi}$ in Eq. (S55c), we obtain an affine equation in the two controls $\hat{u}_\mathrm{s}$ and $\hat{u}_\mathrm{b}$:

$$\left(\hat{u}_\mathrm{s}\omega_\mathrm{s} + \hat{u}_\mathrm{b}\omega_\mathrm{b} + \omega_{/}\right)\phi(a_\mathrm{r} - a_\mathrm{s}) + \rho_\mathrm{rs} = 0 \tag{S57a}$$

$$-\hat{u}_\mathrm{s}\chi^\mathrm{sr}_\mathrm{rs0}(t,\mathbf{x}^*,\boldsymbol{\lambda}) - \hat{u}_\mathrm{b}\chi^\mathrm{sr}_\mathrm{rb0}(t,\mathbf{x}^*,\boldsymbol{\lambda}) + \zeta^\mathrm{rs}_{\mathrm{r}/00\mathrm{r}}(t,\mathbf{x}^*,\boldsymbol{\lambda}) = 0, \tag{S57b}$$

where

$$\begin{aligned}\chi^\mathrm{sr}_\mathrm{rs0}(t,\mathbf{x}^*,\boldsymbol{\lambda}) &= -\phi\omega_\mathrm{s}(a_\mathrm{r} - a_\mathrm{s})\\ &= a_\mathrm{r}\lambda_\mathrm{r}\omega_\mathrm{s}(a_\mathrm{s} - a_\mathrm{r})\end{aligned} \tag{S58a}$$

$$\begin{aligned}\chi^\mathrm{sr}_\mathrm{rb0}(t,\mathbf{x}^*,\boldsymbol{\lambda}) &= -\phi\omega_\mathrm{b}(a_\mathrm{r} - a_\mathrm{s})\\ &= a_\mathrm{r}\lambda_\mathrm{r}\omega_\mathrm{b}(a_\mathrm{s} - a_\mathrm{r})\end{aligned} \tag{S58b}$$

$$\begin{aligned}\zeta^\mathrm{rs}_{\mathrm{r}/00\mathrm{r}}(t,\mathbf{x}^*,\boldsymbol{\lambda}) &= \rho_{0\mathrm{r}} + \phi\omega_{/}(a_\mathrm{r} - a_\mathrm{s})\\ &= \rho_{0\mathrm{r}} - a_\mathrm{r}\lambda_\mathrm{r}\omega_{/}(a_\mathrm{s} - a_\mathrm{r}).\end{aligned} \tag{S58c}$$

Now, from $\sigma_\mathrm{b} = 0$, we have that $\ddot{\sigma}_\mathrm{b} = 0$, which is

$$a_\mathrm{b}\lambda_\mathrm{b} - a_\mathrm{r}\lambda_\mathrm{r} + b_1\lambda_\mathrm{k} = 0 \tag{S59a}$$

$$\Leftrightarrow \quad -a_\mathrm{b}\left(\phi\dot{\psi} + \dot{\phi}\psi_\mathrm{b} + \dot{\lambda}_\mathrm{k}b_1B_\mathrm{b}\right) + a_\mathrm{r}\left[\phi\dot{\psi} + \dot{\phi}\psi_\mathrm{r} - \mu\exp(-\mu t)\right] - b_1\left(\phi\dot{\psi}_\mathrm{k} + \dot{\phi}\psi_\mathrm{k} - \dot{\lambda}_\mathrm{k}b_2\right) = 0 \tag{S59b}$$

$$\Leftrightarrow \quad -\dot{\psi}(t,\mathbf{u}^*,\mathbf{x}^*)\phi(\boldsymbol{\lambda})(a_\mathrm{b} - a_\mathrm{r}) - \dot{\psi}_\mathrm{k}(t,\mathbf{u}^*,\mathbf{x}^*)\phi(\boldsymbol{\lambda})b_1 + \rho_\mathrm{kr}(t,\mathbf{x}^*,\boldsymbol{\lambda}) = 0, \tag{S59c}$$

where as before

$$\rho_\mathrm{kr}(t,\mathbf{x}^*,\boldsymbol{\lambda}) = -\dot{\lambda}_\mathrm{k}b_1\left(a_\mathrm{b}B_\mathrm{b} - b_2\right) + \dot{\phi}\left(a_\mathrm{r}\psi_r - a_\mathrm{b}\psi_\mathrm{b} - b_1\psi_\mathrm{k}\right) - a_\mathrm{r}f_0\mu\exp(-\mu t) \tag{S60a}$$

$$\begin{aligned}&= -b_1b_2\lambda_\mathrm{k}\left(a_\mathrm{b}B_\mathrm{b} - b_2\right) - a_\mathrm{r}\lambda_\mathrm{r}\left[a_\mathrm{r}\psi_\mathrm{r}\left(a_\mathrm{r}\psi_\mathrm{r} - a_\mathrm{b}\psi_\mathrm{b} - b_1\psi_\mathrm{k}\right) - b_1\psi_\mathrm{k}\left(a_\mathrm{b}B_\mathrm{b} - b_2\right)\right]\\ &\quad - a_\mathrm{r}f_0\exp(-\mu t)\left[\mu + \left(a_\mathrm{r}\psi_\mathrm{r} - a_\mathrm{b}\psi_\mathrm{b} - b_1\psi_\mathrm{k}\right)\right].\end{aligned} \tag{S60b}$$

Expanding $\dot{\psi}$ and $\dot{\psi}_\mathrm{k}$ in Eq. (S59c), we obtain another affine equation in the two controls $\hat{u}_\mathrm{s}$ and $\hat{u}_\mathrm{b}$:

$$-\left(\hat{u}_\mathrm{s}\omega_\mathrm{s} + \hat{u}_\mathrm{b}\omega_\mathrm{b} + \omega_{/}\right)\phi(a_\mathrm{b} - a_\mathrm{r}) - \left(\hat{u}_\mathrm{s}\omega_\mathrm{sk} + \hat{u}_\mathrm{b}\omega_\mathrm{bk} + \omega_{/\mathrm{k}}\right)\phi b_1 + \rho_\mathrm{rk} = 0 \tag{S61a}$$

$$-\hat{u}_\mathrm{s}\chi^\mathrm{br}_\mathrm{rs(sk)}(t,\mathbf{x}^*,\boldsymbol{\lambda}) - \hat{u}_\mathrm{b}\chi^\mathrm{br}_\mathrm{rb(bk)}(t,\mathbf{x}^*,\boldsymbol{\lambda}) + \zeta^\mathrm{br}_{\mathrm{r}/(/\mathrm{k})\mathrm{kr}}(t,\mathbf{x}^*,\boldsymbol{\lambda}) = 0, \tag{S61b}$$

where

$$\chi^{\rm br}_{\rm rs(sk)}(t,\mathbf{x}^*,\boldsymbol{\lambda}) = \phi\,[\omega_{\rm s}(a_{\rm b}-a_{\rm r})+\omega_{\rm sk}b_1]$$
$$= a_{\rm r}\lambda_{\rm r}\,[\omega_{\rm s}(a_{\rm b}-a_{\rm r})+\omega_{\rm sk}b_1] \tag{S62a}$$
$$\chi^{\rm br}_{\rm rb(bk)}(t,\mathbf{x}^*,\boldsymbol{\lambda}) = \phi\,[\omega_{\rm b}(a_{\rm b}-a_{\rm r})+\omega_{\rm bk}b_1]$$
$$= a_{\rm r}\lambda_{\rm r}\,[\omega_{\rm b}(a_{\rm b}-a_{\rm r})+\omega_{\rm bk}b_1] \tag{S62b}$$
$$\zeta^{\rm br}_{\rm r/(/k)kr}(t,\mathbf{x}^*,\boldsymbol{\lambda}) = \rho_{\rm kr}-\phi\,[\omega_{/}(a_{\rm b}-a_{\rm r})+\omega_{/\rm k}b_1]$$
$$= \rho_{\rm kr}-a_{\rm r}\lambda_{\rm r}\,[\omega_{/}(a_{\rm b}-a_{\rm r})+\omega_{/\rm k}b_1]\,. \tag{S62c}$$

Therefore, solving for $\hat{u}_{\rm s}$ and $\hat{u}_{\rm b}$ in Eqs. (S57b) and (S61b) and assuming that $D \neq 0$, the singular controls for regime BRS are

$$\hat{u}_{\rm s}(t,\mathbf{x}^*,\boldsymbol{\lambda}) = \frac{1}{D}\left[\zeta^{\rm rs}_{\rm r/00r}\chi^{\rm br}_{\rm rb(bk)}-\chi^{\rm sr}_{\rm rb0}\zeta^{\rm br}_{\rm r/(/k)kr}\right] \tag{S63a}$$
$$\hat{u}_{\rm b}(t,\mathbf{x}^*,\boldsymbol{\lambda}) = \frac{1}{D}\left[\chi^{\rm sr}_{\rm sr0}\zeta^{\rm br}_{\rm r/(/k)kr}-\zeta^{\rm rs}_{\rm r/00r}\chi^{\rm br}_{\rm rs(sk)}\right] \tag{S63b}$$
$$D = \chi^{\rm sr}_{\rm rs0}\chi^{\rm br}_{\rm rb(bk)}-\chi^{\rm sr}_{\rm rb0}\chi^{\rm br}_{\rm rs(sk)}. \tag{S63c}$$

5 Parameter values

Here we summarize the values of the 22 parameters used in the numerical approximations. From these, 13 parameters are estimated as described in section 6 and they refer to newborn mass, tissue metabolism, and demography (Table B). The estimates of E_i are less accurate than those of B_i for $i \in \{\mathrm{b}, \mathrm{s}, \mathrm{r}\}$ as the former require stronger assumptions given the available data (see [13]). Since the parameter f_0 only displaces the objective vertically and thus has no effect on the solution, we choose its value to scale the objective J and facilitate the numerical procedure (Table B). The remaining 8 parameters refer to skill metabolism, energy extraction, and maternal care, for which we use benchmark values that produce body and brain mass that closely approach ontogenetic modern human data. Hence, we use different benchmark values with either power (Table C) or exponential (Table D) competence.

Newborn mass		Tissue metabolism				Demography	
		K	$132.7281 \frac{\mathrm{MJ}}{\mathrm{y}} \mathrm{kg}^{-\beta}$	β	0.7378		
$x_\mathrm{s}(0)$	2.0628 kg	B_s	$29.6891 \frac{\mathrm{MJ}}{\mathrm{y} \times \mathrm{kg}}$	E_s	$12.4594 \frac{\mathrm{MJ}}{\mathrm{kg}}$	f_0	$10 \frac{\#\mathrm{offspring}}{\mathrm{kg} \times \mathrm{y}}$
$x_\mathrm{b}(0)$	0.3372 kg	B_b	$313.0962 \frac{\mathrm{MJ}}{\mathrm{y} \times \mathrm{kg}}$	E_b	$123.7584 \frac{\mathrm{MJ}}{\mathrm{kg}}$	μ	$0.034 \frac{1}{\mathrm{y}}$
$x_\mathrm{r}(0)$	0 kg	B_r	$2697.1179 \frac{\mathrm{MJ}}{\mathrm{y} \times \mathrm{kg}}$	E_r	$190.8196 \frac{\mathrm{MJ}}{\mathrm{kg}}$	T	47 y

Table B: Estimated parameter values and f_0, which is set to an arbitrary value. Note that B_i and E_i are per mass unit. So, with an estimated adult brain and reproductive mass of ≈ 1.31 kg and ≈ 3 g (§6.1), respectively, the estimated B_b and B_r yield an estimated adult brain and reproductive metabolic rates of ≈ 410 MJ/y and ≈ 8 MJ/y, respectively.

For power competence:

Skill metabolism		Energy extraction		Maternal care	
s_k	0.5	α	1 skill$^\gamma$	φ_0	0.6
B_k	$36 \frac{\mathrm{MJ}}{\mathrm{y} \times \mathrm{skill}}$	γ	1.4	φ_r	$0.2 \frac{1}{\mathrm{y}}$
E_k	$370 \frac{\mathrm{MJ}}{\mathrm{skill}}$	$x_\mathrm{k}(0)$	1 skill		

Table C: Benchmark parameter values with power competence. The value used for φ_r yields maternal care for ≈ 20 years, as observed in forager-horticulturalists [14]. For short, we occasionally write "skill unit" as "skill".

For exponential competence:

Skill metabolism		Energy extraction		Maternal care	
s_k	0.5	α	1.15	φ_0	0.8
B_k	$50 \frac{\mathrm{MJ}}{\mathrm{y} \times \mathrm{skill}}$	γ	0.6 skill^{-1}	φ_r	$0.2 \frac{1}{\mathrm{y}}$
E_k	$250 \frac{\mathrm{MJ}}{\mathrm{skill}}$	$x_\mathrm{k}(0)$	0 skill		

Table D: Benchmark parameter values with exponential competence.

6 Estimation of parameter values

Here we describe how we obtained the parameter values in Table B. We use ontogenetic data for modern human females published in Table S2 of [15]. We denote the observed mass of tissue i at age t as $X_i(t)$ and their sum as $X_B(t)$. Thus, we set $x_s(0) = X_s(0) = 2.0628$ kg and $x_b(0) = X_b(0) = 0.3372$ kg [15]. We take reproductive cells as referring to preovulatory ovarian follicle cells, and thus set $x_r(0) = 0$ kg. We also denote by τ_a the observed age at adulthood. Hence, $X_B(\tau_a) = 51.1$ kg and $X_b(\tau_a) = 1.31$ kg [15]. We also have that $B_{rest}(\tau_a) = 1243.4$ kcal/day × 4184 J/kcal × 365 d/y = 1898.8707 MJ/y [15].

As stated in the main text, we assume that B_i and E_i are constant with respect to age. However, they are likely to vary with age in real systems. So, we seek to estimate these parameters' value around the ages where the parameter is expected to have the strongest effects on growth dynamics. From the shape of the ontogenetic dynamic equations (Eqs. 24), the growth dynamics are more likely to be driven by maintenance costs B_i later in life and by acquisition costs E_i at points in life where the tissue is growing the fastest (see [16]). So, to obtain the values of B_i and E_i at the ages that are presumably most affected by them, we estimate B_i from data for adults, E_b from data for newborns, E_r from data for fifteen year old females, and E_s for newborns.

6.1 Values for B_i for $i \in \{b, r, s\}$

B_b: Let $c_1(t)$ be the ratio of glucose uptake by the brain per unit time at age t divided by the resting metabolic rate at that age. Let $c_2(t)$ be the fraction of brain glucose metabolism that is oxidative. Then, the empirically estimated brain metabolic rate at age t is the product $B_{rest}(t)c_1(t)c_2(t)$. $c_1(t)$ is obtained from Table S2 of [15] and rough estimates of $c_2(t)$ are obtained from [17]. For adults they are $c_1(\tau_a) = 0.24$ and $c_2(\tau_a) = 0.9$ [15, 17]. Hence, we let $B_b = B_{rest}(\tau_a)c_1(\tau_a)c_2(\tau_a)/X_b(\tau_a) = 313.0962$ MJ/kg/y.

B_r: We are unable to find reports of the metabolic rate of preovulatory follicles. Thus, we use the metabolic rate of a human oocyte as a proxy. The oxygen consumption by a human oocyte is estimated to be 0.53×10^{-9} l O_2/h/oocyte [18]. Oxygen consumption can be transformed into power units by multiplying by 20.1 kJ/l O_2 [19]. The mass of a mouse oocyte is 34.6 ng [20]. Assuming that mouse and human oocytes are of similar mass, then $B_r = 0.53 \times 10^{-9} \frac{1\,O_2}{\text{h}\times\text{oocyte}} \times 20.1 \frac{\text{kJ}}{1\,O_2} \times \frac{1 \text{ oocyte}}{34.6 \text{ ng}} \times \frac{24 \text{ h}}{1 \text{ d}} \times \frac{365 \text{ d}}{1 \text{ y}} \times \frac{10^9 \text{ ng}}{1 \text{ g}} \times \frac{1000 \text{ g}}{1 \text{ kg}} \times \frac{1 \text{ MJ}}{1000 \text{ kJ}} = 2697.1179$ MJ/kg/year.

B_s: Adult human females have on average about 2 preovulatory follicles at any given age [21]. A preovulatory follicle has an average diameter of 21.1 mm [22]. Approximating the follicle dry mass by the dry mass of a spherical cell with such diameter and water content of 60%, then the adult mass of reproductive tissue is $X_r(\tau_a) = 2 \text{ follicles} \times \frac{4}{3}\pi\left(\frac{21.1 \text{ mm}}{2}\right)^3 \times \frac{1 \text{ kg } H_2O}{10^6 \text{ mm}^3 H_2O} \times \frac{0.4 \text{ kg dry mass}}{1 \text{ kg } H_2O} = 3.9349 \times 10^{-3}$ kg. Hence, $X_s(\tau_a) = X_B(\tau_a) - X_b(\tau_a) - X_r(\tau_a) = 49.7861$ kg.

Since at human adulthood there is no growth, it must be the case that $B_{rest}(\tau_a) = B_{maint}(\tau_a) = \sum_{i\in\{b,r,s\}} X_i(\tau_a)B_i$. Because we have that $B_{rest}(\tau_a) = 1898.8707$ MJ/y, it follows that $B_s = [B_{rest}(\tau_a) - B_b X_b(\tau_a) - B_r X_r(\tau_a)]/X_s(\tau_a) = 29.6891$ MJ/kg/y.

6.2 Values for E_i for $i \in \{\mathrm{b}, \mathrm{r}, \mathrm{s}\}$

E_b : We have that brain metabolic rate is $B_{\mathrm{rest,b}}(t) = X_\mathrm{b}(t)B_\mathrm{b} + \dot{X}_\mathrm{b}(t)E_\mathrm{b}$. Hence, E_b should satisfy $E_\mathrm{b} = [B_{\mathrm{rest,b}}(0) - X_\mathrm{b}(0)B_\mathrm{b}]/\dot{X}_\mathrm{b}(0)$. We have also that $B_{\mathrm{rest,b}}(0) = B_\mathrm{rest}(0)c_1(0)c_2(0)$ and that $B_\mathrm{rest}(0) = 166.6132$ MJ/y [15], $c_1(0) = 0.598$ [15], and $c_2(0) \approx 0.9$ [17]. From the data in [15], we estimate $\dot{X}_\mathrm{b}(0) = 0.7246$ kg/y. Using these values and the estimated B_b, the resulting $[B_{\mathrm{rest,b}}(0) - X_\mathrm{b}(0)B_\mathrm{b}]/\dot{X}_\mathrm{b}(0)$ is negative. However, this may be a consequence of B_b being estimated for adults while the other quantities are for newborns. Therefore, we instead assume that at birth most brain metabolic rate is due to brain growth (i.e., $\dot{X}_\mathrm{b}(0)E_\mathrm{b} \gg X_\mathrm{b}(0)B_\mathrm{b}$, either because at birth brain growth rate is substantial relative to brain mass or because *at birth* mass-specific brain maintenance is substantially small relative to production). So $B_{\mathrm{rest,b}}(0) \approx \dot{X}_\mathrm{b}(0)E_\mathrm{b}$. Then, we let $E_\mathrm{b} = B_{\mathrm{rest,b}}(0)/\dot{X}_\mathrm{b}(0) = 123.7584$ MJ/kg.

E_r : We have that $B_\mathrm{syn}(t) = \sum_{i\in\{\mathrm{b,r,s}\}} \dot{X}_i(t)E_i$. We assume that shortly before adulthood, specifically at fifteen years of age, most growth is reproductive. So assuming $\dot{X}_\mathrm{r}(15) \neq 0$ while $\dot{X}_{i\neq\mathrm{r}}(15) \approx 0$, we have that

$$E_\mathrm{r} = \frac{B_\mathrm{rest}(15) - B_\mathrm{maint}(15)}{\dot{X}_\mathrm{r}(15)} \tag{S64a}$$

$$= \frac{B_\mathrm{rest}(15) - BX_\mathrm{B}(15)}{\dot{X}_\mathrm{B}(15)}, \tag{S64b}$$

where the mass-specific resting metabolic rate is $B = B_\mathrm{rest}(\tau_\mathrm{a})/X_\mathrm{B}(\tau_\mathrm{a}) = 37.1599$ MJ/y/kg. We also have that $B_\mathrm{rest}(15) = 1328.3\frac{\mathrm{kcal}}{\mathrm{d}} \times \frac{4184\,\mathrm{J}}{1\,\mathrm{kcal}} \times \frac{365\,\mathrm{d}}{1\,\mathrm{y}} = 2028.5266$ MJ/y, $X_\mathrm{B}(15) = 47.4$ kg, and $\dot{X}_\mathrm{B}(15) = 1.4$ kg/y [15]. Then, $E_\mathrm{r} = 190.8196$ MJ/kg.

E_s : Again, we have that $B_\mathrm{syn}(t) = \sum_{i\in\{\mathrm{b,r,s}\}} \dot{X}_i(t)E_i$. Assuming that there is no reproductive growth at birth, then $\dot{X}_\mathrm{r}(0) = 0$ and so

$$E_\mathrm{s} = \frac{B_\mathrm{rest}(0) - B_\mathrm{maint}(0) - \dot{X}_\mathrm{b}(0)E_\mathrm{b}}{\dot{X}_\mathrm{s}(0)} \tag{S65a}$$

$$\approx \frac{B_\mathrm{rest}(0) - \dot{X}_\mathrm{b}(0)E_\mathrm{b}}{\dot{X}_\mathrm{s}(0)}, \tag{S65b}$$

assuming that at birth most resting metabolic rate is due to growth so $B_\mathrm{rest}(0) - B_\mathrm{maint}(0) \approx B_\mathrm{rest}(0)$. We have that $B_\mathrm{rest}(0) = 109.1\frac{\mathrm{kcal}}{\mathrm{d}} \times \frac{4184\,\mathrm{J}}{1\,\mathrm{kcal}} \times \frac{365\,\mathrm{d}}{1\,\mathrm{y}} = 166.6132$ MJ/y and $\dot{X}_\mathrm{B}(0) = 6.9$ kg/y [15]. Since $\dot{X}_\mathrm{s}(0) = \dot{X}_\mathrm{B}(0) - \dot{X}_\mathrm{b}(0)$, then $E_\mathrm{s} = 12.4594$ MJ/kg.

6.3 Values for K and β

Using the ontogenetic (averaged) data in Table S2 of [15], where resting metabolic rate is measured in well fed individuals, we find that $B_\mathrm{rest}(t) = KX_\mathrm{B}^\beta(t)$ with $K = 132.7281\frac{\mathrm{MJ}}{\mathrm{y}}\mathrm{kg}^{-\beta}$ and $\beta = 0.7378$ ($R^2 = 0.92$) (Fig. C).

6.4 Values for f_0, μ, and T

The constant f_0 only multiplies the objective J and thus has not effect on the solution of the optimal control problem, but we use it to re-scale the objective to facilitate convergence of the GPOPS algorithm. We thus set it to $f_0 = 10\frac{\#\,\mathrm{offspring}}{\mathrm{kg}\times\mathrm{y}}$.

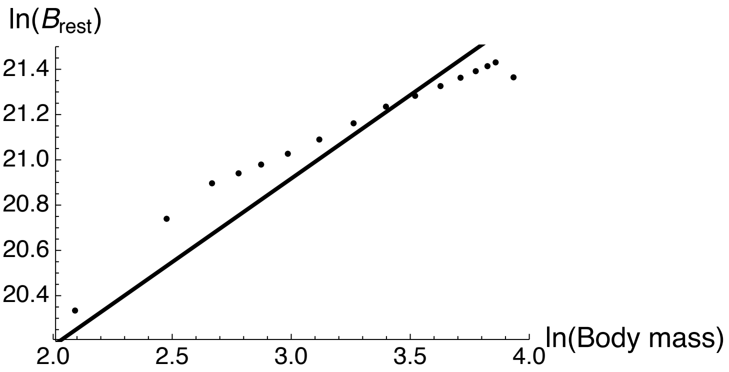

Figure C: Power law approximation of resting metabolic rate with respect to body mass. Dots are ontogenetic values of resting metabolic rate vs body mass in modern humans in a log-log scale [15]. The line is the linear least square regression yielding $K = 132.7281\,\frac{\mathrm{MJ}}{\mathrm{y}}\,\mathrm{kg}^{-\beta}$ and $\beta = 0.7378$ ($R^2 = 0.92$).

For traditional hunter-gatherers, the average life expectancy at birth is between 21 and 37 years [23]. The mid-range life expectancy is thus 29 years. With a constant mortality rate, life expectancy is $1/\mu$. We thus let $\mu = \frac{1}{29\,\mathrm{y}} = 0.034\,\frac{1}{\mathrm{y}}$.

For Hadza and Gainj hunter-gatherers, the average age at menopause is about 47 years [24]. So, we let $T = 47$ years.

7 Numerical implementation

GPOPS 2.0 was used to obtain the numerical approximations of the optimal solutions [10]. GPOPS is based on a pseudospectral method which converts the optimal control problem into a finite-dimensional nonlinear program. GPOPS adapts the underlying time mesh (partitions of the optimization horizon, or time interval) until the error tolerance is met or until the maximum number of iterations is reached. We discarded solutions that did not meet the error tolerance or where the solver failed [e.g., stalled in a zero objective (i.e., a minimum)].

The GPOPS setup used was:

```
mesh.method = 'hp-PattersonRao';

mesh.tolerance = 1e-6;

mesh.maxiterations = 45;

setup.method = 'RPM-Integration';
```

To facilitate convergence of dynamic optimization algorithms, non-negative state variables should be scaled so that they fall roughly between 0 and 1. So, we ran GPOPS with mass and skill units rescalled to Mg (megagrams) and kilo-skill units.

For Fig. 3 in the main text, the initial guess used was

$$x_{\mathrm{b}}(0) = x_{\mathrm{b}0} \quad x_{\mathrm{b}}(T) = 1.3\ \mathrm{kg} \tag{S66a}$$

$$x_{\mathrm{r}}(0) = x_{\mathrm{r}0} \quad x_{\mathrm{r}}(T) = 0.2\ \mathrm{kg} \tag{S66b}$$

$$x_{\mathrm{s}}(0) = x_{\mathrm{s}0} \quad x_{\mathrm{s}}(T) = 50\ \mathrm{kg} \tag{S66c}$$

$$x_{\mathrm{k}}(0) = x_{\mathrm{k}0} \quad x_{\mathrm{k}}(T) = 40\ \text{skill unit} \tag{S66d}$$

$$u_{\mathrm{b}}(0) = 0.2 \quad u_{\mathrm{b}}(T) = 0 \tag{S66e}$$

$$u_{\mathrm{s}}(0) = 0.8 \quad u_{\mathrm{s}}(T) = 0. \tag{S66f}$$

For parameter sweeps (e.g., Fig. 6 in the main text), we let each parameter $p \in \{\alpha, \ldots, s_{\mathrm{k}}\}$ take values p_i for $i \in \{1, \ldots, n\}$. For $i = 1$, the initial guess was given by Eqs. (S66), and for $i > 1$ the initial guess was the solution found with $i - 1$. This often facilitates convergence. The mesh partitioning algorithm of GPOPS can lead to numerical instability on Mac OS computers, so we ran GPOPS in Windows as recommended by GPOPS developers. GPOPS is also stable in Linux.

8 Supplementary results

8.1 Results without image filtering

The solution of an optimal control problem during singular arcs is numerically challenging, and so GPOPS may yield numerical jitter during singular arcs. For clarity in the figures, we filter all figures involving the control strategies by plotting one every ten points rather than all the points. To illustrate the effect of such filtering, Fig. 3 in the main text is shown in Fig. D without such filtering. The effect of filtering can be seen only in the singular arcs (when more than one tissue grow simultaneously) (Fig. D panels A,E).

8.2 Tests of assumptions

8.2.1 Effect of fecundity approximation

Fig. E shows the obtained uninvadable growth strategy and resulting growth patterns using Eq. (29) with $B_o \approx 0$ but without neglecting $\dot{x}_r(t)E_r$; that is, letting fecundity be

$$f(t) = \frac{s_o[x_r(t)B_r + \dot{x}_r(t)E_r]}{E_o}. \tag{S67}$$

To calculate Eq. (S67), it is not necessary to estimate the value of the new parameters for s_o and E_o, but only the value of the ratio $s_o/E_o = f_0/B_r$ which was already estimated.

The error in the approximated fecundity (Eq. 30) is shown in Fig. F. The predicted switching times as well as the predicted adult body and brain mass with fecundity given by Eq. (S67) are in Table E. In this case, the predicted brain and body mass are identical with those obtained with the approximated fecundity (compare Table E with Table 1 in the main text).

Table E: Predictions for life history timing with fecundity given by Eq. (S67). The values are the results as in Table 1 in the main text but with fecundity given by Eq. (S67). Values with a ** differ (slightly) from those in Table 1 in the main text.

		Predicted with		Observed in*
		PC	EC	*H. sapiens*
Age at:	Maturity, t_m [y]	9.94	9.64**	7−13
	Adulthood, t_a [y]	23.45**	17.33	≈17
	Brain growth onset, t_{b0} [y]	2.35**	1.76**	0
	Brain growth arrest, t_b [y]	7.19	7.29**	≈17
	Adult body mass, [kg]	53.19	67.79	51.1
	Adult brain mass, [kg]	1.02	1.53	1.31
	EQ†, [ ]	4.43	5.52	5.87

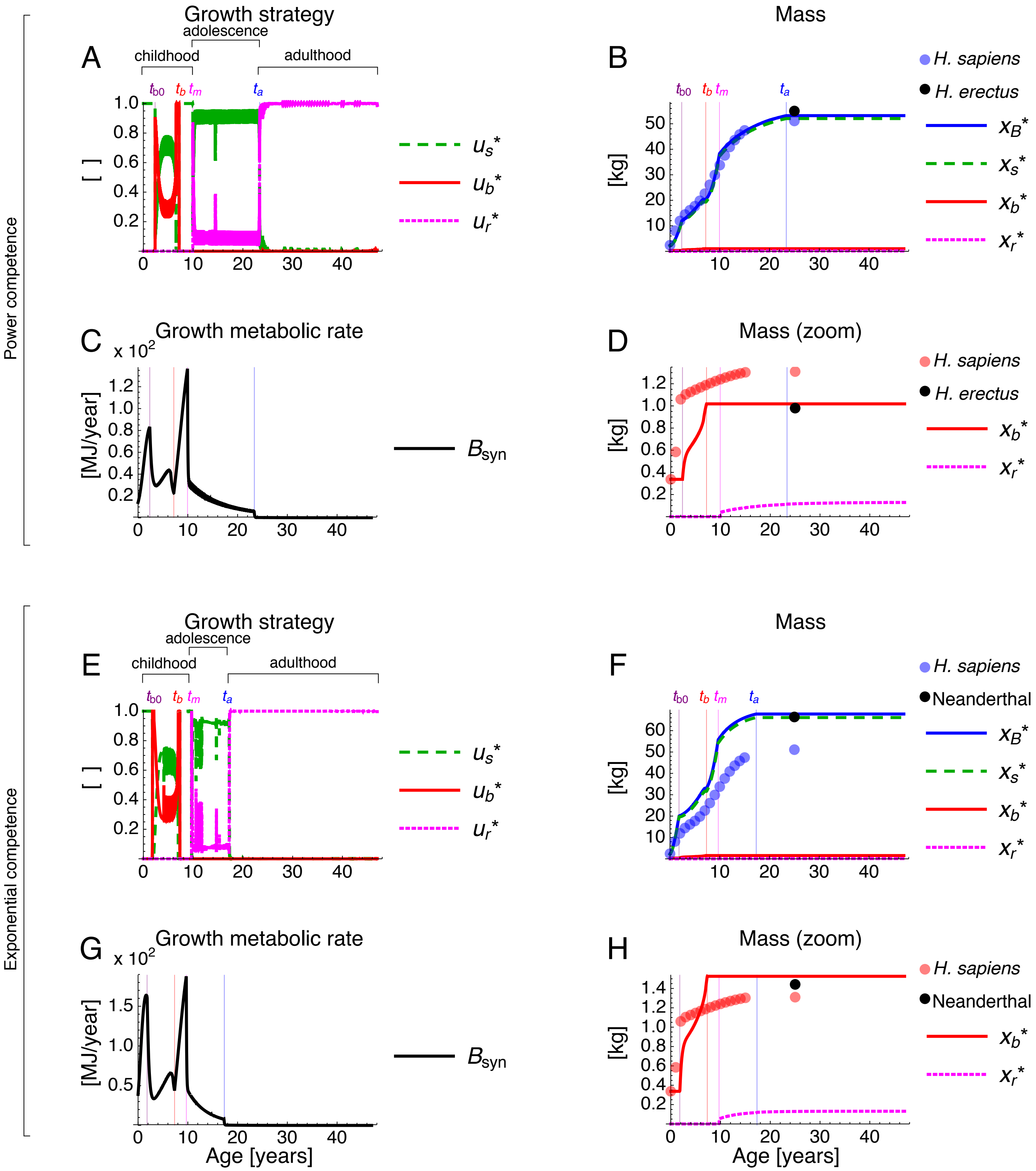

Figure D: Same as Fig. 3 in the main text without image filtering. Jitter in the controls indicates that the optimal control problem is computationally challenging for GPOPS (this applies to all plots in the main paper and SI).

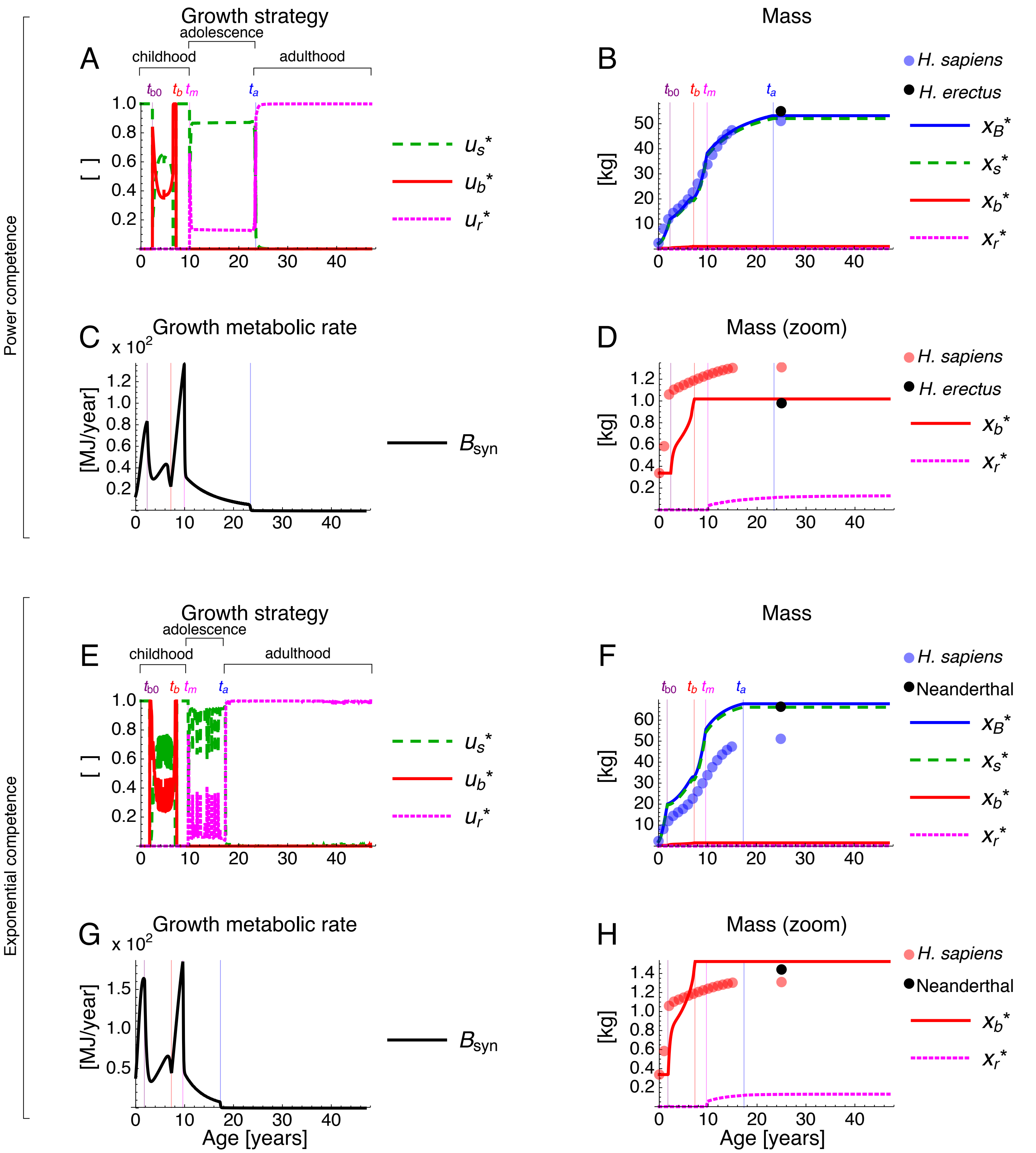

Figure E: Uninvadable growth strategy with fecundity given by Eq. (S67). The plots are the results as in Fig. 3 in the main text but with fecundity given by Eq. (S67).

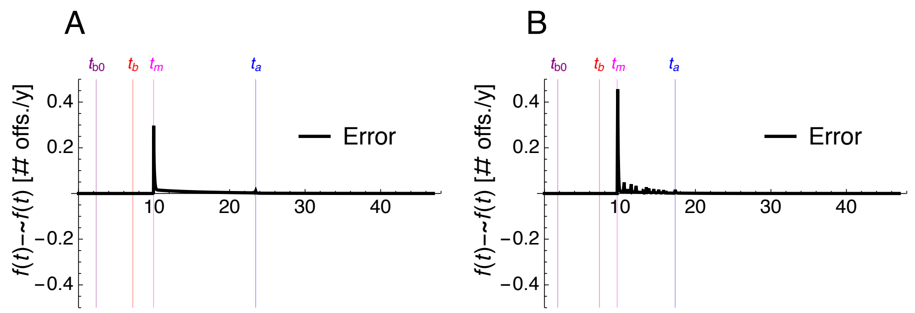

Figure F: Error in the approximated fecundity. The plots are the resulting fecundity when using Eq. (S67) minus the resulting fecundity when using Eq. (30) as in Fig. 3 in the main text. (A) The error for the power competence case. (B) The error for the exponential competence case.

8.2.2 Effect of removing maternal care

Fig. G shows the predicted growth patterns with the same situation of Fig. 3E-H in the main text but removing maternal care by setting the maternal facilitation at birth to zero, $\varphi_0 = 0$. The predicted adult body and brain mass are essentially unchanged, but there is a period of negative growth early in life as the individual has larger tissues than what it can maintain without maternal care (growth metabolic rate is negative early in life; Fig. (G panel D).

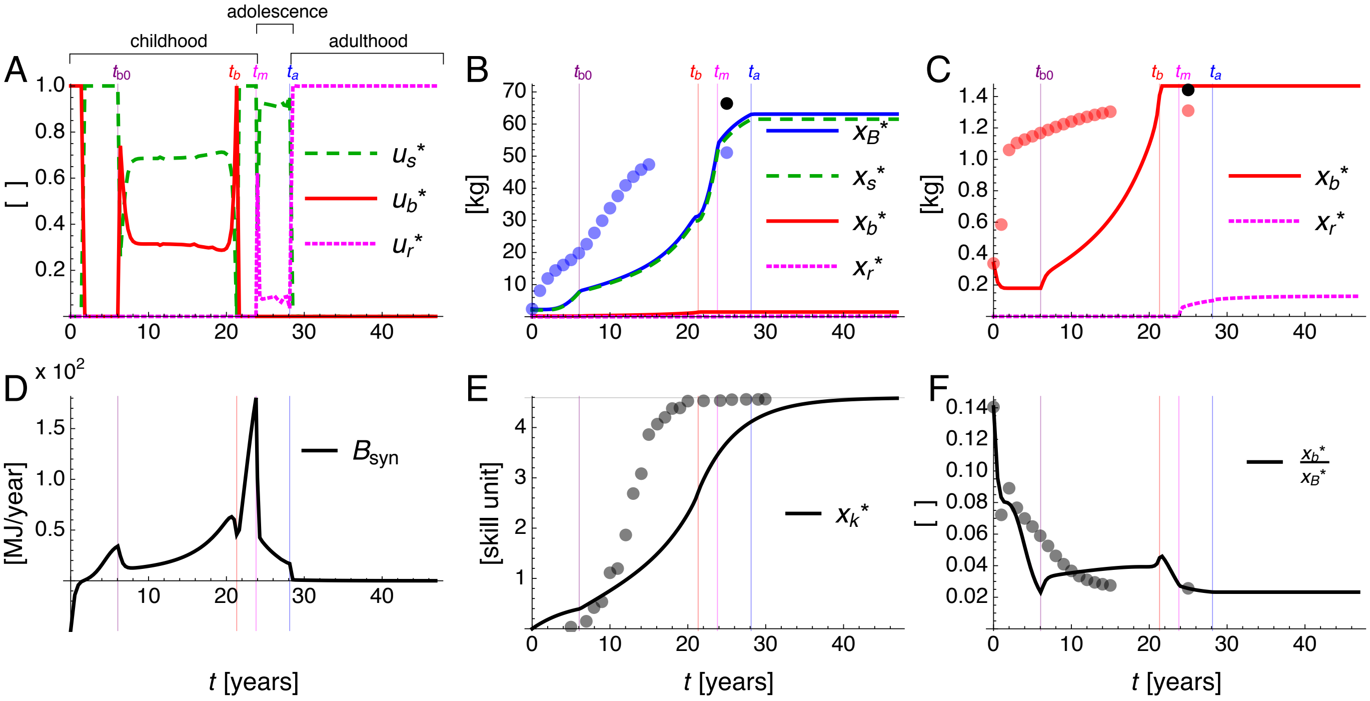

Figure G: Effect of removing maternal care with exponential competence. Parameters are as in Fig. 3E-H in the main text, except that here maternal care is absent; i.e., $\varphi_0 = 0$.

8.2.3 Relaxing the assumption that maternal care is independent of maternal skill

Here we relax the assumption that maternal care is independent of maternal skill by letting $\varphi(t) = \varphi_0 \exp(-\varphi_r t)$ (Eq. 33) be dependent on maternal skill. We do so by assuming that the facilitation given to a newborn (φ_0) depends on the skill level of its mother (whereas the rate φ_r of decrease of maternal care remains independent of maternal skill). Given clonal reproduction, the mother of a mutant individual is equally mutant. So, the skill level of the mutant's mother when the mother is of age t is $x_k(t)$. A newborn mutant may be born by mothers of different ages, and to determine the mutant's invasion fitness we need the average environment faced by the mutant [25–29]. Since the population is large, an average mutant is born by a mutant mother of average age. Hence, the maternal facilitation $\varphi_0(\bar{x}_k)$ received by an average mutant newborn depends on the average skill level of mutant mothers, which is

$$\bar{x}_k = \int_0^T x_k(t)\rho(t)\mathrm{d}t \tag{S68}$$

where the probability density function of mutant mothers of age t is [30]

$$\rho(t) = \frac{l(t)m(t)}{\int_0^T l(\tau)m(\tau)\mathrm{d}\tau}. \tag{S69}$$

From the definition of $\varphi(t)$, $\varphi_0(\bar{x}_k)$ must be between 0 and 1 so that the environmental difficulty $d(t)$ is non-negative. So, we take the maternal facilitation to a newborn as

$$\varphi_0(\bar{x}_k) = \frac{\bar{x}_k}{1+\bar{x}_k}, \tag{S70}$$

which is an increasing function of the maternal average skill level, starting from 0 and saturating at 1.

Using Eq. (S70), the obtained uninvadable growth strategy and resulting growth patterns are in Fig. H. The resulting facilitation to a newborn is $\varphi_0 = 0.8$ with power competence and $\varphi_0 = 0.82$ with exponential competence. Since in Fig. 3 in the main text we took $\varphi_0 = 0.6$ for power competence and $\varphi_0 = 0.8$ for exponential competence, Fig. H shows faster growth with power competence but is virtually identical for exponential competence. In both cases, the resulting adult body and brain mass are essentially unchanged.

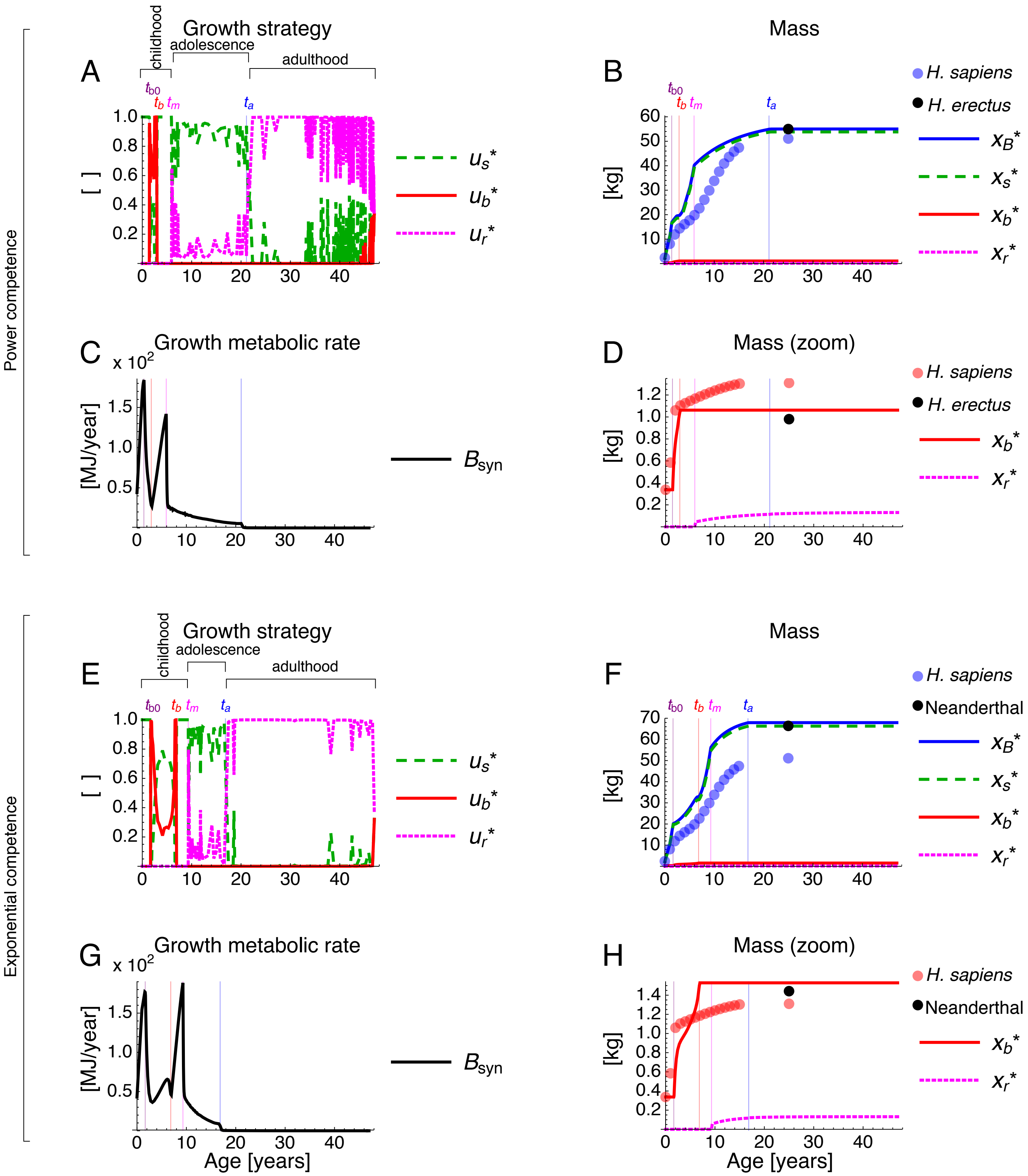

32 Figure H: Uninvadable growth strategy (u_i^*) and the resulting growth patterns (x_i^*) under a me-vs-nature set-
33 ting when maternal care depends on maternal skill. See legend of Fig. 3 in the main text.

8.3 Brain metabolic rate through ontogeny

With the obtained optimal growth strategy, brain metabolic rate is predicted to peak at the age of brain growth arrest, which is qualitatively consistent with recent findings for brain glucose intake (Fig. I panels A,B and Fig. J panels A,B; [15]). Brain metabolic rate and brain glucose intake are, however, not equivalent because the former refers to oxygen consumption while the latter includes non-oxidative glucose metabolism which is especially high during childhood [15, 17]. As observed with brain glucose intake [15], a peak in brain metabolic rate is predicted during mid childhood. The predicted small peak in brain metabolic rate results from brain growth arrest (Fig. I panel B and Fig. J panel B) and is enhanced by a peak in allocation to brain growth just before brain growth arrest (Fig. 3A,E in the main text). The predicted ratio of brain metabolic rate and resting metabolic rate is also qualitatively consistent with brain glucose intake in modern humans (Fig. I panel C and Fig. J panel C).

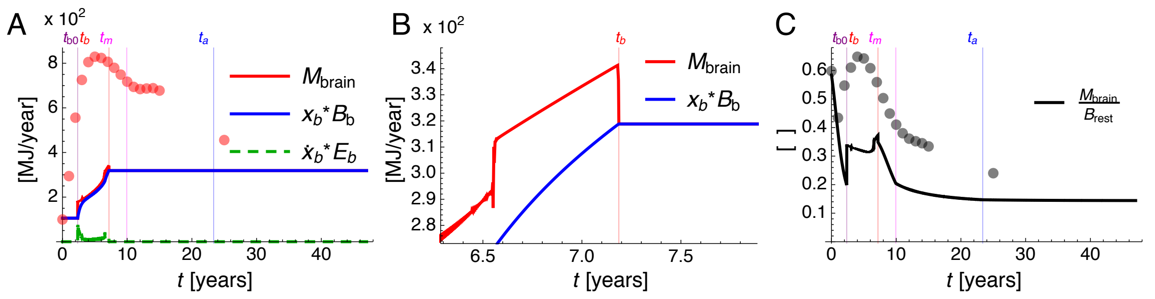

Figure I: Predicted and observed brain metabolic patterns in humans qualitatively agree. Plots are for the scenario in Fig. 3A-D in the main text (power competence). (A) Maintenance (blue; $x_{\mathrm{b}}^{*} B_{\mathrm{b}}$), growth (green; $\dot{x}_{\mathrm{b}}^{*} E_{\mathrm{b}}$), and total (red; $B_{\mathrm{rest,b}}$) brain metabolic rates. (B) Brain metabolic rate peaks at the age of brain growth arrest. (C) Ratio of brain metabolic rate to resting metabolic rate vs age. Dots are (A) the energy-equivalent brain glucose intake observed in modern human females or (C) the ratio of the latter to resting metabolic rate [15]. A similar pattern is predicted with exponential competence (Fig. J). Image filtering is not applied to this figure.

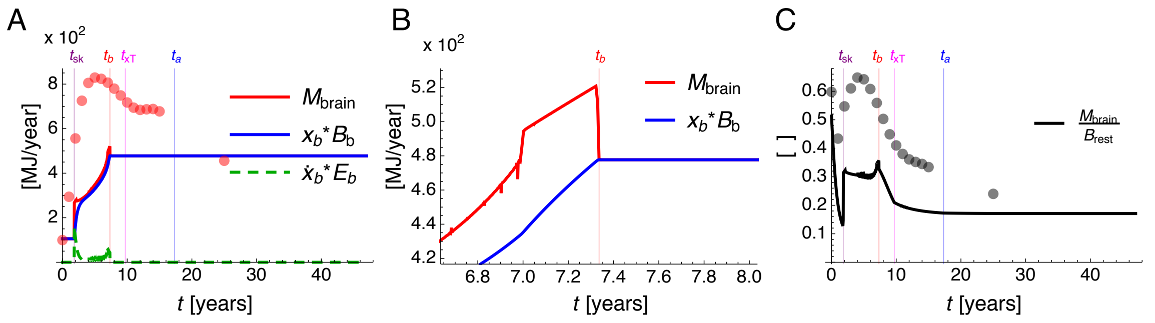

Figure J: Predicted brain metabolic patterns with exponential competence. Plots are for the scenario in Fig. 3E-H in the main text (exponential competence). See legend of Fig. I. Image filtering is not applied to this figure.

8.4 Mass of reproductive tissue

For the parameter values of Fig. 3 in the main text, the predicted reproductive tissue mass remains at zero until maturity t_m and reaches 129 g (with power competence) or 131 g (with exponential competence) during adulthood, exceeding the 3 g we roughly estimate for human females (SI §6.1).

8.5 Indeterminate skill growth with inexpensive memory

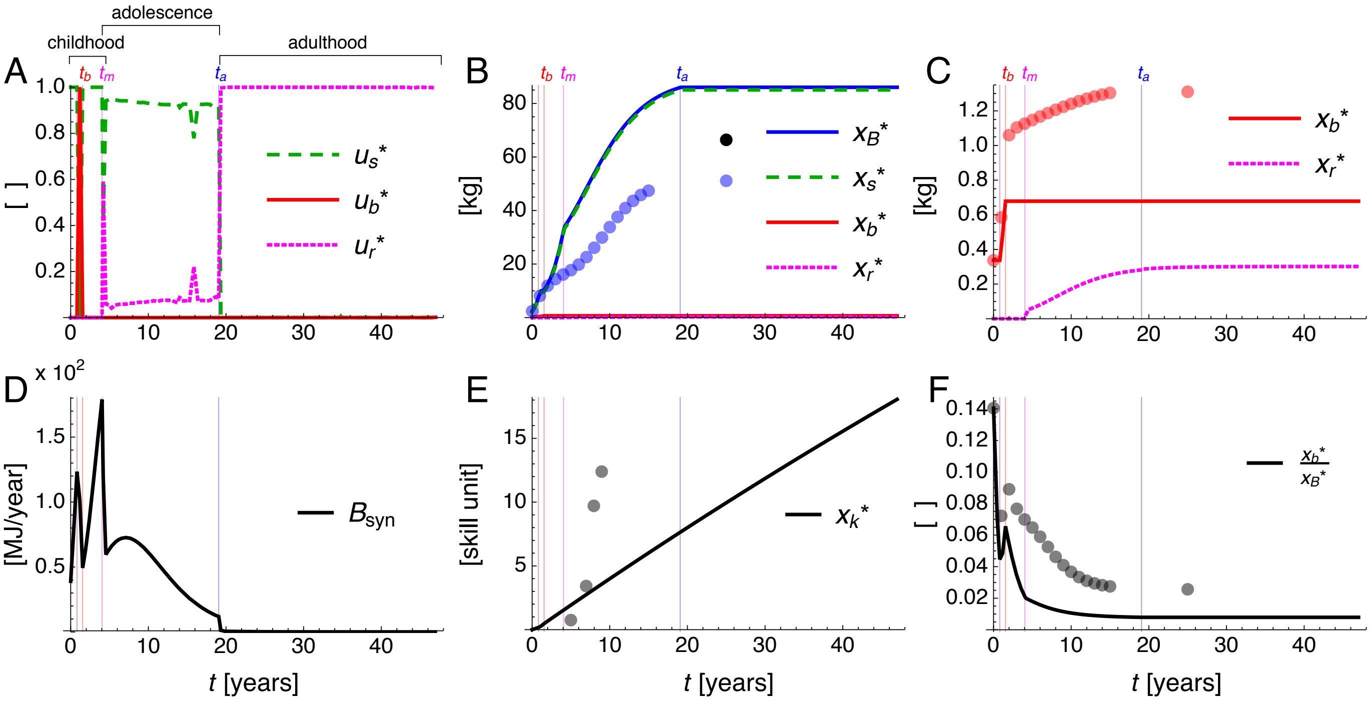

Figure K: Indeterminate skill growth with inexpensive memory and exponential competence. In E, skill level does not plateau. Parameters are as in Fig. 3E-H in the main text, except that here $B_k = 1$ MJ/y/skill rather than $B_k = 50$ MJ/y/skill.

8.6 Large, yet inconsistent-with-data encephalization with exceedingly expensive memory

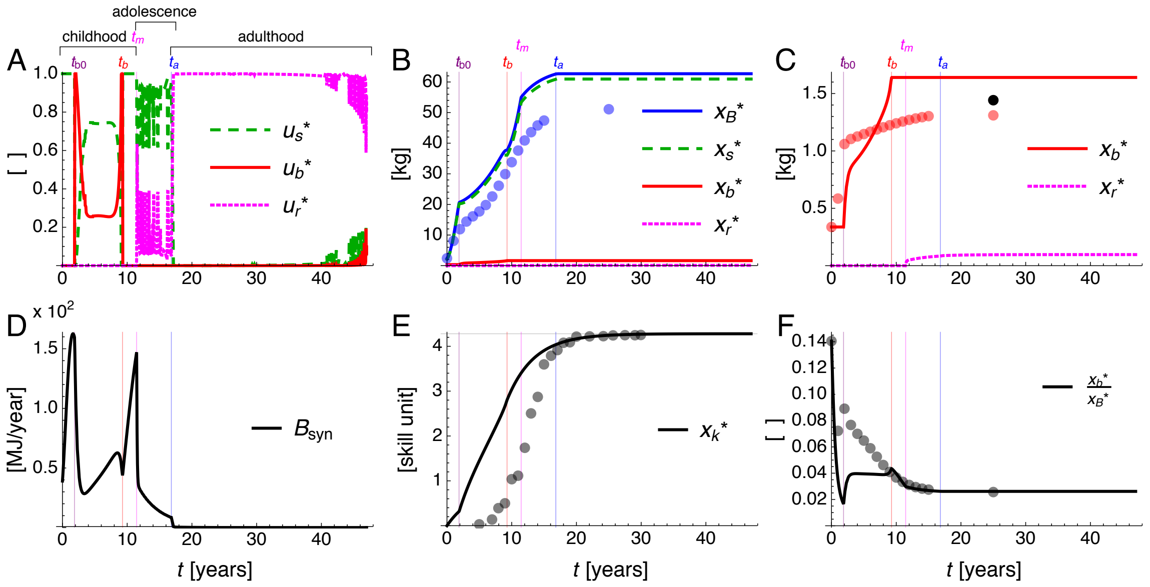

Figure L: Larger EQ than that in Fig. 3 in the main text with exponential competence, but predicted body mass is less consistent with observation. Parameters are as in Fig. 3E-H in the main text, except that here $B_k = 60$ MJ/y/skill rather than $B_k = 50$ MJ/y/skill.

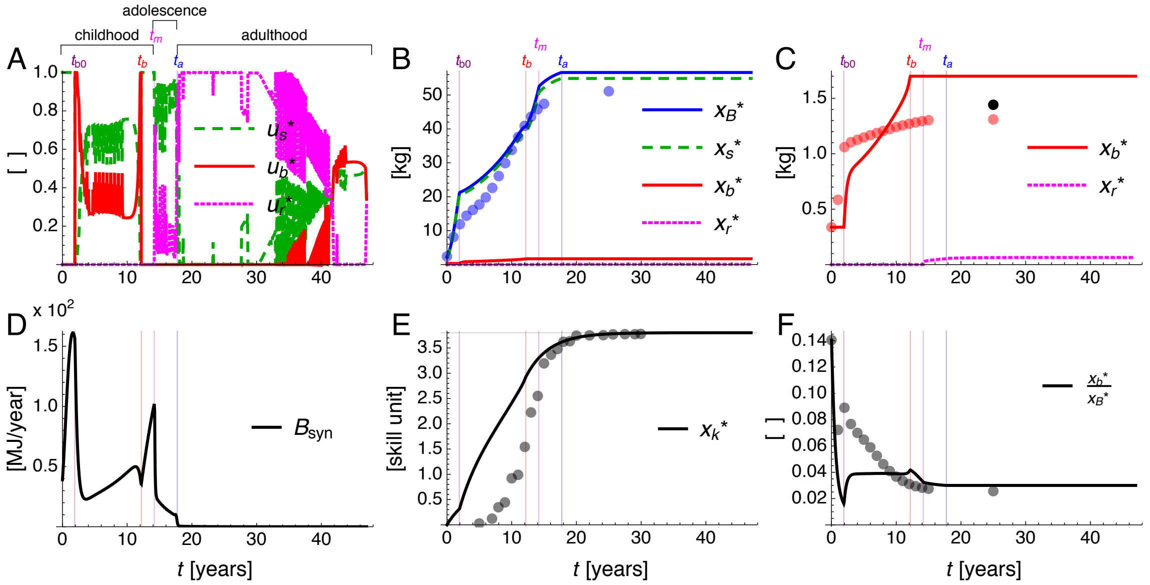

Figure M: Larger EQ than that in Fig. 3 in the main text with exponential competence, but predicted body mass is less consistent with observation. Parameters are as in Fig. 3E-H in the main text, except that here $B_k = 70$ MJ/y/skill rather than $B_k = 50$ MJ/y/skill.

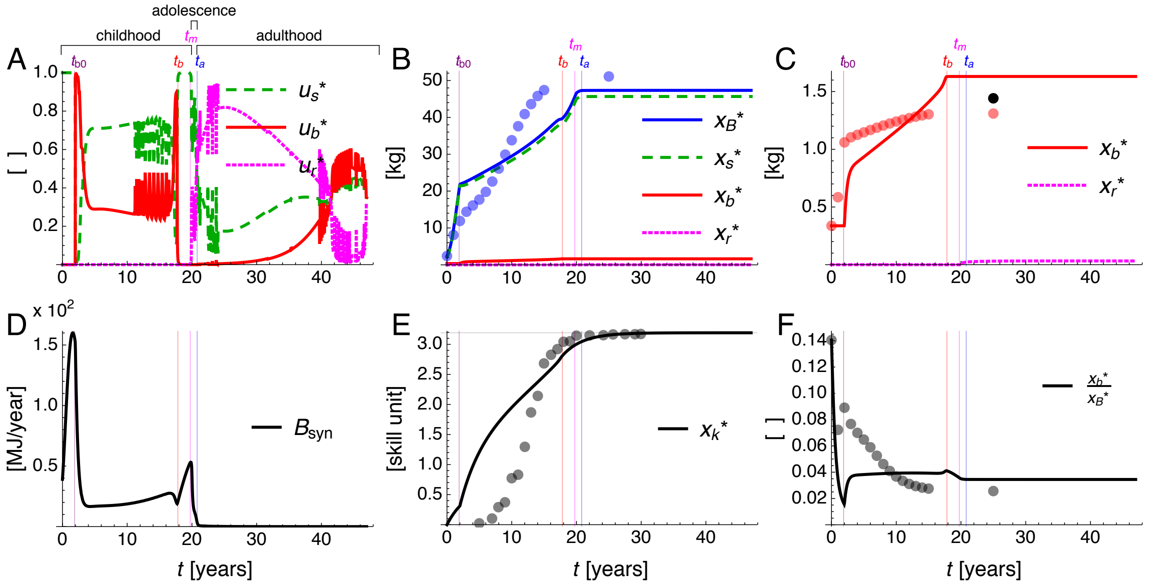

Figure N: Larger EQ than that in Fig. 3 in the main text with exponential competence, but predicted body mass is less consistent with observation. Parameters are as in Fig. 3E-H in the main text, except that here $B_{\mathrm{k}} = 80$ MJ/y/skill rather than $B_{\mathrm{k}} = 50$ MJ/y/skill.

8.7 Reproduction without growth and body collapse for certain parameter values

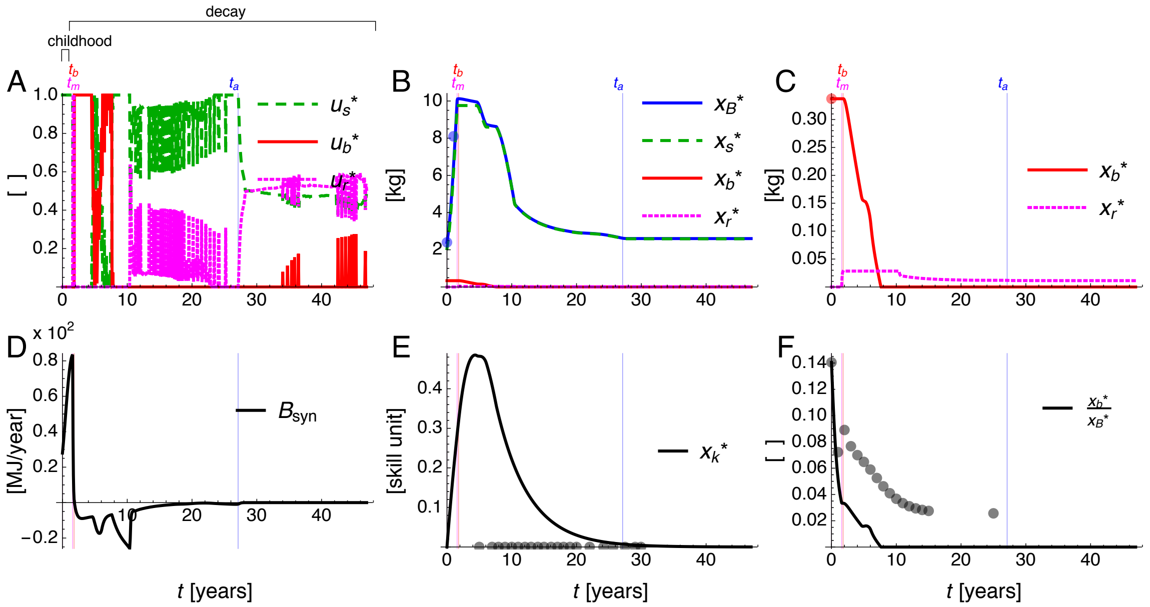

Figure O: Reproduction without substantial growth with exponential competence when the environment is exceedingly challenging. Parameters are as in Fig. 3E-H in the main text, except that here $\alpha = 1.5$ rather than 1.15. The mass of reproductive tissue grows from 0 kg at birth, to 0.77 g at the age of $t_{\rm m} \approx 6$ months, and reaches a peak of 4.64 g at $t_{\rm b} \approx 8$ months.

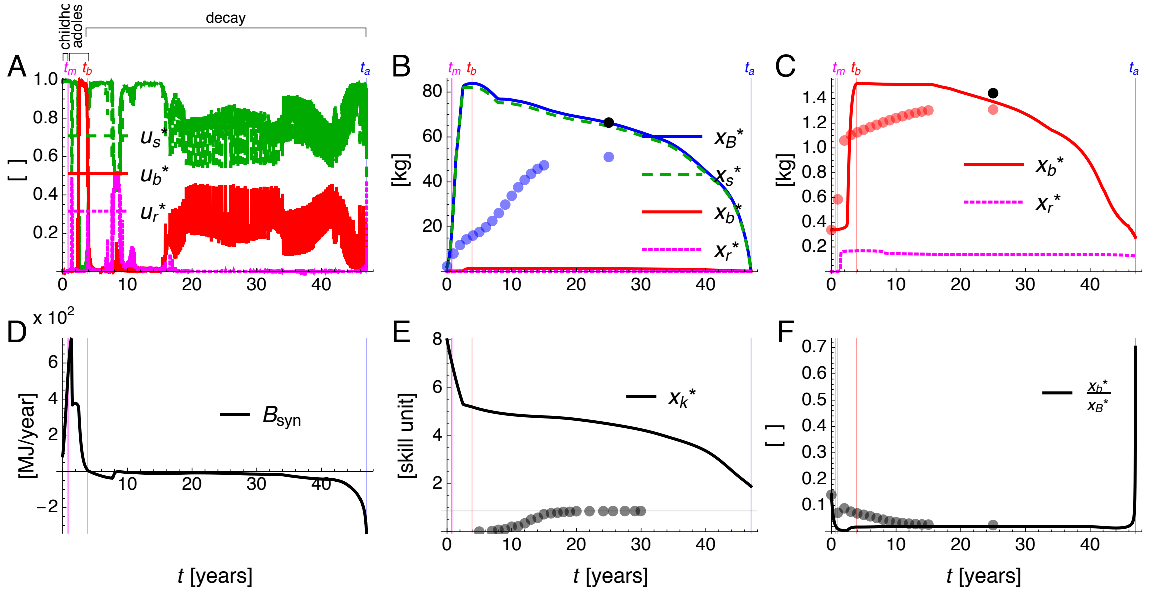

Figure P: Brain and body collapse with exponential competence when the newborn is overly skilled. Parameters are as in Fig. 3E-H in the main text, except that here $x_{\rm k}(0) = 8$ skill units rather than 0.

8.8 A large brain is also favored by small metabolic costs of learning, small innate skill, and intermediate allocation of brain metabolic rate to skills

In sections 8.8 and 8.9 we vary the values of the remaining parameters that were not estimated from data to assess their effect on the predicted switching times and adult body and brain mass.

We obtain that later ages at brain growth arrest, at maturity, and at adulthood are favored by costly learning, small skill at birth, and smaller brain allocation to skills (Fig. Q). Despite quantitative variation in the switching times, the sequence of childhood, adolescence, and adulthood remains (Fig. Q).

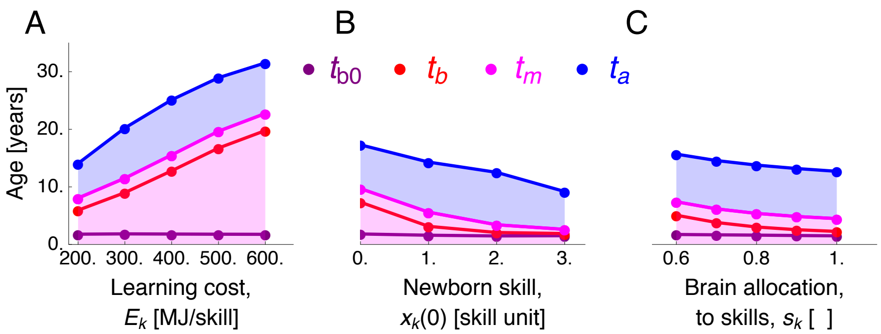

Figure Q: Predicted ages at brain growth onset, brain growth arrest, maturity, and adulthood vs other parameter values with exponential competence. See legend of Fig. 5 in the main text.

Adult brain and body mass decrease with increasing learning costs, and with exceedingly expensive learning, body and brain mass fail to grow (Fig. R panel A). This is because with increasingly expensive learning, skill grows more slowly and thus there is less growth metabolic rate at each age. Similarly, adult body mass decreases with increasing learning costs. Consequently and in contrast with memory costs (Figs. 5C and 6C in the main text), while increasing learning costs delay the ages at maturity and brain growth arrest (Fig. Q panel A), learning costs do not affect EQ within the range where adult brain and body mass are substantially greater than zero (Fig. R panel D).

With respect to newborn skill level, larger adult brain and body mass are predicted when the newborn has small skill (Fig. R panel B). If the newborn is overly skilled, the individual grows more during the maternal care period than what it can maintain when maternal care is absent, causing brain and body collapse during adulthood (Fig. R panel B and Fig. P).

With respect to allocation of brain metabolic rate to energy-extraction skills, brain mass is predicted to be larger with a decreasing, but not exceedingly, small brain allocation to skills (Fig. R panel C). With an exceedingly small brain allocation to skills, the individual reproduces without substantial growth because skill grows little and the individual is unable to support itself when maternal care becomes absent. Above a threshold, an increasing brain allocation to skills predicts a decreasing adult brain mass because the energetic input to skill growth is larger without the brain having to be as large (Eq. 24b). In contrast to brain mass and EQ, the predicted adult skill level increases with brain allocation to skills (Fig. R panel F).

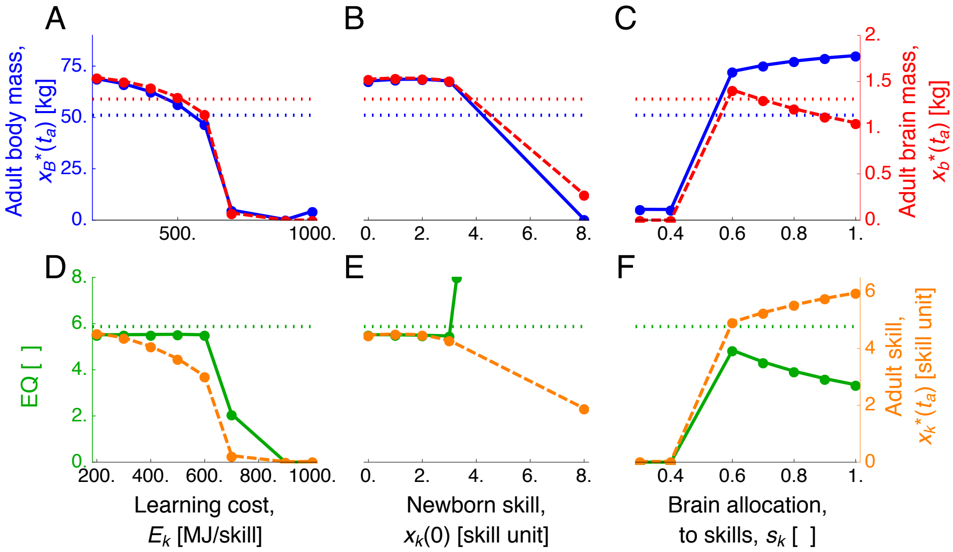

Figure R: Predicted adult body and brain mass, EQ, and skill vs other parameter values with exponential competence. See legend of Fig. 6 in the main text.

Comparative predictions with power competence are similar to those with exponential competence (Figs. S and T).

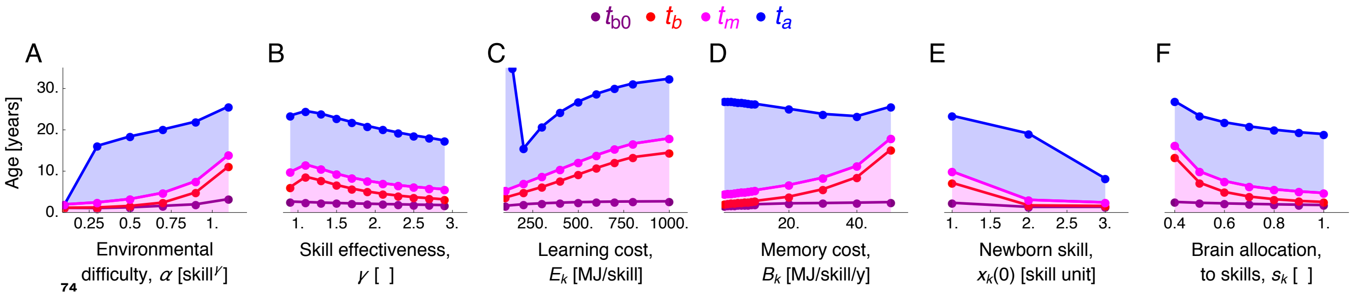

Figure S: Predicted ages at brain growth onset, brain growth arrest, maturity, and adulthood vs other parameter values with power competence. See legend of Fig. 5 in the main text.

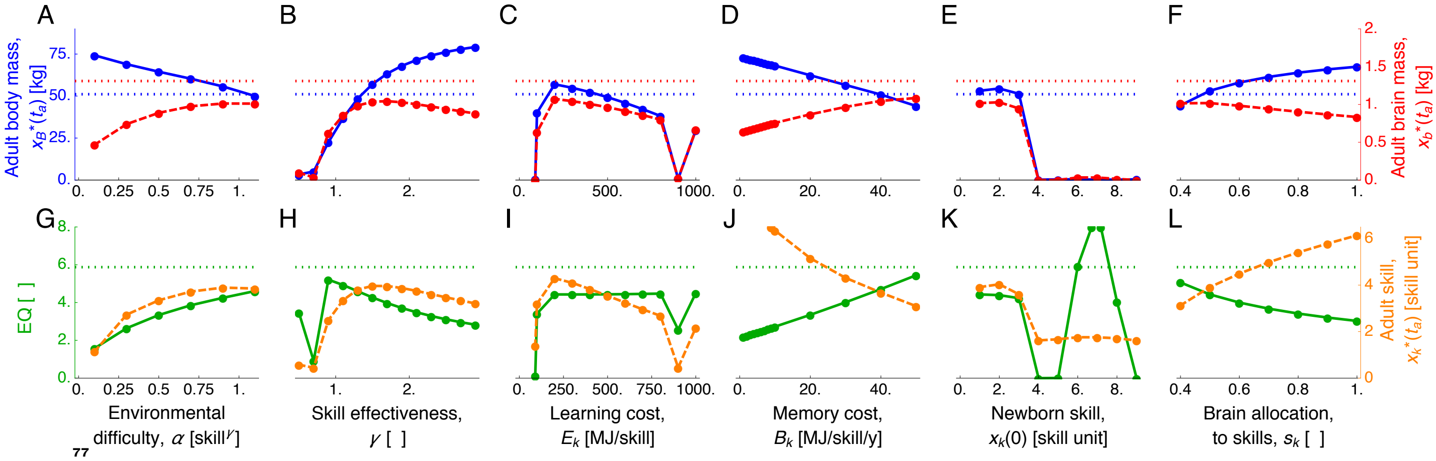

Figure T: Predicted comparative patterns with power competence. See legend of Fig. 6 in the main text. In (A,G) adult body and brain mass also collapse for exceedingly large environmental difficulty but in this case the error tolerance is not satisfied so the point is not shown. In (H,K) jitter in EQ is due to near zero adult body mass. In (I) the point at $E_\mathrm{k} = 900$ MJ/skill may be due to the solver stalling near a minimum (the objective is ≈ 0.6).

8.9 Dependence on maternal care parameters

Finally, we vary the parameter values controlling maternal care to assess their effect on predictions. We find that later ages at brain growth arrest, at maturity, and at adulthood are favored by decreasing maternal facilitation for newborns and by faster decrease in maternal care (Fig. U). That is, maternal care allows for earlier switching times.

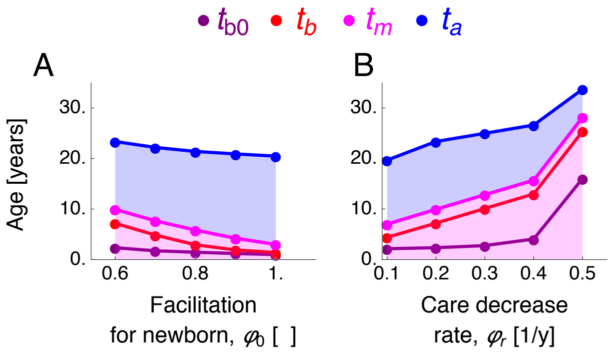

Figure U: Predicted ages at brain growth onset, brain growth arrest, maturity, and adulthood vs maternal care parameter values with power competence. See legend of Fig. 5 in the main text.

Maternal care parameters have minor effects on the adult body and brain mass attained (Fig. V). Such invariance is likely to vanish if maternal care is allowed to affect newborn mortality, which we did not consider for simplicity.

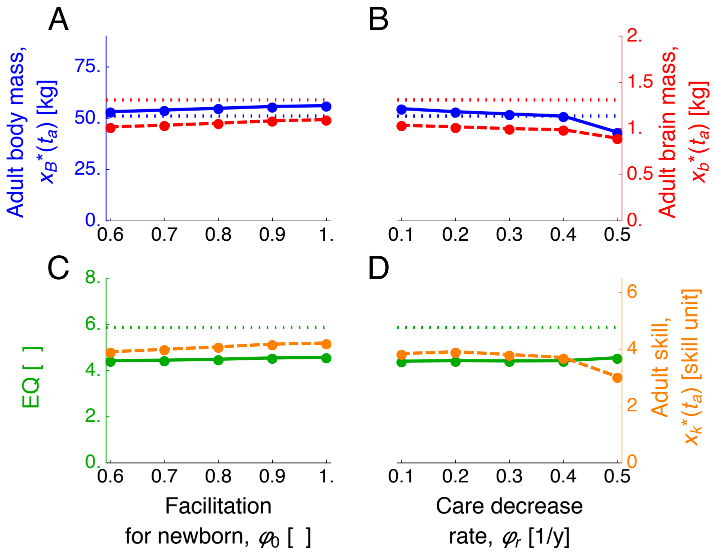

Figure V: Predicted adult body and brain mass, EQ, and skill vs maternal care parameter values with power competence. See legend of Fig. 6 in the main text.

Similar results for the effects of maternal care are obtained with either power or exponential competence (Figs. W and X). In sum, maternal care only triggers earlier switching times in the model.

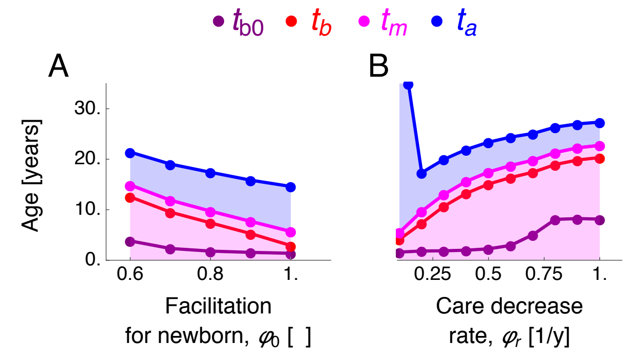

Figure W: Predicted ages at brain growth onset, brain growth arrest, maturity, and adulthood vs maternal care parameter values with exponential competence. See legend of Fig. 5 in the main text.

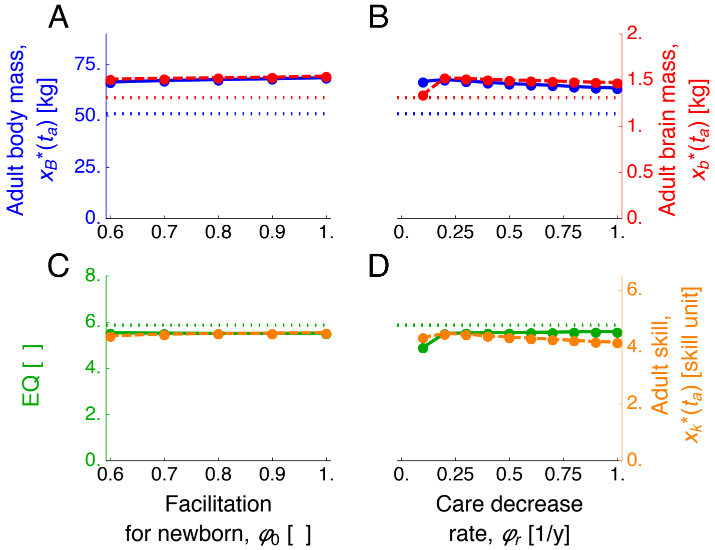

Figure X: Predicted adult body and brain mass, EQ, and skill vs maternal care parameter values with exponential competence. See legend of Fig. 6 in the main text.

References

[1] King D, Roughgarden J. Graded allocation between vegetative and reproductive growth for annual plants in growing seasons for random length. Theor Popul Biol. 1982;22:1–16.

[2] Iwasa Y, Roughgarden J. Shoot/root balance of plants: optimal growth of a system with many vegetative organs. Theor Popul Biol. 1984;25:78–105.

[3] Perrin N. Optimal resource allocation and the marginal value of organs. Am Nat. 1992;139:1344–1369.

[4] Irie T, Iwasa Y. Optimal growth pattern of defensive organs: the diversity of shell growth among mollusks. Am Nat. 2005;165:238–249.

[5] Sydsæter K, Hammond P, Seierstad A, Strom A. Further Mathematics for Economic Analysis. 2nd ed. Prentice Hall; 2008.

[6] Bryson, Jr AE, Ho YC. Applied Optimal Control. Taylor & Francis; 1975.

[7] Kamien MI, Schwartz NL. Dynamic Optimization. 2nd ed. Mineola, NY: Dover; 2012.

[8] Dorfman R. An economic interpretation of optimal control theory. Am Econ Rev. 1969;59:817–831.

[9] Hartl RF, Sethi SP, Vickson RG. A survey of the maximum principles for optimal control problems with state constraints. SIAM review. 1995;37:181–218.

[10] Patterson MA, Rao AV. GPOPS-II: A MATLAB Software for solving multiple-phase optimal control problems using hp-adaptive Gaussian quadrature collocation methods and sparse nonlinear programming. ACM Trans Math Softw. 2014;41:1–37.

[11] Diehl M, Bock HG, Diedam H, Wieber PB. Fast direct multiple shooting algorithms for optimal robot control. In: Fast motions in biomechanics and robotics. Berlin: Springer; 2006. p. 65–93.

[12] Kelley HJ, Kopp RE, Moyer HG. Singular extremals. In: Leitmann G, editor. Topics in Optimization. New York: Academic Press; 1967. p. 63–101.

[13] Moses ME, Hou C, Woodruff WH, West GB, Nekola JC, Zuo W, et al. Revisiting a model of ontogenetic growth: estimating model parameters from theory and data. Am Nat. 2008;171:632–645.

[14] Schniter E, Gurven M, Kaplan HS, Wilcox NT, Hooper PL. Skill ontogeny among Tsimane forager-horticulturalists. Am J Phys Anthrop. 2015;158:3–18.

[15] Kuzawa CW, Chugani HT, Grossman LI, Lipovich L, Muzik O, Hof PR, et al. Metabolic costs and evolutionary implications of human brain development. Proc Nat Acad Sci USA. 2014;111:13010–13015.

[16] West GB, Brown JH, Enquist BJ. A general model for ontogenetic growth. Nature. 2001;413:628–631.

[17] Goyal MS, Hawrylycz M, Miller JA, Snyder AZ, Raichle ME. Aerobic Glycolysis in the Human Brain Is Associated with Development and Neotenous Gene Expression. Cell Metab. 2014;19:49–57.

[18] Magnusson C, Hillensjö T, Hamberger L, Nilsson L. Oxygen consumption by human oocytes and blastocysts grown in vitro. Hum Reprod. 1986;1:183–184.

[19] Blaxter K. Energy Metabolism in Animals and Man. Cambridge, UK: Cambridge Univ. Press; 1989.

[20] Abramczuk J, Sawicki W. Variation in dry mass and volume of nonfertilized oocytes and blastomeres of 1-, 2- and 4-celled mouse embryos. J Exp Zool. 1974;188:25–34.

[21] Dickey RP, Taylor SN, Lu PY, Sartor BM, Rye PH, Pyrzak R. Effect of diagnosis, age, sperm quality, and number of preovulatory follicles on the outcome of multiple cycles of clomiphene citrate-intrauterine insemination. Reproductive Endocrinology. 2002;78:1088–1095.

[22] O'Herlihy C, De Crespigny LC, Lopata A, Johnston I, Hoult I, Robinson H. Preovulatory follicular size: a comparison of ultrasound and laparoscopic measurements. Fertil Steril. 1980;34:24–26.

[23] Gurven M, Kaplan H. Longevity among hunter-gatherers: a cross-cultural examination. Popul Dev Rev. 2007;33:321–365.

[24] Eaton SB, Pike MC, Short RV, Lee NC, Trussell J, Hatcher RA, et al. Women's reproductive cancers in evolutionary context. Q Rev Biol. 1994;69:353–367.

[25] Metz JAJ, Geritz SAH, Meszéna G, Jacobs FJA, van Heerwaarden JS. Adaptive Dynamics, a geometrical study of the consequences of nearly faithful reproduction. In: van Strien SJ, Verdyan Lunel SM, editors. Stochastic and Spatial Structures of Dynamical Systems. Amsterdam, The Netherlands: North Holland; 1996. p. 183–231.

[26] Dieckmann U, Heino M, Parvinen K. The adaptive dynamics of function-valued traits. J Theor Biol. 2006;241:370–389.

[27] Parvinen K, Heino M, Dieckmann U. Function-valued adaptive dynamics and optimal control theory. J Math Biol. 2013;67:509–533.

[28] Metz JAJ, Staňková K, Johanson J. The canonical equation of adaptive dynamics for life histories: from fitness-returns to selection gradients and Pontryagin's maximum principle. J Math Biol. 2016;72:1125–1152.

[29] Metz JAJ, Geritz SAH. Frequency dependence 3.0: an attempt at codifying the evolutionary ecology perspective. J Math Biol. 2016;72:1011–1037.

[30] Charlesworth B. Evolution in age-structured populations. 2nd ed. Cambridge Univ. Press; 1994.
